# Deciphering the potential ability of DExD/H-box helicase 60 (DDX60) on the proliferation, diagnostic and prognostic biomarker in pancreatic cancer: a research based on silico, RNA-seq and molecular biology experiment

**DOI:** 10.1186/s41065-024-00361-9

**Published:** 2025-01-22

**Authors:** Dongdong Zhang, Enze Zhang, Ying Cai, Yixin sun, Peiji Zeng, Xiaohua Jiang, Yifan Lian

**Affiliations:** 1 Department of Gastroenterology, Zhongshan Hospital of Xiamen University, School of Medicine, Xiamen University, Xiamen, China; 2 School of Medicine, Xiamen University, Xiamen, 361000 Fujian China; 3 3National Institute for Data Science in Health and Medicine, Xiamen UniversityXiamen, Fujian, 361000 China; 4 Department of Orthopedics, Xiang’an Hospital of Xiamen University, School of Medicine, Xiamen University, Xiamen, Fujian People’s Republic of China

**Keywords:** DDX60, RNA-seq, Pancreatic cancer; prognostic, TCGA, GEO, Biomarker

## Abstract

**Background:**

Pancreatic cancer is one of the most malignant abdominal tumors. DDX60 has been shown to be associated with a variety of tumor biological processes. However, DDX60 in pancreatic cancer has not been reported. Our study confirmed that DDX60 can serve as a novel biomarker for diagnosis and treatment of pancreatic cancer.

**Materials and methods:**

We downloaded pancreatic cancer datasets from GEO and TCGA databases, respectively. To investigate the relationship between DDX60 expression and prognosis in pancreatic cancer. GSEA analysis was performed on DDX60. We performed RNA-seq to further explore the downstream biological targets of DDX60 and the signaling pathways that may be involved in pancreatic cancer. Finally, we tested it through molecular biology experiments. First, we constructed the plasmid and tested the plasmid effect by WB. Then MTT assay was performed to explore the effect of DDX60 knockout on the proliferation of pancreatic cancer cells. LDH assay was performed to explore the effect of DDX60 on the release of lactate dehydrogenase from tumor cells. The effect of DDX60 on cell proliferation was further explored by clonal formation experiment. Continue to explore clinical therapeutic drugs sensitive to DDX60 targets.

**Results:**

By analyzing the GSE71729, GSE183795, GSE16515, GSE28735 and GSE62452 data sets, we found that DDX60 was highly expressed in pancreatic cancer. And is associated with poorer outcomes for pancreatic patients. The mRNA expression level of DDX60 was correlated with lymph node metastasis and grade in clinical pancreatic patients. Through the results of RNA-seq analysis, GO and KEGG analysis showed that DDX60 may be associated with cell cycle in pancreatic cancer. Through molecular biology experiments (MTT, LDH, and clonal formation experiment), we found that When DDX60 is knocked down in pancreatic cancer cells, the proliferation ability of tumor cells is significantly decreased. Several drugs targeting about DDX60 have been found, such as JW-7–52-1, this could provide direction for drug therapy against the DDX60 target.

**Conclusion:**

In summary, DDX60 can be used as a novel biomarker related to the diagnosis and treatment of pancreatic cancer, participate in tumor proliferation, and is associated with poor prognosis in patients.

**Supplementary Information:**

The online version contains supplementary material available at 10.1186/s41065-024-00361-9.

## Introduction

Pancreatic cancer is one of the most aggressive malignancies within the digestive system [1, 2]. In 2023, there were 64,050 new cases reported, with 33,130 being male and 30,920 female. Unfortunately, the disease claimed 50,550 lives, affecting 26,620 men and 23,930 women [3]. Despite advancements in treatment methods such as surgery, chemotherapy, and radiotherapy, the prognosis for patients with pancreatic cancer remains poor [4], and there is a significant risk of recurrence and metastasis following surgical intervention [5, 6]. The challenge of improving patient outcomes has prompted a search for novel biomarkers that could aid in the early diagnosis and treatment of pancreatic cancer. This quest is part of a broader shift in scientific research, which now leverages a variety of technological approaches beyond traditional molecular biology experiments.

Technological advancements in genomics [7], proteomics [8], and radiomics [9, 10] have become integral to cancer research. Genomic changes are at the root of cancer development, and while molecular biology has provided insights into signaling mechanisms, the advent of genome sequencing has offered a more detailed and comprehensive view of tumor progression. The emergence of new sequencing technologies has clarified our understanding of the genome, providing a foundation for identifying the genetic underpinnings of pancreatic cancer [11]. Our research aims to harness this genomic information by analyzing data from public databases. By doing so, we seek to uncover potential therapeutic targets in pancreatic cancer. This approach is crucial for developing new diagnostic and treatment strategies that could ultimately enhance the prognosis and survival rates of patients with pancreatic cancer.

There are various ways of tumor treatment, among which immunotherapy is a novel treatment in recent years [12–14]. Factors such as immune checkpoint [15] and immunosuppressant [16] are involved in the process of immunotherapy. Tumor immune microenvironment is involved in the metastasis of pancreatic cancer. Due to the difficulty in the early diagnosis of pancreatic cancer, it is very important to study tumor immune microenvironment for the treatment of pancreatic cancer [17]. Therefore, it is more important to explore the infiltration scores of immune cells and mechanism cells in pancreatic cancer. These studies could create opportunities for immunotherapy in clinical pancreatic cancer patients, and thus roughly identify the patient population that could benefit from immunotherapy clinically.

At the same time, DDX60 can also act as a defender of the cellular antiviral response [18]. In recentyears, it has been reported that DDX60 is involved in the occurrence and developmentof tumors. Zaixiang Tang's team reported that DDX60 was associated with breast cancer and that patients in the low-risk DDX60 group had better sensitivity to chemotherapy [19]. Therefore, we raised questions about whether DDX60 can affect the chemotherapy sensitivity of pancreatic cancer, and we also conducted preliminary exploration in our own study. In addition, DDX60 has been reported to be associated with autoimmune diseases, such as systemic lupus erythematosus. DDX60 is associated with serum activity in patients with systemic lupus erythematosus and may serve as a therapeutic biomarker [20]. Studies on DDX60 in pancreatic cancer have not yet been reported. Our study aims to explore the relationship between DDX60 and prognosis, immunity, chemotherapy sensitivity, and cell proliferation in patients with pancreatic cancer.

## Materials and methods

### Data acquire and patients collection

The original data of gene expression profile and patients data related to pancreatic adenocarcinoma (PAAD) were obtained from TCGA-PAAD database [21], which including transcriptome sequencing data (count) and clinical data (xml) of PAAD patients. A total of 183 reserch patients information (179 tumor cohorts, 4 normal cohorts) were downloaded, and the clinical information (tumor size and depth of invasion, age, gender, grade, stage, lymph node status, distant metastasis) of 183 reserch patients were also identified and obtained from the TCGA database. Excluding patients without the information of survival time and survival status. We input the “pancreatic” keywords in the GEO database [22] and collected GSE71729 [23], GSE183795 [24], GSE16515 [25–27], GSE28735 [28, 29] and GSE62452 [30] as validation datasets. The PAAD gene expression profile (GSE71729) was downloaded from the GEO database which was executed with help of GPL20769. The clinical information of GSE71729 was identified from the GEO database. GSE71729 contains a total of 357 samples, including 191 samples of pancreatic tissue. GSE71729 contains 46 non-tumor samples and 145 pancreatic ductal adenocarcinoma samples (Street address: 450 West Drive; Organization name: University of North Carolina; City: Chapel Hill; ZIP/Postal code: 27,599; Country: USA). The PAAD gene expression profile (GSE183795) was downloaded from the GEO database (http://www.ncbi.nlm.nih.gov/geo/), which was executed with the help of GPL6244. The clinical information of GSE183795 was identified from the GEO database. GSE183795 contains 105 non-tumor samples and 139 pancreatic tumor samples (Street address: 37 Convent Drive; Organization name: NIH/NCI; City: Bethesda; ZIP/Postal code: 20,892; Country: USA). The PAAD gene expression profile (GSE16515) was downloaded from the GEO database (http://www.ncbi.nlm.nih.gov/geo/), which was executed with help of GPL570. The clinical information of GSE16515 was identified from the GEO database. GSE16515 contains 16 non-tumor samples and 36 pancreatic tumor samples (Street address: 200 First street SW; Organization name: Mayo Clinic; City: Rochester; ZIP/Postal code: 55,905; Country: USA; Department:Molecular Pharmacology and Experimental Therapeutics). The pancreatic ductal adenocarcinoma gene expression profile (GSE28735) was downloaded from the GEO database (http://www.ncbi.nlm.nih.gov/geo/), which was executed with help of GPL6244. The clinical information of GSE28735 was identified from the GEO database. GSE28735 contains 45 non-tumor samples and 45 pancreatic tumor samples (Street address: 37 Convent Drive; Organization name: NCI/NIH; Contact name: Perwez Hussain; Submission date: Apr 20, 2011). The pancreatic ductal adenocarcinoma gene expression profile (GSE62452) was downloaded from the GEO database, which was executed with help of GPL6244. The clinical information of GSE62452 was identified from the GEO database. GSE62452 contains 61 non-tumor samples and 69 pancreatic tumor samples (Street address: 37 Convent Drive, Building 37, Room3044B; Organization name: National Cancer Institute; Lab: Laboratory of Human Carcinogenesis; Contact name: Perwez S Hussain; Submission date:Oct 17, 2014). The details information of five microarray datasets are shown in Table 1. The number of pancreatic tissue and the number of normal tissue were included for each dataset.

**Table 1:** Details of the GEO datasets included in this study

GSE Datasets	Normal (sample)	PAAD (sample)	Total	GPL	Organization name	City	ZIP/Postal code	Country
GSE71729	46	145	191	GPL20769	University of North Carolina	Chapel Hill	27,599	USA
GSE183795	105	139	244	GPL6244	NIH/NCI	Bethesda	20,892	USA
GSE16515	16	36	52	GPL570	Mayo Clinic	Rochester	55,905	USA
GSE28735	45	45	90	GPL6244	NCI/NIH	Bethesda	20,892	USA
GSE62452	61	69	130	GPL6244	National Cancer Institute	Bethesda	20,892	USA

### Data preprocessing and identification of DEGs

We download the Series Matrix File(s) and SOFT formatted family file(s) of the five microarray datasets from the GEO database. GSE71729、GSE183795、GSE28735、GSE62452 are in the format of RMA algorithm [31, 32], which directly downloaded from the official website GEO database. The GSE16515 dataset is in the format of MAS5 algorithm [33]. We download the GSE16515 information in MAS5 format, and then use the “Affy” packet [34, 35] to standardize the data and convert the data into RMA format. Finally, the R language is used to call the “limma” package [36] to analyze the microarray data. The probe ID istransformed into gene ID, and the gene ID is transformed into gene symbol. The "limma" package in Rstudio is used to identifies DEGs between pancreatic cancer tumor samples and healthy subjects or samples adjacent to cancer. We set the threshold as Adjust. *P*. Value < 0.05 and |Log2 Fold Change|≥ 0.7.

### GO and KEGG analysis

GO analysis includes BP, CC and MF. GO analysis is to obtain the biological processes that these genes may participate in by enriching multiple genes, and then to know the biological processes that the tumor may participate in. KEGG analysis belongs to pathway enrichment analysis, which can link multiple genes with advanced functions. In this study, we used DAVID online database for GO and KEGG analysis [37].

### DExD/H-box RNA Helicase mRNA Expression in GSE71729、GSE183795、GSE16515、GSE28735、GSE62452 and GEPIA database

We intersected DExD/H-box RNA helicase family related genes in five datasets and finally obtained DDX60. Here we explore the role of DDX60 in pancreatic cancer. We download the microarray representation matrix data from the platform file. The data of the chip is analyzed through the through “limma” package and “R” program [38, 39]. Then the expression of DDX60 in normal subject group and pancreatic tumor was extracted from silicos to calculate whether it was statistically significant. The paired sample T test method is used to first test whether the data conforms to the normal distribution. Four statistical methods were selected for analysis, and the data were all normal distribution GSE71729, GSE183795, GSE16515, and GSE62452. Independent sample T test was used to check whether the variances were homogeneous. Different statistical analyses were performed according to the results. Graphpad Prism8 is used for plotting. The GEPIA database [40] contained 179 patients with pancreatic tumors and 171 normal subjects. The GEPIA database was used to verify the mRNA expression of DDX60 in pancreatic cancer and normal subject groups.

### Analysis of protein expression of DDX60 in pancreatic tissue and pancreatic cancer

The CPTAC database [41] stores a lot of sequencing data of tumor tissues and adjacent tissues. It is exciting that this database is a public free database that researchers can access and download relevant resources at any time. Proteins are the main executors of biological functions. We use CPTAC database to explore the differential expression of DDX60 in pancreatic cancer tissues and adjacent tissues, and then explore the role of DDX60 in pancreatic cancer.

### Survival analysis of DDX60 in pancreatic cancer

We downloaded the sequencing data of STAR-Counts format from the TCGA database and used the “Perl” [42] and “R” language to process the transcriptome downstream data. According to the median value of DDX60, all pancreatic cancer patients included in the research were divided into high-risk group (DDX60 relatively high expression) and low-risk group (DDX60 relatively low expression). Analyze whether the prognosis of patients in the high-risk group and the low-risk group is different. *P* < 0.05 was statistically significant. The data of pancreatic cancer patients in UALCAN database [43] were used to verify the survival difference of DDX60 in high and low risk groups. The prognostic value (OS: overall survival and RFS: progression free survival) of DDX60 in 1, 3, and 5 years was verified using pancreatic cancer data in the Kaplan Meier database [44, 45].

### The relationship between DDX60 and clinical variables in pancreatic cancer

We used TCGA-PAAD data for clinical variable correlation analysis. The clinical features of patients were extracted from the TCGA-PAAD database. According to the variable name age, gender, lymph node metastasis, distant metastasis factors, two groups were formed. The patients were divided into 4 groups according to stage, grade, tumor size and depth of myometrial invasion. The next step is to analyze the expression differences of DDX60 in different clinical features.

#### GSEA

In order to further explore the molecular mechanism of target genes and tumor occurrence and development, we performed GSEA analysis [46]. GSEA analysis divided the samples into high-risk group and low-risk group according to the expression of the target gene, and calculated which biological pathways each group was enriched in. The purpose of this study was to explore the molecular mechanism of DDX60 in pancreatic cancer. The data packets used were “limma”, “org.Hs.eg.db”, “clusterProfiler” [47], “enrichplot”. Download the c2.cp.kegg.v7.4.symbols.gmt gene set as a reference.

### Drug sensitive

At present, in clinical work, the occurrence of drug resistance in tumor patients during the comprehensive treatment of tumors often occurs. Therefore, it is particularly important to explore new biological targets and find new therapeutic drugs according to new targets. “pRRophetic _ 0.5.tar” [48] packet contains 138 anticancer drugs and 727 cell lines. We use this package to look for targeted therapeutic agents that target DDX60 in pancreatic cancer. The lower the IC50, the more sensitive the drug.

### Cell culture and plasmid construction and western blotting

The cell line used in this study was pancreatic cancer cell line panc02 (Pancreatic cancer tissues from mice), which was gifted by School of Medicine, Xiamen University. The cell culture conditions were 37℃, 5% CO2. The medium was RPMI-1640 with 10% fetal bovine serum. Supplemented with 100 units/ml penicillin and 100 μg/ml streptomycin. The cells were cultured and passaged using a 10 cm disk, and the cell culture density was maintained at 70%. The plasmid was constructed using lentivirus as a vector. The sequence of the plasmid is DDX60-1: F-AGTATCCTGAAAGTGTAATAT, R-ATATTACACTTTCAGGATACT. DDX60-2: F-TTAGCAAAGGACCGCAATTTG, R-CAAATTGCCCTCCTTTGCTAA. DDX60-3: ATCTGACATCCTTCGTCTTTA, R-TAAAGACGAAGGATGTCAGAT. Lentivirus packaging was performed using 293 T, and the packaged virus infected the panc02 cell line. The infection time was 24 h. The virus solution was replaced with serum DMEM and continued to be cultured for 48 h. During each medium change, puro was added for drug screening. The cells were tested to confirm that DDX60 was knocked down in pancreatic cancer cells. The cells in the 10 cm disc were collected in a 1.5 ml centrifuge tube. Proteins are then extracted from the cells. After centrifugation, the protein was denatured by a metal bath at 99 °C. Protein was stored at-20 °C. The Western Blotting experiment was carried out to transfer the protein to the PVDF membrane. DDX60 antibody was purchased from abcam company, the antibody model is ab139807. The antibody was diluted at a ratio of 1:1000 and subsequently used for experiments.

### RNA-seq and bioinformatic and DAVID、STRING and Cytoscape

In order to further explore the biological function of DDX60 in pancreatic cancer, transcriptome sequencing was performed on the studied cell line, and the Panc02 cell line was selected in this study. According to the requirements of sequencing, we prepared 6 discs of cells, among which 3 discs of cells were no-load control group and 3 discs of cells with DDX60 gene knocked down were experimental group. The cells were incubated with viral fluid for 8 h, then replaced with a culture medium with serum for 48 h, during which the cell status was frequently observed. At 48 h, puro was used for drug screening, and when cell density reached about 80%, well-cultured cell lines were collected in a centrifuge tube loaded with trizol and handed over to sequencing companies. DAVID [49] database is a free database for biological function analysis. The advantage of this database is open source, large data processing capacity, and multiple species analysis can be carried out. We uploaded the differential genes to the DAVID database, species selected mice, and analyzed the differential genes by BP, CC, MF and KEGG. The *p*value < 0.05 is considered as statistically significant cutoff criterion. We further set the threshold as *P* < 0.05, |LOG2FC|≥ 2, and upload the genes that meet the above requirements to the STRING [50] database. The setting standard is as follows: We hide disconnected nodes in the network. Minimum required interaction score: 0.900. We download the analysis results in TSV format. Upload the TSV file to cytoscape software, We choose the MCODE plug‐in to analyze the entire network and set the parameters: Node score cutoff: 0.2, K‐core = 6, Finally, We end up with three MCODEs. The MCODE gene is the hub gene. We further analyzed MCODE genes to explore the biological processes involved in these genes.

### MTT and lactate dehydrogenase release experiment

The cells selected in this study were Panc02. Firstly, the growth state of the cells was observed. The cells in logarithmic growth phase were digested with trypsin, and then the cells were washed with PBS, centrifuged, and seeded in 96-well plates according to 1 × 104 per well, and each well kept 100 ul liquid. The edge holes of 96-well plates were filled with serum-free medium. Three holes were set in each group. Cells were cultured to 0. 24. 48. 72. 96 h, and 10ul MTT was added. The 96-well plate was placed on the biological safety cabinet between the cells and gently shaken, so that the MTT liquid was evenly placed in the compound hole of the 96-well plate. Then the cells were placed in a cell incubator for 4 h, and the absorbance at 490OD was measured by a microplate reader. The effect of DDX60 on cell proliferation was calculated according to the formula. All aseptic procedures in this experiment were performed in the biosafety cabinet. After the cells of the shvector group and shDDX60-1, shDDX60-2, shDDX60-3 were cultured for a period of time, the supernatant in the cell culture dish was collected in the centrifuge tube for lactate dehydrogenase analysis. Kit brand is Japanese Tongren company. Item number: CK12. According to the requirements of the kit, the absorption value was measured at 490 nm. Which reflects cell damage. LDH was detected by cell culture for 24 h.

### Colony formation experiment, EDU and immunohistochemistry

In this experiment, 6-well plates were selected, and 500 cells were added to each well in the experimental group and the control group. The cells were cultured in DMEM medium with 10% serum for 14 days, and the medium was changed every 3 days, and the cell surface was gently washed with PBS. After staining with trypan blue for 15 min, the camera was used for photo recording. Like the above experiment, we also selected 6-well plates for the experiment. Each well was inoculated with 500 cells. Follow the kit instructions. Finally, photographs were taken using a fluorescence microscope. Human Protein Altas (HPA) Database is an open and free database that stores a variety of tumor immunohistochemical databases. Both RNA and protein levels of target gene in normal tissue and tumor tissue were detected. Staining intensity was used to assess DDX60 expression level in pancreatic cancer tissues. We accessed DDX60 data from the HPA database (pancreatic cancer), which contained pathological slides from 3 normal subjects and 10 pancreatic cancer patients.

### Statistic analysis

In this study, Rstudio [51] and Graphpad Prism8 (GraphPad Software, CA., USA) software were used to analyze the microarray datasets using independent sample T test or paired sample T test. R 4.1.1 version was used in this artical, uses the "limma" package to obtain DEGs for microarray data. Perl language was used to process the original data from TCGA-PAAD. *P* < 0.05 was considered statistically significant.

## Results

### DEGs

**Fig. 1:**
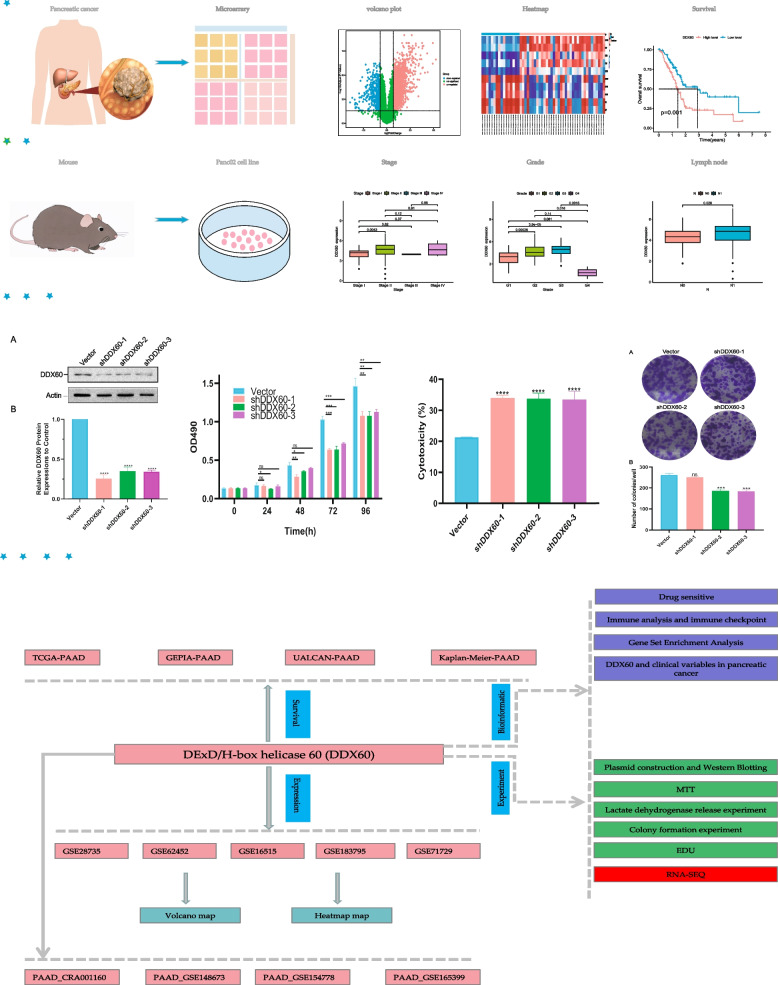
The pipeline diagram of this study manuscript

**Fig. 2:**
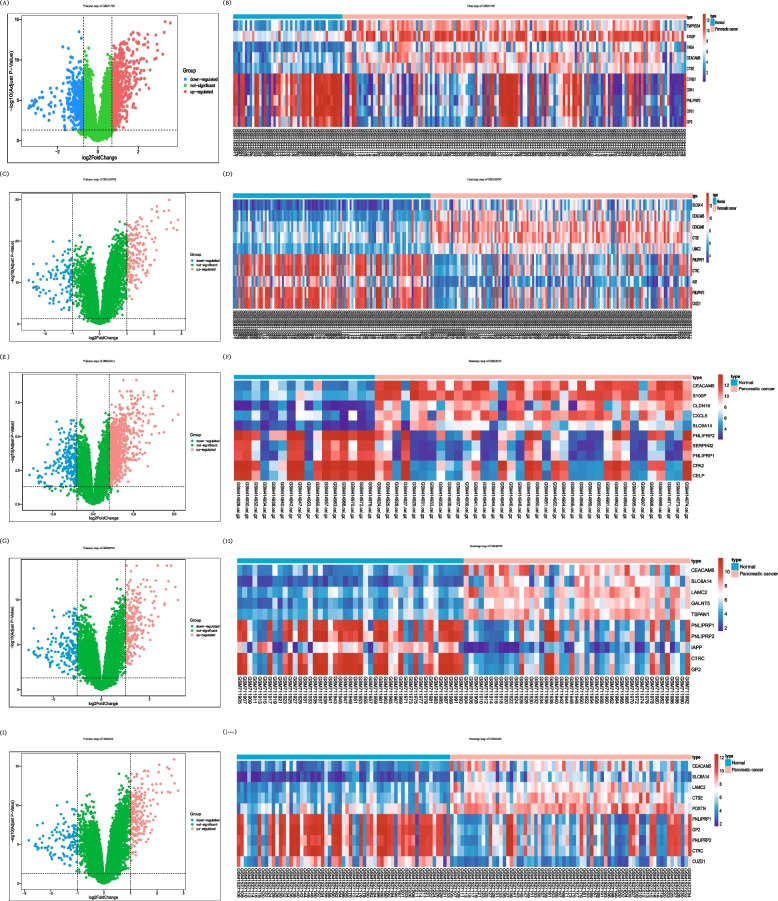
Differentially gene expression analysis in GSE71729, GSE183795, GSE16515, GSE28735 and GSE62452. **A** Volcano plot of the pancreatic ductal adenocarcinoma differentially expressed genes (DEGs) in GSE71729, between pancreatic ductal adenocarcinoma patients and normal subjects. Volcano plot of DEGs. Red means up-regulated DEGs; Blue means down-regulated DEGs; Green means no significance. **B** Heatmap of the pancreatic ductal adenocarcinoma differentially expressed genes (DEGs) in GSE71729, between pancreatic ductal adenocarcinoma patients and normal subjects. Heatmap of DEGs. Red means up-regulated DEGs; Blue means down-regulated DEGs; White means no significance. **C** Volcano plot of the pancreatic tumor differentially expressed genes (DEGs) in GSE183795, between pancreatic ductal adenocarcinoma patients and normal subjects. Volcano plot of DEGs. Red means up-regulated DEGs; Blue means down-regulated DEGs; Green means no significance. **D** Heatmap of the pancreatic tumor differentially expressed genes (DEGs) in GSE183795, between pancreatic ductal adenocarcinoma patients and normal subjects. Heatmap of DEGs. Red means up-regulated DEGs; Blue means down-regulated DEGs; White means no significance. **E** Volcano plot of the pancreatic ductal adenocarcinoma differentially expressed genes (DEGs) in GSE16515, between pancreatic ductal adenocarcinoma patients and normal subjects. Volcano plot of DEGs. Red means up-regulated DEGs; Blue means down-regulated DEGs; Green means no significance. **F** Heatmap of the pancreatic ductal adenocarcinoma differentially expressed genes (DEGs) in GSE16515, between pancreatic ductal adenocarcinoma patients and normal subjects. Heatmap of DEGs. Red means up-regulated DEGs; Blue means down-regulated DEGs; White means no significance. **G** Volcano plot of the pancreatic ductal adenocarcinoma differentially expressed genes (DEGs) in GSE28735, between pancreatic ductal adenocarcinoma patients and normal subjects. Volcano plot of DEGs. Red means up-regulated DEGs; Blue means down-regulated DEGs; Green means no significance **H** Heatmap of the pancreatic ductal adenocarcinoma differentially expressed genes (DEGs) in GSE28735, between pancreatic ductal adenocarcinoma patients and normal subjects. Heatmap of DEGs. Red means up-regulated DEGs; Blue means down-regulated DEGs; White means no significance. **I** Volcano plot of the pancreatic tumors differentially expressed genes (DEGs) in GSE62452, between pancreatic tumors patients and normal subjects. Volcano plot of DEGs. Red means up-regulated DEGs; Blue means down-regulated DEGs; Green means no significance. **J** Heatmap of the pancreatic tumors differentially expressed genes (DEGs) in GSE62452, between pancreatic tumors patients and normal subjects. Heatmap of DEGs. Red means up-regulated DEGs; Blue means down-regulated DEGs; White means no significance

Flow chart of this study in Fig. 1. A total of 191 participants from GSE71729 participated in this study. Using R language and calling the “limma” package, a total of 975 DEGs were identified, including 596 up-regulated genes and 379 down-regulated genes. Volcano and heat maps for data visualization (Fig. 2A-B). A total of 439 participants from GSE183795 participated in this study. Using R language and calling the “limma” package, a total of 647 DEGs were identified, including 439 up-regulated genes and 208 down-regulated genes. Volcano and heat maps for data visualization (Fig. 2C-D). A total of 52 participants from GSE16515 participated in this study. Using R language and calling the “limma” package, a total of 2257 DEGs were identified, including 1750 up-regulated genes and 507 down-regulated genes. Volcano and heat maps for data visualization (Fig. 2E-F). A total of 90 participants from GSE28735 participated in this study. Using R language and calling the “limma” package, a total of 1032 DEGs were identified, including 612 up-regulated genes and 420 down-regulated genes. Volcano and heat maps for data visualization (Fig. 2G-H). A total of 130 participants from GSE62452 participated in this study. Using R language and calling the “limma” package, a total of 774 DEGs were identified, including 519 up-regulated genes and 255 down-regulated genes. Volcano and heat maps for data visualization (Fig. 2I-J). The differential gene lists of GSE71729, GSE183795, GSE16515, GSE28735, and GSE62452 datasets are in the Table 1.

### GO terms and KEGG pathways analysis of DEGs significantly enriched in PAAD

**Fig. 3:**
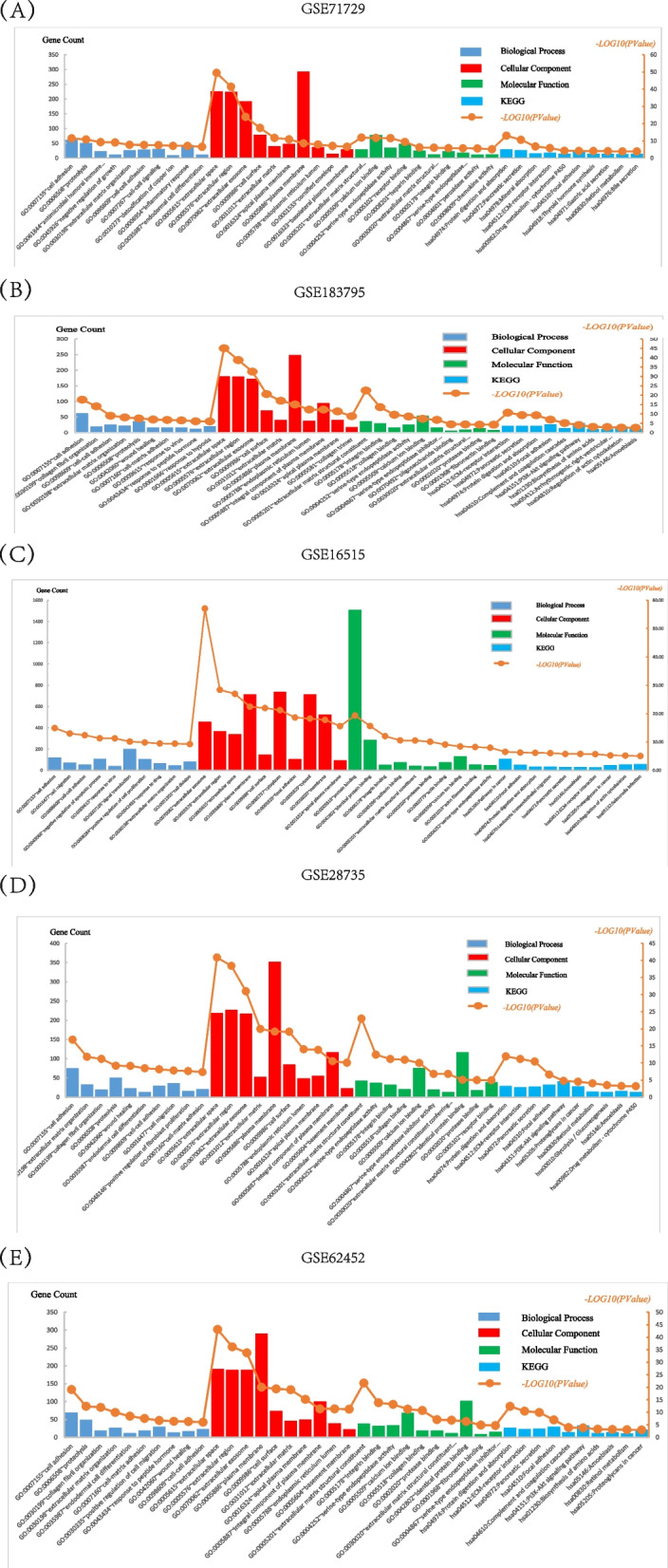
GO terms and KEGG pathways analysis of DEGs significantly enriched in PAAD. **A** The top ten BP, CC, MF and pathways in GSE71729. **B** The top ten BP, CC, MF and pathways in GSE183795. **C** The top ten BP, CC, MF and pathways in GSE16515. **D** The top ten BP, CC, MF and pathways in GSE28735. **E** The top ten BP, CC, MF and pathways in GSE62452

As shown in Fig. 3A and Supplementary Table 1, in the GSE71729 dataset. The DEGs were mainly enriched in GO:0007155 ~ cell adhesion, GO:0006508 ~ proteolysis, GO:0061844 ~ antimicrobial humoral immune response mediated by antimicrobial peptide, GO:0005615 ~ extracellular space, GO:0005576 ~ extracellular region, GO:0070062 ~ extracellular exosome, GO:0005201 ~ extracellular matrix structural constituent, GO:0005509 ~ calcium ion binding, GO:0004252 ~ serine-type endopeptidase activity, hsa04974:Protein digestion and absorption, hsa04972:Pancreatic secretion, and hsa04978: Mineral absorption. As shown in Fig. 3B and Supplementary Table 2, in the GSE183795 dataset. The DEGs were mainly enriched in GO:0007155 ~ cell adhesion, GO:0030199 ~ collagen fibril organization, GO:0098609 ~ cell–cell adhesion, GO:0005615 ~ extracellular space, GO:0005576 ~ extracellular region, GO:0070062 ~ extracellular exosome, GO:0005201 ~ extracellular matrix structural constituent,GO:0005178 ~ integrin binding, GO:0005518 ~ collagen binding, hsa04512:ECM-receptor interaction, hsa04972:Pancreatic secretion, and hsa04974:Protein digestion and absorption. As shown in Fig. 3C and Supplementary Table 3, in the GSE16515 dataset. The DEGs were mainly enriched in GO:0007155 ~ cell adhesion, GO:0016477 ~ cell migration, GO:0098609 ~ cell–cell adhesion, GO:0070062 ~ extracellular exosome, GO:0005576 ~ extracellular region, GO:0005615 ~ extracellular space, GO:0005515 ~ protein binding, GO:0042802 ~ identical protein binding, GO:0005178 ~ integrin binding, hsa05200:Pathways in cancer, hsa04510:Focal adhesion, and hsa04974:Protein digestion and absorption. As shown in Fig. 3D and Supplementary Table 4, in the GSE28735 dataset. The DEGs were mainly enriched in GO:0007155 ~ cell adhesion, GO:0030198 ~ extracellular matrix organization, GO:0030199 ~ collagen fibril organization, GO:0005615 ~ extracellular space, GO:0005576 ~ extracellular region, GO:0070062 ~ extracellular exosome, GO:0005201 ~ extracellular matrix structural constituent, GO:0004252 ~ serine-type endopeptidase activity, GO:0005178 ~ integrin binding, hsa04974:Protein digestion and absorption, hsa04512:ECM-receptor interaction, and hsa04972:Pancreatic secretion. As shown in Fig. 3E and Supplementary Table 5, in the GSE62452 dataset. The DEGs were mainly enriched in GO:0007155 ~ cell adhesion, GO:0006508 ~ proteolysis, GO:0030199 ~ collagen fibril organization, GO:0005615 ~ extracellular space, GO:0005576 ~ extracellular region, GO:0070062 ~ extracellular exosome, GO:0005201 ~ extracellular matrix structural constituent, GO:0005178 ~ integrin binding, GO:0004252 ~ serine-type endopeptidase activity, hsa04974:Protein digestion and absorption, hsa04512:ECM-receptor interaction, and hsa04972:Pancreatic secretion.

### The expression level of DDX60 in pancreatic cancer

**Fig. 4:**
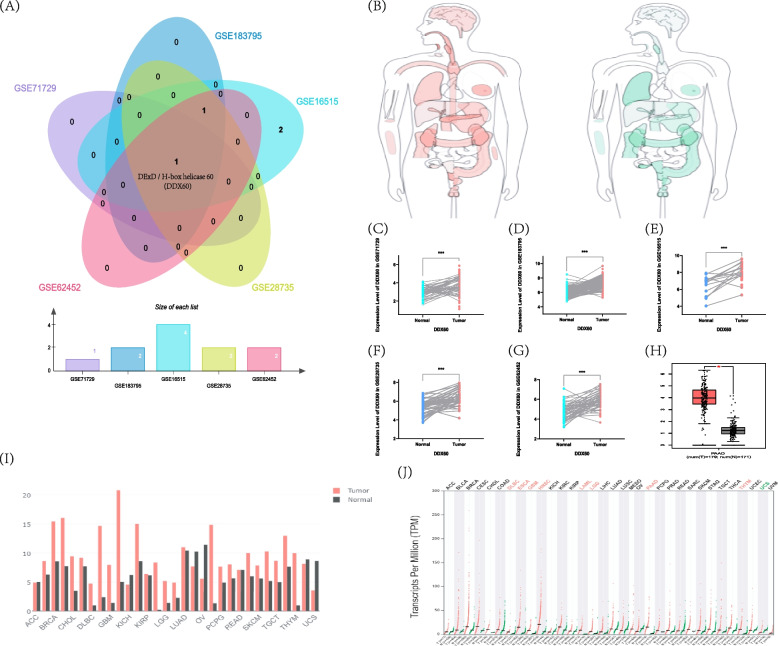
The expression level of DDX60 in pancreatic cancer. **A** Common genes of DExD/H-box RNA helicase in GSE71729, GSE183795, GSE16515, GSE28735 and GSE62452. **B** Expression analysis of DDX60 in each organ in GEPIA database (The expression value in pancreatic tissues was 14.85; The expression value in pancreas tissues was 1.34). **C** The expression level of DDX60 in GSE71729. **D** The expression level of DDX60 in GSE183795. **E** The expression level of DDX60 in GSE16515. **F** The expression level of DDX60 in GSE28735. (**G**). The expression level of DDX60 in GSE62452. **H** The expression level of DDX60 in GEPIA database. **I** Use a bar chart to show the expression level of DDX60 in GEPIA database. **J** Use a dot plot to show the expression level of DDX60 (TPM:Transcripts Per Million) in GEPIA database

We intersected the DExD/H-box RNA helicase genes in five datasets, and finally we obtained DDX60. The results are shown in the form of a Venn diagram **(**Fig. 4A**)**. Firstly, we explored the mRNA expression of DDX60 between pancreatic cancer groups and normal control groups in five GEO datasets respectively. From the results, we learned that the expression level of DDX60 in pancreatic cancer was significantly higher than that in normal tissues and was statistically significant. The GEPIA database again verified that DDX60 was highly expressed in pancreatic cancer (*P* < 0.05) (Fig. 4B-J). From the CPTAC database, we know that the protein level of DDX60 is significantly higher in pancreatic tumor tissues than in normal tissues. DDX60 may play a role of oncogene in pancreatic cancer (Supplementary Fig. 1).

### Survival Analysis of DDX60 in pancreatic cancer

**Fig. 5:**
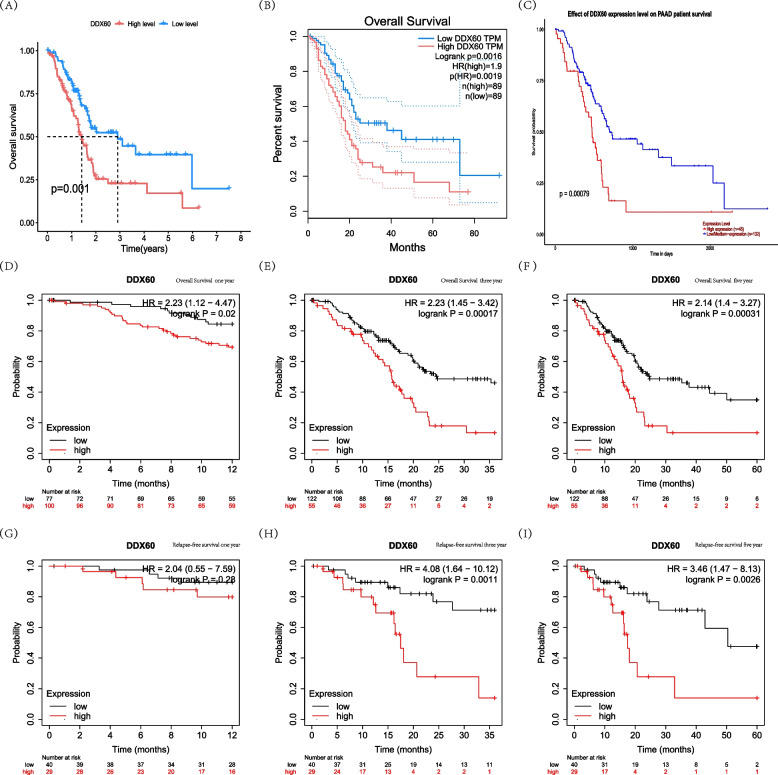
The survival analysis of DDX60 in pancreatic cancer. **A** Overall survival of DDX60 in TCGA-PAAD dataset. **B** Overall survival of DDX60 in GEPIA-PAAD dataset. **C** Overall survival of DDX60 in UALCAN-PAAD dataset. **D** Overall survival of DDX60 in the Kaplan Meier-PAAD database with a cut-off value of 1 year. **E** Overall survival of DDX60 in the Kaplan Meier-PAAD database with a cut-off value of 3 year. **F** Overall survival of DDX60 in the Kaplan Meier-PAAD database with a cut-off value of 5 year. **G** Overall survival of DDX60 in the Kaplan Meier-PAAD database with a cut-off value of 5 year. **H** Relapse-free survival of DDX60 in the Kaplan Meier-PAAD database with a cut-off value of 1 year. **I** Relapse-free survival of DDX60 in the Kaplan Meier-PAAD database with a cut-off value of 3 year. **J** Relapse-free survival of DDX60 in the Kaplan Meier-PAAD database with a cut-off value of 3 year. **K** Relapse-free survival of DDX60 in the Kaplan Meier-PAAD database with a cut-off value of 5 year

Firstly, we explored the survival value of DDX60 between high-risk group andlow-risk group in the TCGA-PAAD database. We found that the survival rate of DDX60 in high-risk group was lower, and the results were statistically significant (*P* = 0.001) (Fig. 5A). We used the GEPIA dataset to analyze DDX60 survival differences in pancreatic cancer. The survival rate was lower in the group with high DDX60 expression (Logrank *p* = 0.0016, HR = 1.9, *P* = 0.0019), and the patients in the high score group and the low risk group was 89 respectively (Fig. 5B). We continued to analyze the prognostic value of DDX60 using the UALCAN database. Pancreatic cancer patients in UALCAN database included 45 patients in the medium–high risk group and 132 patients in the low-risk group. We found that patients with high DDX60 expression had poor prognostic value (*P* = 0.00079) (Fig. 5C). We further used the Kaplan Meier database to analyze DDX60 survival differences in pancreatic tumors. From the database, we know that DDX60 has a statistically significant effect on the overall survival of pancreatic cancer patients at 1 [HR = 2.23 (1.12–4.47; logrank *P* = 0.02)] (Fig. 5D), 3 [HR = 2.23 (1.45–3.42; logrank P = 0.00017)] **(**Fig. 5E), and 5 [HR = 2.14 (1.4–3.27; logrank P = 0.00031)] (Fig. 5F) years. There was also an effect on 3 [HR = 4.08 (1.64–10.12; logrank *P* = 0.0011)] (Fig. 5H) and 5 [HR = 3.46 (1.47–8.13; logrank *P* = 0.0026)] (F ig. 5I) year RFS of pancreatic cancer patients, but no effect on 1 [HR = 2.04 (0.55–7.59; logrank *P* = 0.28)] year RFS (Fig. 5G). These results indicate that DDX60 can affect the prognosis of pancreatic cancer patients. The higher the expression of DDX60, the worse the prognosis of patients.

### The relationship between DDX60 and clinical variables in pancreatic cancer

**Fig. 6:**
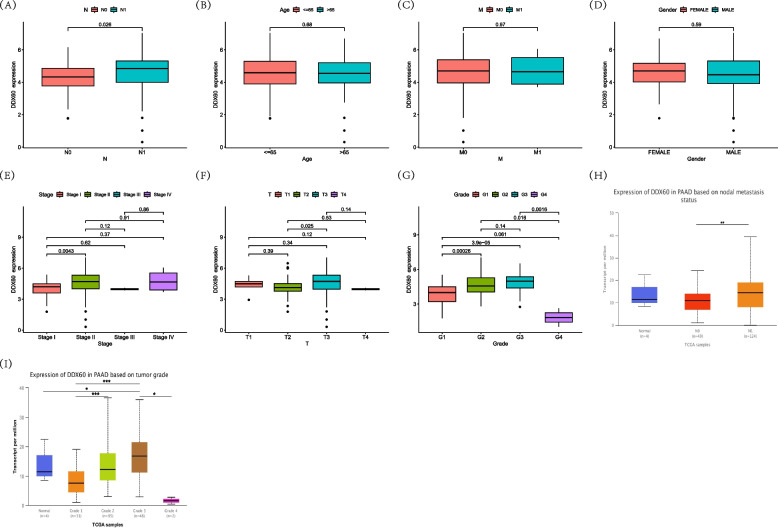
Relationship between DDX60 expression level and clinical variables in the TCGA-PAAD database. **A** The expression level of DDX60 in PAAD patients was related to N metastasis, which was statistically significant. The expression level of DDX60 in N1 was higher than that in N0 (*P* = 0.026). **B** The expression level of DDX60 in PAAD patients was not related to age groups (age ≤ 65, vs age > 65), and there was no statistical significance. There was no significant difference in the expression level of DDX60 between age ≤ 65 and age > 65 (*P* = 0.68). **C** The expression level of DDX60 in PAAD patients was not related to M, and there was no statistical significance. There was no significant difference in the expression level of DDX60 between M0 and M1 (*P* = 0.97). **D** The expression level of DDX60 in PAAD patients was not related to gender, and there was no statistical significance. There was no significant difference in the expression level of DDX60 between female and male (*P* = 0.59). **E** The expression level of DDX60 in PAAD patients was related to stage, which was statistically significant. The expression level of DDX60 in stageII was higher than that in stageI (*P* = 0.0043). The comparison of other stages was not statistically significant. **F** The expression level of DDX60 in PAAD patients was related to T, which was statistically significant. The expression level of DDX60 in T3 was higher than that in T2 (*P* = 0.025). The comparison of other T was not statistically significant. **G** The expression level of DDX60 in PAAD patients was related to grade, which was statistically significant. The expression level of DDX60 in grade2 was higher than that in grade1 (*P* = 0.00026), DDX60 level in grade3 was higher than that in grade1 (*P* = 3.9e-05). Since there are only 2 patients in grade 4, we discard this subgroup. **H** The expression level of DDX60 in PAAD patients was related to N, which was statistically significant. The expression level of DDX60 in N1 (124 patients) was higher than that in N0 (49 patients) (*P* = 7.216100E-03). The database used in the above study is UALCAN. **I** The expression level of DDX60 in PAAD patients was related to grade, which was statistically significant. The expression level of DDX60 in grade2 was higher than that in grade1 (*P* = 1.68942999999588E-05), DDX60 level in grade3 was higher than that in grade1 (*P* = 1.63574999999527E-05). The database used in the above study is UALCAN. Grade4 only has 2 patients, we discard this stage

Clinical variables are of great value in the clinical treatment of tumor patients, especially for patients with distant metastasis. They are of great guiding value in clinical treatment and are crucial to the formulation of surgical protocols. We learned from the results that DDX60 was more highly expressed in the group with lymph node metastasis than in the group without lymph node metastasis, which was statistically significant. Therefore, DDX60 can be used as an indicator for the detection of lymph node metastasis in clinical pancreatic cancer. The expression level of DDX60 was not associated with clinical variables such as age, distant metastasis, sex, and T stage. However, DDX60 is associated with the clinical stage and pathological grade of pancreatic cancer patients, which is clinically important. According to the results, DDX60 expression level in stage 2 was significantly higher than that in stage1. The expression level of DDX60 in grade1, grade2, and grade3 shows an increasing trend. Considering that there were only 2 patients in grade 4, we discarded this reference index and speculated that the expression level of DDX60 was positively correlated with patient grade. The higher the DDX60 expression level, the worse the pathological grade, the worse the prognosis and the worse the treatment response (Fig. 6).

#### GSEA

**Fig. 7:**
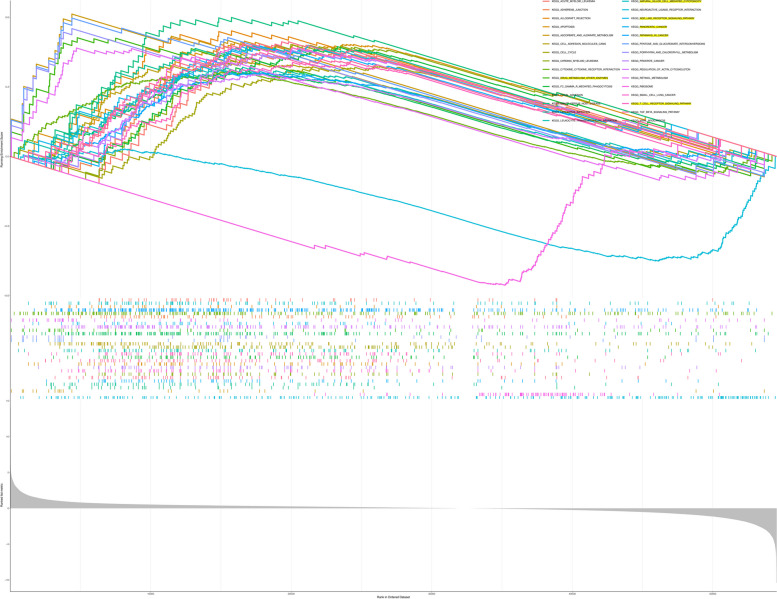
GSEA analysis of DDX60 in TCGA-PAAD. As can be seen from the figure, DDX60 is involved in tumor biological processes, mainly involved in biological pathways related to pancreatic cancer. And it's associated with immune-related pathways. Such as: T cell receptor signaling pathway, natural killer cell mediated sytotoxicity and drug-metabolism other enzymes

GSEA is used to search for important enrichment pathways in high or low expression types of DDX60. As shown in the Fig. 7, we know that DDX60 is mainly involved in tumor-related pathways, immune-related pathways and drug metabolism-related pathways. Such as KEGG_DRUG_METABOLISM_OTHER_ENZYMES,NOD_LIKE_RECEPTOR_SIGNALING_PATHWAY, PANCREATIC_CANCER, PATHWAY IN CANCER,T_CELL_RECEPTOR_SIGNALING_PATHWAY,DRUG_METABOLISM_CYTOCHROME_P450 (Table 2). In summary, we reveal the biological pathway of DDX60 involvement in pancreatic cancer.

**Table 2:** GSEA analysis of DDX60 in TCGA-PAAD

Description	*p*value	p.adjust	NES
KEGG_RIBOSOME	0.001438849	0.044433827	-1.802937216
KEGG_NEUROACTIVE_LIGAND_RECEPTOR_INTERACTION	0.00117096	0.044433827	-1.679813841
KEGG_LONG_TERM_POTENTIATION	0.047761194	0.155852317	-1.384110207
KEGG_CHEMOKINE_SIGNALING_PATHWAY	0.025380711	0.10978633	1.294487924
KEGG_NEUROTROPHIN_SIGNALING_PATHWAY	0.012145749	0.068956995	1.350475913
KEGG_TOLL_LIKE_RECEPTOR_SIGNALING_PATHWAY	0.038869258	0.145882353	1.330502805
KEGG_ENDOCYTOSIS	0.014925373	0.075030254	1.416757511
KEGG_REGULATION_OF_ACTIN_CYTOSKELETON	0.005747126	0.044433827	1.467734547
KEGG_JAK_STAT_SIGNALING_PATHWAY	0.014018692	0.072429907	1.415621838
KEGG_PATHWAYS_IN_CANCER	0.007042254	0.048513302	1.572828039
KEGG_LYSOSOME	0.012244898	0.068956995	1.406805715
KEGG_FOCAL_ADHESION	0.005434783	0.044433827	1.493787366
KEGG_TIGHT_JUNCTION	0.012605042	0.068956995	1.435677746
KEGG_CYTOKINE_CYTOKINE_RECEPTOR_INTERACTION	0.00621118	0.044433827	1.569156082
KEGG_NATURAL_KILLER_CELL_MEDIATED_CYTOTOXICITY	0.008403361	0.052173913	1.478293587
KEGG_LEUKOCYTE_TRANSENDOTHELIAL_MIGRATION	0.003831418	0.044433827	1.488463275
KEGG_CELL_CYCLE	0.004081633	0.044433827	1.5148155
KEGG_RENAL_CELL_CARCINOMA	0.03313253	0.130722892	1.387252188
KEGG_ECM_RECEPTOR_INTERACTION	0.01618123	0.079202862	1.468459505
KEGG_UBIQUITIN_MEDIATED_PROTEOLYSIS	0.008695652	0.052173913	1.566017079
KEGG_B_CELL_RECEPTOR_SIGNALING_PATHWAY	0.019417476	0.088089036	1.446423319
KEGG_T_CELL_RECEPTOR_SIGNALING_PATHWAY	0.003759398	0.044433827	1.566679395
KEGG_GLYCOLYSIS_GLUCONEOGENESIS	0.042424242	0.147749338	1.402970915
KEGG_DRUG_METABOLISM_CYTOCHROME_P450	0.027355623	0.115639679	1.463301414
KEGG_CELL_ADHESION_MOLECULES_CAMS	0.004132231	0.044433827	1.619831911
KEGG_METABOLISM_OF_XENOBIOTICS_BY_CYTOCHROME_P450	0.021604938	0.095679012	1.499849211
KEGG_ENDOMETRIAL_CANCER	0.042857143	0.147749338	1.443045739
KEGG_NON_SMALL_CELL_LUNG_CANCER	0.031428571	0.129470979	1.485879794
KEGG_TGF_BETA_SIGNALING_PATHWAY	0.003257329	0.044433827	1.680016169
KEGG_NUCLEOTIDE_EXCISION_REPAIR	0.04	0.145882353	1.465877884

### Drug sensitive

**Fig. 8:**
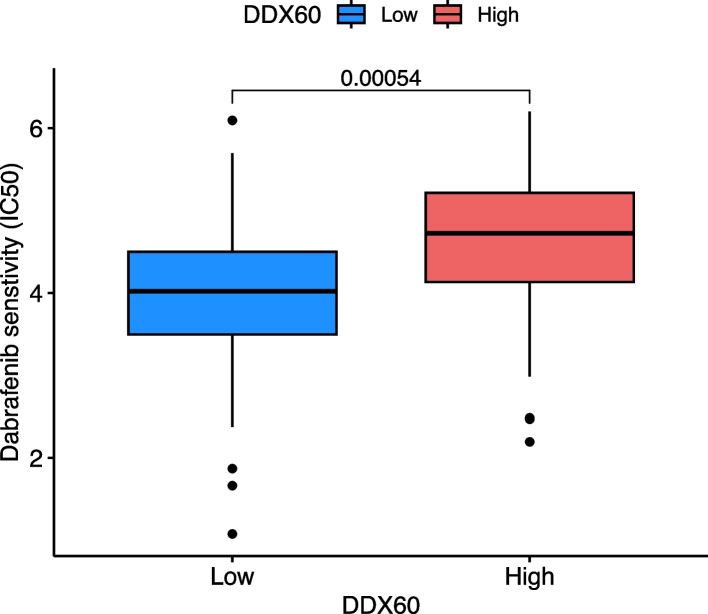
The relationship between DDX60 and 132 chemotherapeutic drugs contained in “pRRophetic” package. There was IC50 sensitivity difference between 8 chemotherapy drugs and DDX60 high risk group and low risk group. PAAD patients in the DDX60 low-risk group were more sensitive to Dabrafenib (*P* = 0.00054)

In the course of clinical treatment of cancer patients, most of them are accompanied by drug therapy, and one of the important indicators affecting the effect of drug therapy is IC50. The purpose of our study is to find drugs with smaller IC50 as far as possible, so as to improve the sensitivity of clinical cancer patients to therapeutic drugs and improve the prognosis of patients. By calling “pRRophetic _ 0.5.tar” packet with R language, we get a total of 11 drugs with pFilter = 0.001, respectively CEP-701(*P* = 0.00043), Dabrafenib (*P* = 0.00054), TW37 (*P* = 0.00054), Cyclopamine (*P* = 6e-06), JW-7–52-1 (*P* = 0.00025), Paclitaxel (*P* = 1.4e-07), Rapamycin (*P* = 1.4e-05), Sunitinib (*P* = 4.4e-06), TAE684 (P = 5.5e-05), WZ-1–84 (*P* = 0.00038), and Z-LLNle-CHO (*P* = 8.4e-06). The IC50 of CEP-701, Dabrafenib and TW3 in the high-risk group was higher than that in the low-risk group, indicating that the DDX60 high-expression group was relatively insensitive to these three chemotherapy drugs than the low expression group. Comparatively speaking, the IC50 value of Cyclopamine, JW-7–52-1, Paclitaxel, Rapamycin, Sunitinib, TAE684, WZ-1–84, and Z-LLNle-CHO in the high-risk group was lower than that in the low-risk group, indicating that high-risk pancreatic cancer patients were relatively sensitive to these eight drugs, and clinical trials could be attempted. This study provides the direction for clinical drug treatment (Fig. 8). Other drugs that are not important are included in the supplement file Supplement Fig. 9. For experimental verification, we selected Dabrafenib, which was purchased from the flagship store of MCE under the catalog number HY-14660. DMSO was used for dissolution, and the final concentration selected was 1 nM. The LDH experimental results were in Supplement Fig. 10. From the results, we learned that pancreatic cancer cell lines in the DDX60 dilution group were more sensitive to Dabrafenib.

**Fig. 9:**
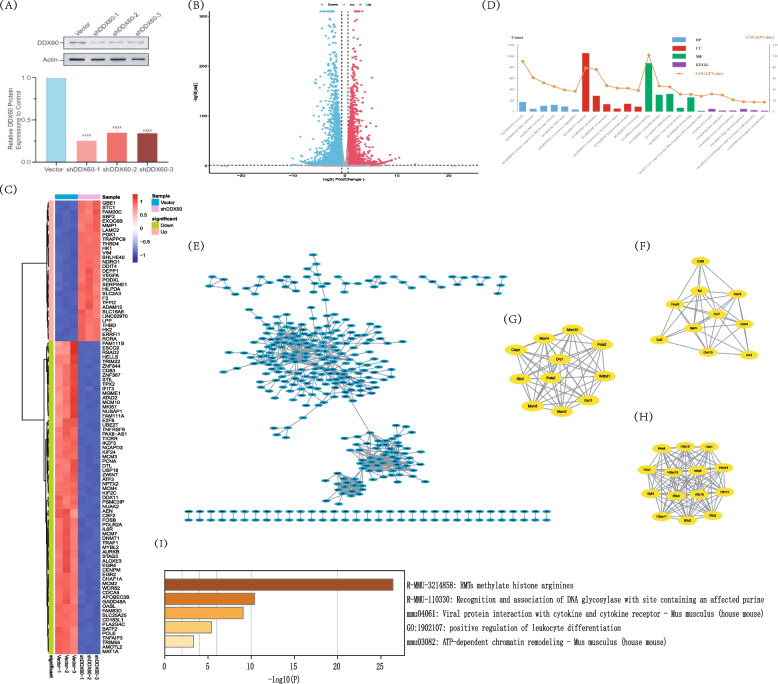
Plasmid Construction、Western Blotting and RNA-seq analysis. (**A**) Western Blotting experiment was used to detect the effect of plasmid obtained by molecular cloning. The shDDX60-1, shDDX60-2 and shDDX60-3 were successfully constructed, and DDX60 could be knocked down at protein levels in three plasmid. (**A**)Western Blotting images of plasmid effect detection were developed using a developer. (**B**) Quantitative ananlysis of western blot. (**B**) Volcano plot of DEGs. (**C**) Heatmap of DEGs. (**D**) Gene ontology (GO) and Kyoto Encyclopedia of Genes and Genomes (KEGG)analysis of DEGs. BP: Biological Process, CC: cellular component, MF: molecular function, KEGG: Kyoto Encyclopedia of Genes and Genomes. (**E**) Functional and pathway analysis based on cytoscape (PPI network). (**F**) MCODE1. (**G**) MCODE2. (**H**) MCODE3. (**I**) KEGG enrichment of MCODE genes

**Fig. 10:**
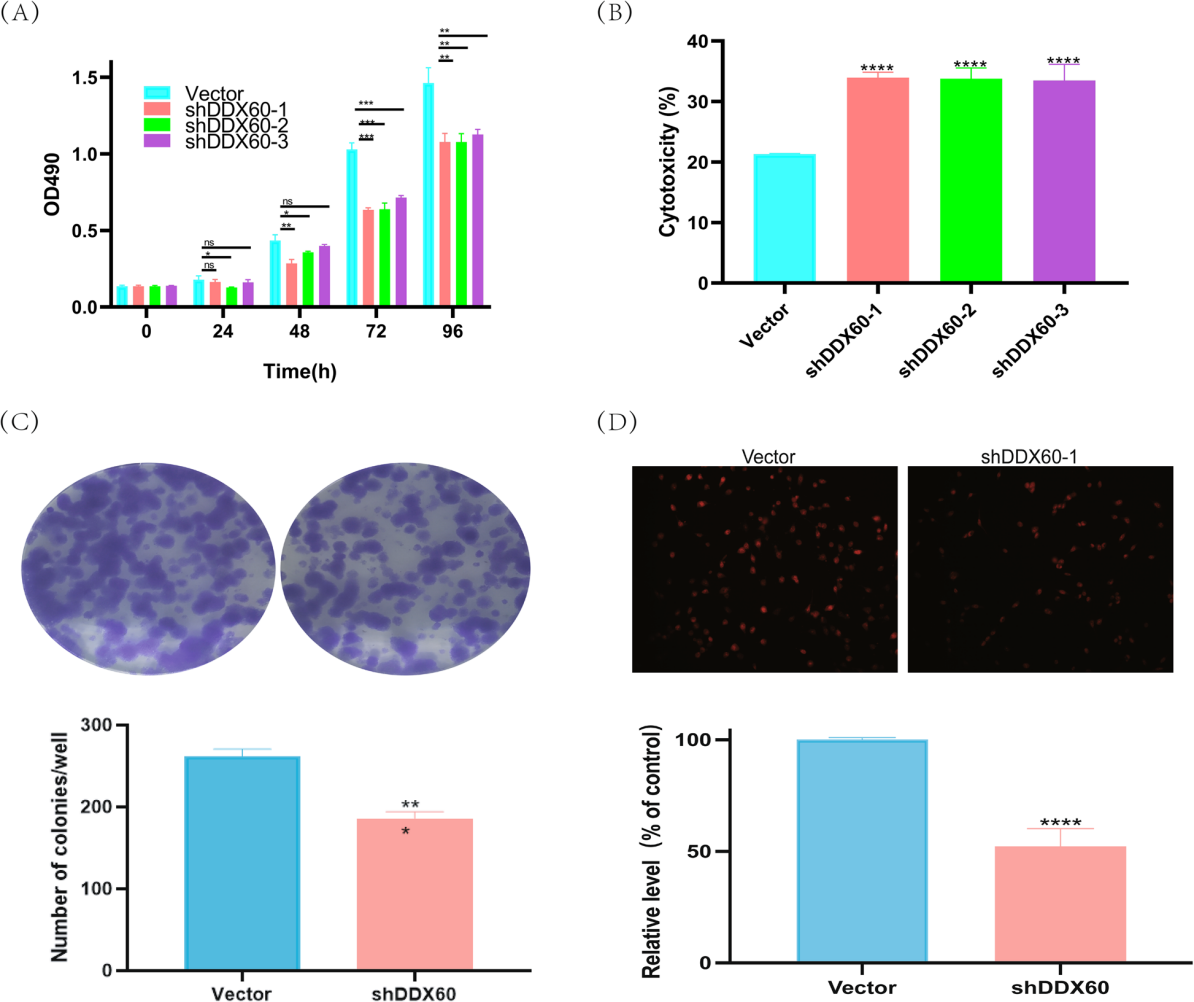
Molecular Biology Experiment. (**A**) When DDX60 in Panc02 cells of pancreatic cancer is knocked down, cell proliferation is decreased. MTT assay for cell viability. Data are expressed as mean ± SD, *n* = 3,* *P* < 0.05,** *P* < 0.01, * * * * *P* < 0.0001, compared with the control group. (**B**) LDH release experiment. Knock down DDX60 in Panc02 cells of pancreatic cancer, the LDH release ability of cells was enhanced, which had statistical significance. Data are expressed as mean ± SD, *n* = 3,*P < 0.05,** *P* < 0.01, * * * * *P* < 0.0001, compared with the control group. (**C**) The effect of DDX60 on cell proliferation was evaluated by colony formation assay. The cell proliferation ability of DDX60 knockdown group was lower than that of vector group (*P* < 0.001). Quantitative ananlysis of cell proliferation. (**D**) The effect of DDX60 on cell proliferation was evaluated by EDU. From the results, we know that the proliferation ability of tumor cells with low DDX60 is significantly decreased

### Plasmid construction、western blotting and RNA-seq analysis

After plasmid construction, we tested the effect of plasmid construction, WB experiment was selected for verification, the virus after plasmid packaging was infected with pancreatic cancer cell line Panc02, and then protein was extracted for electrophoresis. Finally, after PVDF membrane transfer, DDX60 antibody was used for incubation for 24 h, and then secondary antibody was used for incubation. After membrane washing, the instrument was developed. We observed that the plasmid was constructed successfully. Compared with the Vector group, DDX60 protein in shDDX60-1, shDDX60-2 and shDDX60-3 groups was knocked down (*P* < 0.05) (Fig. 9A). The original data analysis was completed by the sequencing company, and the initial analysis was completed by Xiamen Emer Biotechnology Co., Ltd. Comparing the DDX60 knockdown group with the vector group, we found a total of 2934 differential expressed genes (DEGs), of which the number of up-regulated genes was 1383 and the number of down-regulated genes was 1551. Threshold was set at P < 0.05, |LOG2 fold change|≥ 1. DEGs were displayed in the form of volcano plots and heat maps, respectively (Fig. 9B-C). Compared vector group with shDDX60 group, the DEGs were mainly enriched in GO:0007049 ~ cell cycle, GO:0006260 ~ DNA replication, GO:0051301 ~ cell division, GO:0006974 ~ cellular response to DNA damage stimulus, GO:0006281 ~ DNA repair, and GO:0007059 ~ chromosome segregation in BP. DEGs were mainly enriched in GO:0005737 ~ cytoplasm, GO:0005856 ~ cytoskeleton, GO:0005694 ~ chromosome, GO:0000775 ~ chromosome, centromeric region, GO:0098978 ~ glutamatergic synapse, and GO:0005874 ~ microtubule in CC. DEGs were mainly enriched in GO:0005515 ~ protein binding, GO:0000166 ~ nucleotide binding, GO:0003677 ~ DNA binding, GO:0008017 ~ microtubule binding, GO:0005524 ~ ATP binding, and GO:0017116 ~ single-stranded DNA-dependent ATP-dependent DNA helicase activity in MF. DEGs were mainly enriched in mmu04110:Cell cycle, mmu03030:DNA replication, mmu03460: Fanconi anemia pathway, mmu04015:Rap1 signaling pathway, mmu05412: Arrhythmogenic right ventricular cardiomyopathy, and mmu03440:Homologous recombination in KEGG. (Fig. 9D) (Table 3). In order to study the key genes of DDX60 in the biological role of cell cycle in pancreatic cancer, we imported 990 genes into the online database. The PPI network information is: number of nodes:881, number of edges: 781, average node degree: 1.77, avg. local clustering coefficient:0.3, expected number of edges: 345, and PPI enrichment p-value: < 1.0e-16. After further entering the TSV file into cytoscape, set K = 6, and get three MCODE modules. The gene in the module1 are *H3C1, H2AC11, H2AC13, H4C8, DPF3, H3C4, H4C1, H3C10, H4C9, H3C3, H4C14, H3C15, H3C2,* and *H3C14.* The gene in the module2 are *EXO1, POLA2, CLSPN, RFC3, MCM4, MCM2, MCM8, ORC1, POLE2, WDHD1,* and *MCM10.* The gene in the module3 are *ITGAM, CXCR3, CCR1, CXCl10, CCR7, CXCR4, CSF2, TNF, FOXP3,* and *CD69* (Fig. 9E-H)*.* As a potential target downstream of DDX60, MCODE gene was found to be mainly enriched in RMTs methylate histone arginines, Recognition and association of DNA glycosylase with site containing an affected purine, Viral protein interaction with cytokine and cytokine receptor-Mus musculus (house mouse), positive regulation of leukocyte differentiation, and ATP-dependent chromatin remodeling—Mus musculus (house mouse) (Fig. 9I).

**Table 3:** The top six GO and KEGG enrichment analysis of DEGs

Description	Term	Count	*P*Value	FDR
GOTERM_BP_DIRECT	GO:0007049 ~ cell cycle	174	4.81E-28	3.07E-24
GOTERM_BP_DIRECT	GO:0006260 ~ DNA replication	53	3.52E-19	1.12E-15
GOTERM_BP_DIRECT	GO:0051301 ~ cell division	106	2.23E-16	4.74E-13
GOTERM_BP_DIRECT	GO:0006974 ~ cellular response to DNA damage stimulus	123	2.20E-14	3.51E-11
GOTERM_BP_DIRECT	GO:0006281 ~ DNA repair	95	1.90E-12	2.43E-09
GOTERM_BP_DIRECT	GO:0007059 ~ chromosome segregation	41	9.30E-12	9.90E-09
GOTERM_CC_DIRECT	GO:0005737 ~ cytoplasm	1055	1.87E-24	1.63E-21
GOTERM_CC_DIRECT	GO:0005856 ~ cytoskeleton	286	1.25E-23	5.42E-21
GOTERM_CC_DIRECT	GO:0005694 ~ chromosome	134	6.83E-15	1.98E-12
GOTERM_CC_DIRECT	GO:0000775 ~ chromosome, centromeric region	54	1.63E-13	3.38E-11
GOTERM_CC_DIRECT	GO:0098978 ~ glutamatergic synapse	140	1.94E-13	3.38E-11
GOTERM_CC_DIRECT	GO:0005874 ~ microtubule	89	3.17E-12	4.60E-10
GOTERM_MF_DIRECT	GO:0005515 ~ protein binding	874	1.90E-31	3.34E-28
GOTERM_MF_DIRECT	GO:0000166 ~ nucleotide binding	305	9.86E-15	8.67E-12
GOTERM_MF_DIRECT	GO:0003677 ~ DNA binding	322	3.73E-14	2.18E-11
GOTERM_MF_DIRECT	GO:0008017 ~ microtubule binding	69	3.25E-10	1.38E-07
GOTERM_MF_DIRECT	GO:0005524 ~ ATP binding	258	3.93E-10	1.38E-07
GOTERM_MF_DIRECT	GO:0017116 ~ single-stranded DNA-dependent ATP-dependent DNA helicase activity	15	4.16E-09	1.13E-06
KEGG_PATHWAY	mmu04110:Cell cycle	48	2.27E-10	6.90E-08
KEGG_PATHWAY	mmu03030:DNA replication	20	9.51E-10	1.45E-07
KEGG_PATHWAY	mmu03460:Fanconi anemia pathway	21	3.71E-07	3.76E-05
KEGG_PATHWAY	mmu04015:Rap1 signaling pathway	49	4.17E-06	2.99E-04
KEGG_PATHWAY	mmu05412:Arrhythmogenic right ventricular cardiomyopathy	26	5.50E-06	2.99E-04
KEGG_PATHWAY	mmu03440:Homologous recombination	17	5.91E-06	2.99E-04

### Molecular biology experiment

In order to investigate whether DDX60 knockdown can inhibit the proliferation of pancreatic cancer cells, we knockout DDX60 in Panc02 cells. By MTT assay, we found that the proliferative ability of pancreatic cancer cells with DDX60 knockdown was inhibited (Fig. 10A). When cells are damaged, lactate dehydrogenase will be released into the medium, and then through chemical reactions, the experimenter can measure the light absorption value at 490OD, and the cell activity can be obtained through calculation. From the results, we learned that LDH release ability was enhanced in the group of cells with DDX60 knocked down, indicating serious cell damage (Fig. 10B). Clonogenesis experiments can visually reflect the proliferation capacity of cells. From the experimental results, we can know that the proliferation ability of cells in DDX60 group was significantly inhibited by knocking down DDX60. These results indicated that DDX60 had a statistically significant effect on the proliferation of pancreatic cancer cells (*P* < 0.05) (Fig. 10C). EDU experiment was used to observe the effect of DDX60 on the proliferative ability of pancreatic cancer cells. The results showed that the proliferation ability of pancreatic cancer cells decreased when DDX60 level was knocked down (*P* < 0.05) (Fig. 10D). Consistent with the above analysis results, immunohistochemical results showed that DDX60 was highly expressed in pancreatic cancer tissues compared to pancreatic tissues (Supplement Fig. 11).

## Discussion

Pancreatic cancer, a highly malignant tumor originating in the abdomen, poses a severe threat to global health. It imposes significant health and economic strains on both individual patients and nations. While early detection allows for surgical removal of the tumor, subsequent treatments like radiotherapy, chemotherapy, and molecular targeting can prolong patient survival. However, most patients are diagnosed at an advanced stage, precluding surgical options and rendering traditional tumor treatments less effective. There is an urgent need for innovative diagnostics to enhance early detection. To address this, we utilized comprehensive and publicly accessible genomic databases, TCGA and GEO, to analyze and sequence data using R and Perl languages. Our analysis led to the identification of a novel biomarker, DDX60, which serves as a diagnostic and prognostic tool for pancreatic cancer. Additionally, we predicted candidate drugs targeting this gene, potentially offering new therapeutic avenues for patients.

Our research introduces several novel findings regarding DDX60 in pancreatic cancer. We have identified DDX60 as a potential diagnostic and therapeutic biomarker, a discovery unprecedented in this context. Our analysis showed that elevated DDX60 expression correlates with reduced relapse-free survival (RFS) and overall survival (OS), and it is particularly upregulated in more advanced tumor stages (N1) and in regions with higher malignancy. Experimental evidence from our study supports the role of DDX60 in promoting the proliferation of pancreatic cancer cells, indicating its tumor-promoting potential in pancreatic adenocarcinoma (PAAD). Additionally, we have linked high DDX60 expression to key immune-related pathways, such as B_CELL_RECEPTOR_SIGNALING_.

PATHWAY and KEGG_T_CELL_RECEPTOR_SIGNALING_PATHWAY, which are implicated in cancer progression and the pancreatic cancer microenvironment. Furthermore, we have established that DDX60 is overexpressed at both the transcriptome and protein levels in pancreatic cancer, marking it as a prognostic indicator of poor outcomes. Its expression is also positively associated with risk factors within the pancreatic cancer tumor microenvironment (TME), suggesting a role in immune privilege and the infiltration of immune cells like T and B cells [12].

In conclusion, our work positions DDX60 as a significant biomarker for the diagnosis and prognosis of pancreatic cancer, with implications for developing targeted therapies.

Conventional parameters such as the expression level of target genein RNA and protein level, T, M, N, stage and grade are helpful to predict the prognosis of pancreatic cancer patients to a certain extent in clinical practice. From our study, we know that DDX60 is highly expressed in pancreatic cancer at both RNA and protein levels (*P* < 0.05), indicating that DDX60 may be a proto-oncogene driving the formation of pancreatic cancer. It was further found that the prognosis of patients with high risk of DDX60 was poor, indicating that DDX60, as a proto-oncogene, is associated with poor prognosis of patients with pancreatic cancer. Lymph node status is one of the most important predictors of survival of pancreatic ductal adenocarcinoma, and the prognosis of pancreatic cancer patients with lymph node metastasis is generally poor [52]. Our study showed that DDX60 expression in N1 group was higher than that in N0 group (*P* = 0.026), indicating that DDX60 may drive tumor lymph node metastasis in pancreatic cancer, thus leading to poor prognosis in patients with pancreatic cancer. Grade3 has a higher DDX60 than Grade1 patients (*P* = 3.9e-05). Grade2 has higher DDX60 expression than Grade1 patients (*P* = 0.00026). These results indicate that pancreatic cancer patients in DDX60 high-risk group have higher pathological grade. StageII showed higher DDX60 expression than StageI patients (*P* = 0.0043). These results suggest that DDX60 may promote clinical staging of pancreatic cancer in the initial stage of development.

By analyzing single cell sequencing data, we found that DDX60 was positively correlated with malignant tumor cells. Therefore, it further confirms our preliminary research results that DDX60 can play the role of proto-oncogene. In addition, DDX60 was positively correlated with the immune score in the tumor immune microenvironment. Moreover, DDX60 is negatively correlated with T cell regulatory pathway (Tregs), and we predict that DDX60 may inhibit immune-related passage and the anticancer effect of the body. In addition, we have identified several unreported therapeutic agents related to DDX60 targets. Such as: Cyclopamine, JW-7–52-1, Paclitaxel, Rapamycin, Sunitinib, TAE684, WZ-1–84, and Z-LLNle-CHO. Our study provides promising clinical directions, but it needs to be confirmed in clinical trials.

We acknowledge that our study has some limitations: (A) We have no conditions to collect tumor samples from clinical pancreatic cancer patients for protein-level verification, but only through various public databases for verification, and no conditions for sequencing. (B) Due to the limitation of funding and scientific research conditions, we only conducted molecular biology experiments in the direction of proliferation, but did not carry out molecular biology experiments in the direction of apoptosis and immunity. (C) We have no direct evidence to support a relationship between targeting DDX60 and immune checkpoints, or whether immunotherapy targeting DDX60 is feasible in pancreatic cancer. (D) If possible, we can cooperate with researchers from different cancer centers in different countries to verify pancreatic cancer tumor tissues of different ethnic groups.

## Conclusion

Through an in-depth analysis of sequencing data from the TCGA and GEO databases, we have identified DDX60, a member of the DExD/H-box RNA helicase family, as a significant biomarker in pancreatic cancer. Our findings demonstrate a significant correlation between elevated DDX60 expression and a poor prognosis in patients with pancreatic cancer. GSEA analysis has revealed that higher DDX60 expression levels are associated with increased immune cell infiltration and enhanced activity of drug therapy response pathways. Furthermore, DDX60 has been linked to the immune infiltration and tumor immune microenvironment in patients, suggesting its involvement in biological processes related to tumor immune dynamics in pancreatic cancer. We have also conducted drug sensitivity assays targeting DDX60, leading to the discovery of therapeutic drugs that exhibit relative sensitivity to this gene target. Molecular biology experiments, including MTT, LDH, EDU, and clonal formation assays, have provided further evidence that the knockdown of DDX60 can effectively inhibit the proliferation of pancreatic cancer cells. These results underscore the potential of DDX60 as a therapeutic target and highlight its role in the pathogenesis of pancreatic cancer. 

## Supplementary Information

Supplementary Material 1: Supplement Figure1. DDX60 protein expression levels in pancreatic cancer tissues and normal pancreas tissues according to the CPTAC database.

Supplementary Material 2: Supplement Figure2. High expression DDX60 positive with malignant cells in PAAD_CRA001160.

Supplementary Material 3: Supplement Figure3. High expression DDX60 positive with malignant cells in PAAD_GSE148673.

Supplementary Material 4: Supplement Figure4. High expression DDX60 positive with malignant cells in PAAD_GSE154778.

Supplementary Material 5: Supplement Figure5. High expression DDX60 positive with malignant cells in PAAD_GSE165399.

Supplementary Material 6: Supplement Figure6. High expression of DDX60 promotes immune infiltration of pancreatic cancer. DDX60 was positively correlated with stromal scores, immune scores and estimate score in the tumor microenvironment.

Supplementary Material 7: Supplement Figure7. The relationship between DDX60 and 21 immune cells. A DDX60 positively correlated with Macrophages M1 (cor=0.31; *P*=0.0047). B DDX60 positively correlated with dendritic cell activated (cor=0.26; *P*=0.02). C DDX60 negatively correlated with B cells naive (cor=-0.29; *P*=0.0088). D DDX60 negatively correlated with T cells regulatory (Tregs) (cor=-0.26; *P*=0.022). E A lollipop chart of the relationship between DDX60 and 21 immune cells in pancreatic cancer.

Supplementary Material 8: Supplement Figure8. The relationship between DDX60 and immune checkpoints. DDX60 was positively correlated with immune checkpoint CD274，LGALS9, CD80, CD44, HAVCR2, CD86, TNFSF4, HHLA2, PDCD1LG2, NRP1, CD40, IDO1, TNFSF18, LAIR1, CD276, TNFSF15, TNFRSF9, TNFSF9, TNFRSF14, CD244, CD70, CTLA4, ICOS, and TIGIT (*P*＜0.05).

Supplementary Material 9: Supplement Figure9. The relationship between DDX60 and 132 chemotherapeutic drugs contained in “pRRophetic” package. There was IC50 sensitivity difference between 8 chemotherapy drugs and DDX60 high risk group and low risk group. (A)PAAD patients in the DDX60 high-risk group were more sensitive to WZ-1-84 (*P*=0.00038). (B)PAAD patients in the DDX60 low-risk group were more sensitive to TW37 (*P*=0.00054). (C)PAAD patients in the DDX60 high-risk group were more sensitive to TAE684 (*P*=5.5e-05). (D)PAAD patients in the DDX60 high-risk group were more sensitive to JW-7-52-1 (*P*=0.00025). (E)PAAD patients in the DDX60 high-risk group were more sensitive to Cyclopamine (*P*=6e-06). (F)PAAD patients in the DDX60 high-risk group were more sensitive to Sunitinib (*P*=4.4e-06). (G)PAAD patients in the DDX60 high-risk group were more sensitive to Rapamycin (*P*=1.4e-05). (H)PAAD patients in the DDX60 high-risk group were more sensitive to Paclitaxel (*P*=1.4e-07). (I)PAAD patients in the DDX60 high-risk group were more sensitive to Z-LLNle-CHO(*P*=8.4e-06). (J)PAAD patients in the DDX60 low-risk group were more sensitive to CEP-701(*P*=0.00043).

Supplementary Material 10: Supplement Figure10. LDH results confirmed that pancreatic cancer cell lines in the knockdown group were more sensitive to Dabrafenib.

Supplementary Material 11.

Supplementary Material 12.

Supplementary Material 13.

Supplementary Material 14.

Supplementary Material 15: Supplement Figure11. The IHC-based protein expression of DDX60 in PAAD tissues and normal pancreatic tissue. Normal subject information: Female, age 74, Patients id: 2162. HPA number: HPA046952. PAAD subject information: Female, age 73, Patient id: 3719. HPA number: HPA046952.

Supplementary Material 16: Supplement Table 1. The top ten GO and KEGG enrichment analysis of DEGs in GSE71729.

Supplementary Material 17: Supplement Table 2. The top ten GO and KEGG enrichment analysis of DEGs in GSE183795.

Supplementary Material 18: Supplement Table 3. The top ten GO and KEGG enrichment analysis of DEGs in GSE16515.

Supplementary Material 19: Supplement Table 4. The top ten GO and KEGG enrichment analysis of DEGs in GSE28735.

Supplementary Material 20: Supplement Table 5. The top ten GO and KEGG enrichment analysis of DEGs in GSE62452.

Supplementary Material 21: Supplement Table 6. Correlation analysis between DDX60 and 22 kinds of immune cells in pancreatic cancer (*P*＜0.05).

Supplementary Material 22: Supplement Table 7. Correlation analysis between DDX60 and 24 kinds of immune checkpoint in pancreatic cancer (*P*＜0.05).

Supplementary Material 23.

Supplementary Material 24.

## Data Availability

No datasets were generated or analysed during the current study.
