# Supplementary figures and images for: Deciphering the potential ability of DExD/H-box helicase 60 (DDX60) on the proliferation, diagnostic and prognostic biomarker in pancreatic cancer: a research based on silico, RNA-seq and molecular biology experiment
Source: Hereditas. 2025 Jan 22;162:6. doi: 10.1186/s41065-024-00361-9 (PMC11753068; doi:10.1186/s41065-024-00361-9)

**DDX60 Pancreatic Ductal Adenocarcinoma Protein Abundance**  
(Unpaired P-Value: < 0.0001)

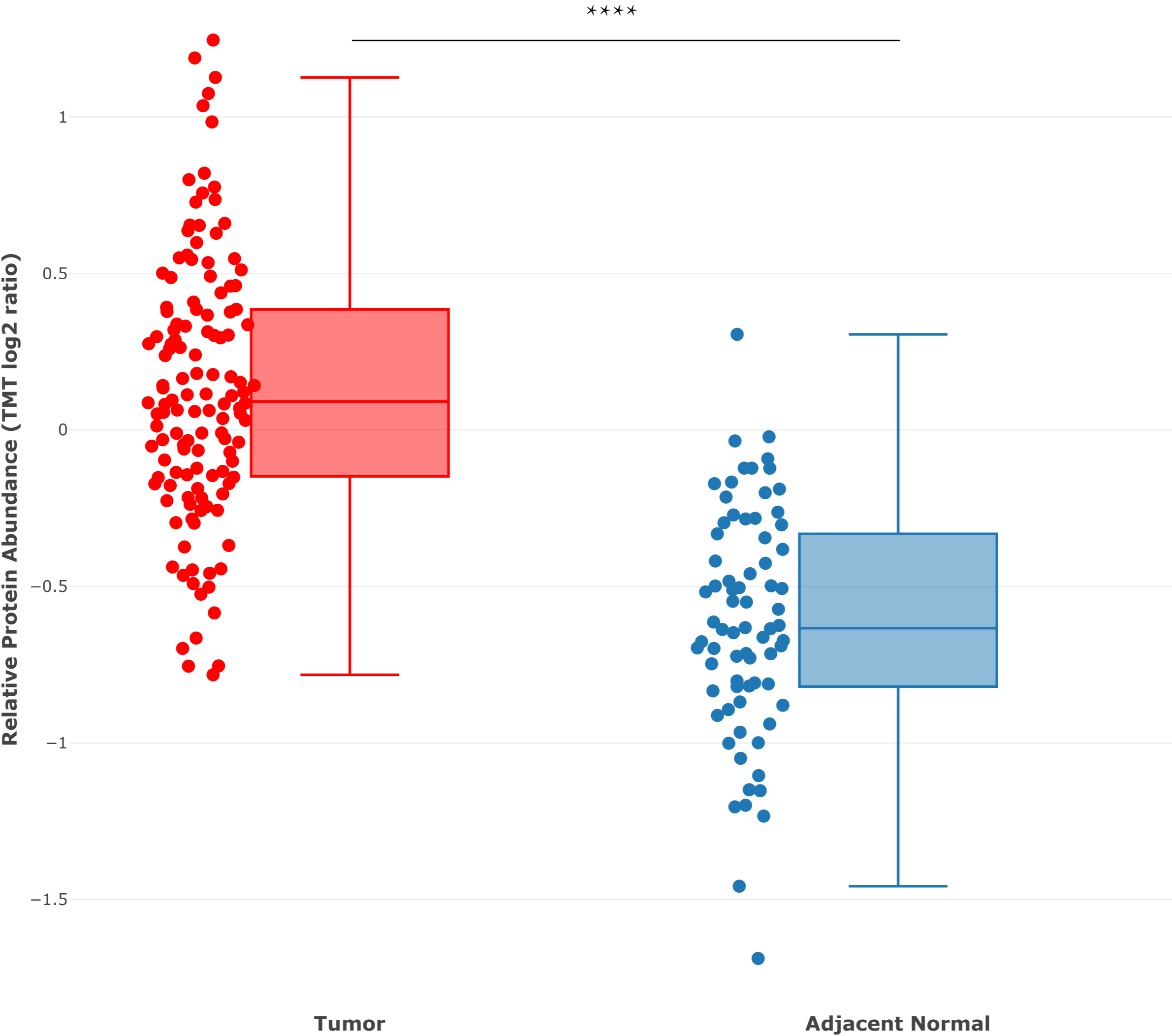

Supplement: Supplementary file 1 — Supplementary Material 1: Supplement Figure1. DDX60 protein expression levels in pancreatic cancer tissues and normal pancreas tissues according to the CPTAC database. [file 41065_2024_361_MOESM1_ESM.pdf]

(A)

PAAD\_CRA001160

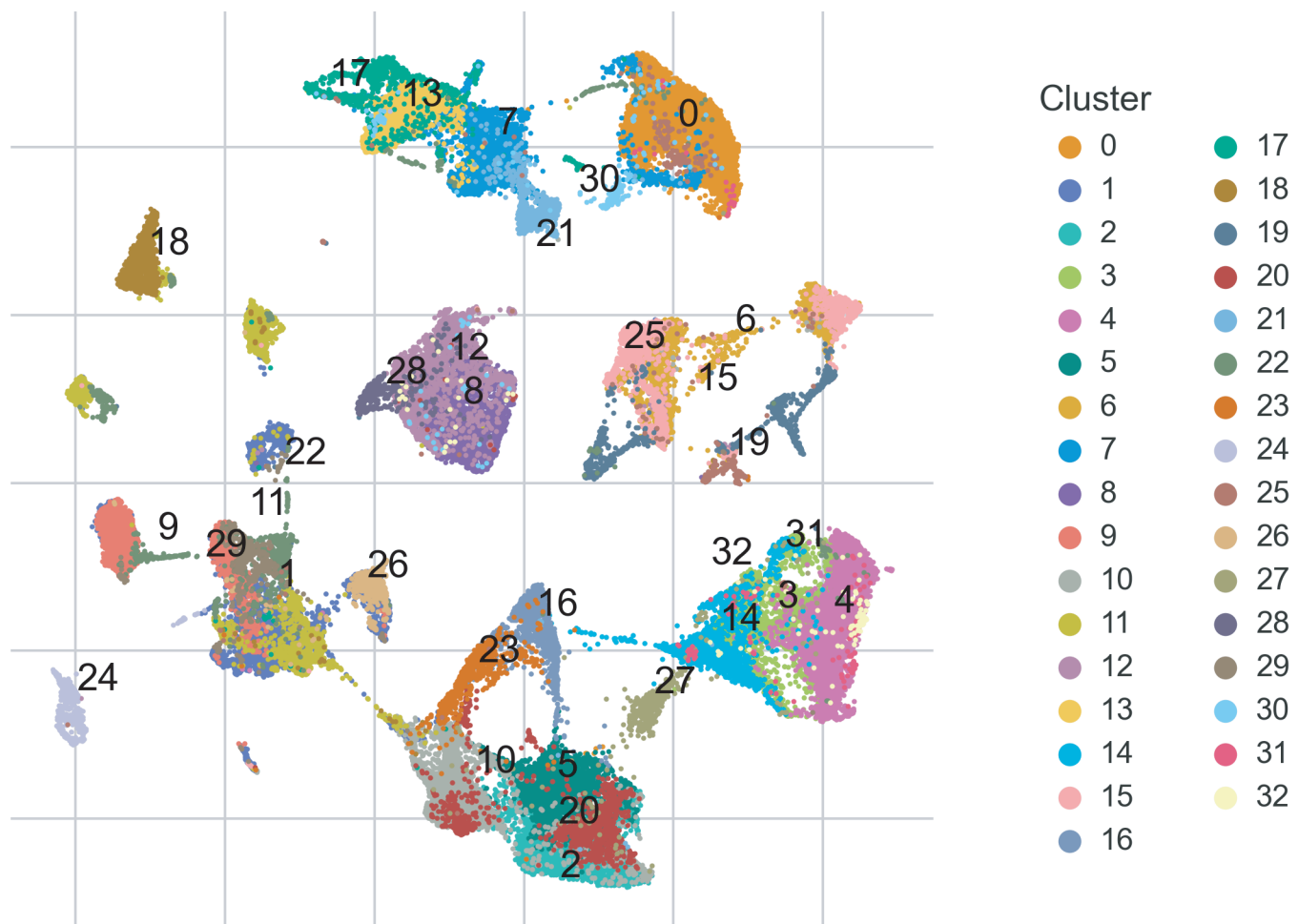

(B)

PAAD\_CRA001160

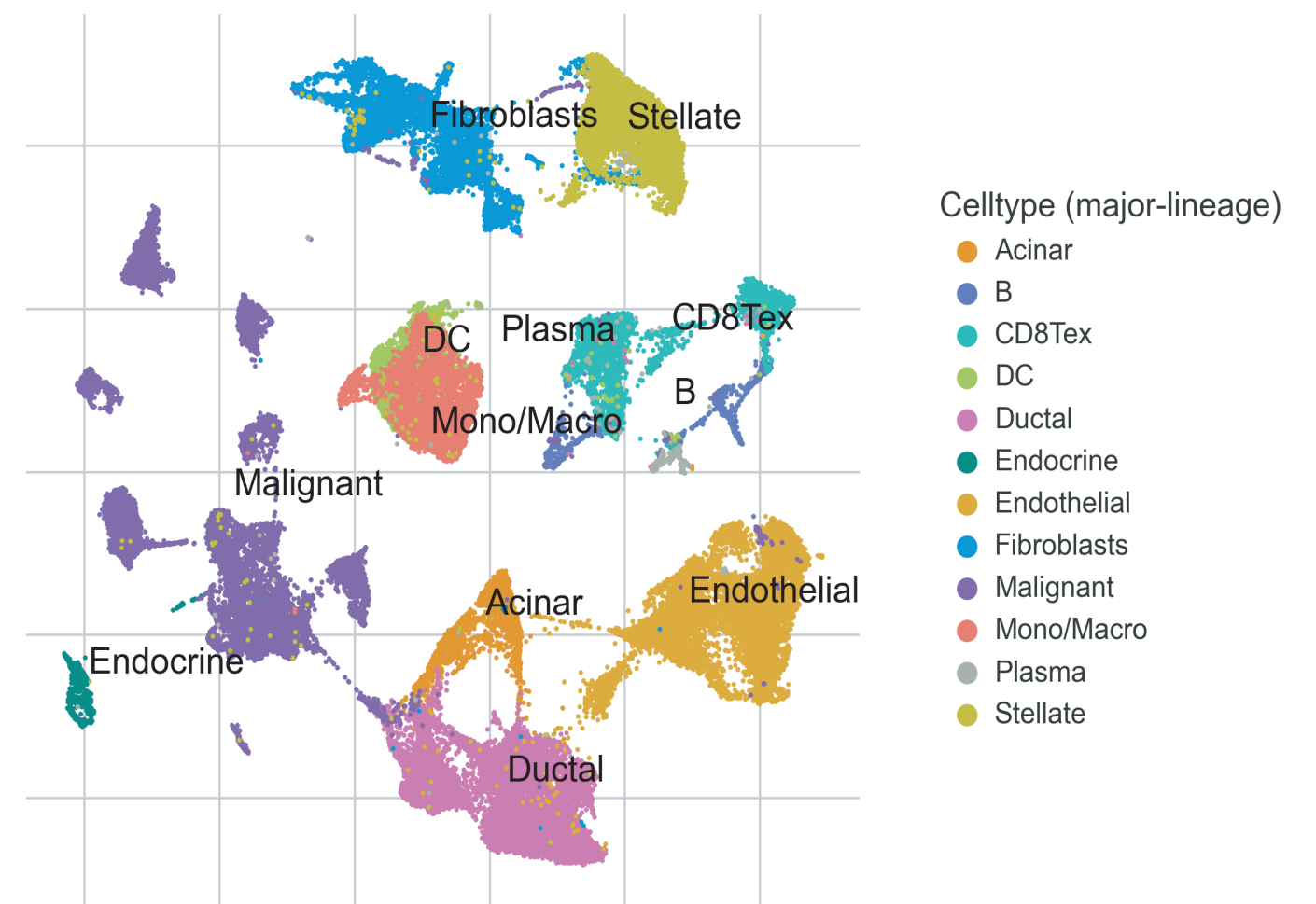

(C)

DDX60

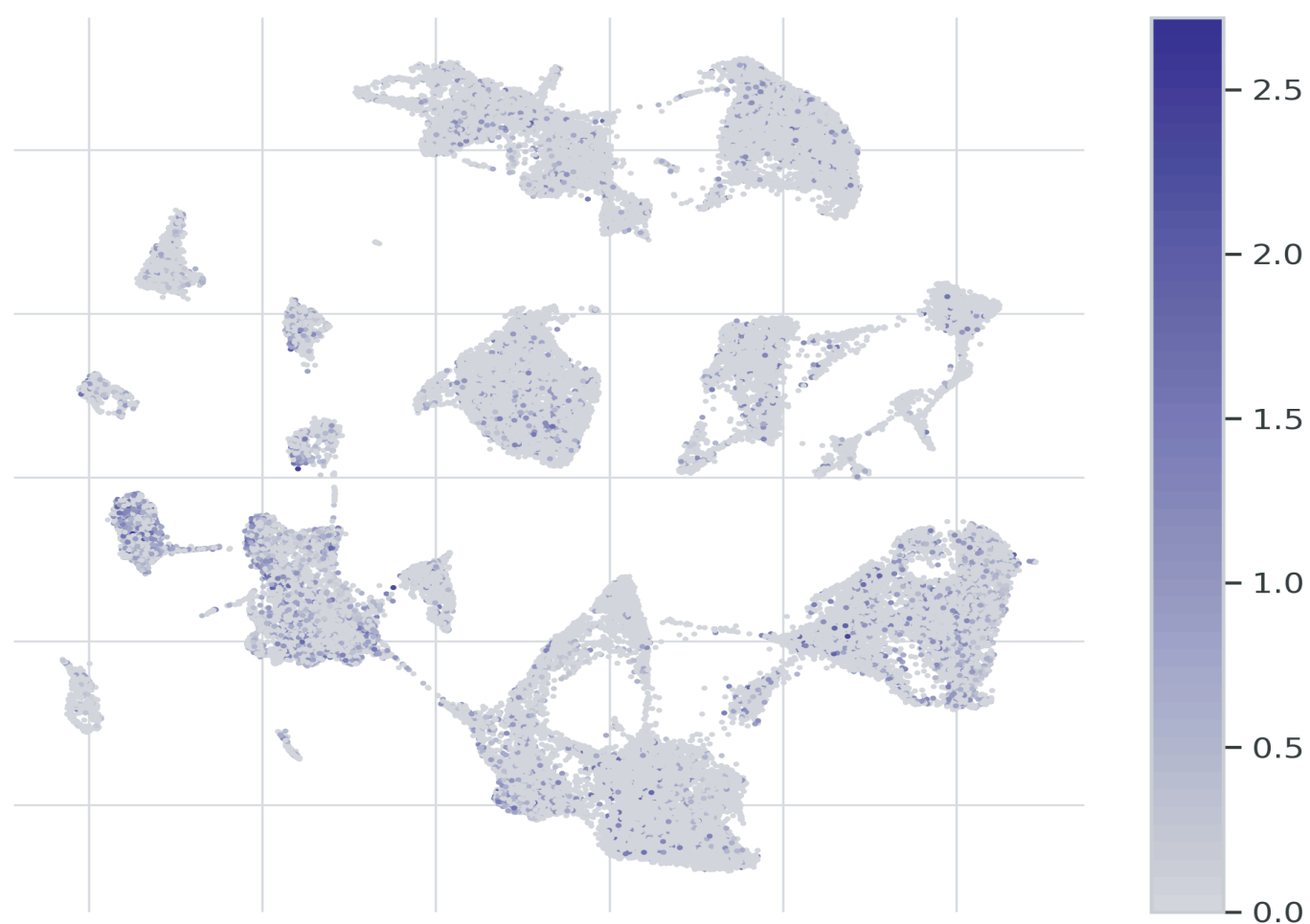

(D)

PAAD\_CRA001160

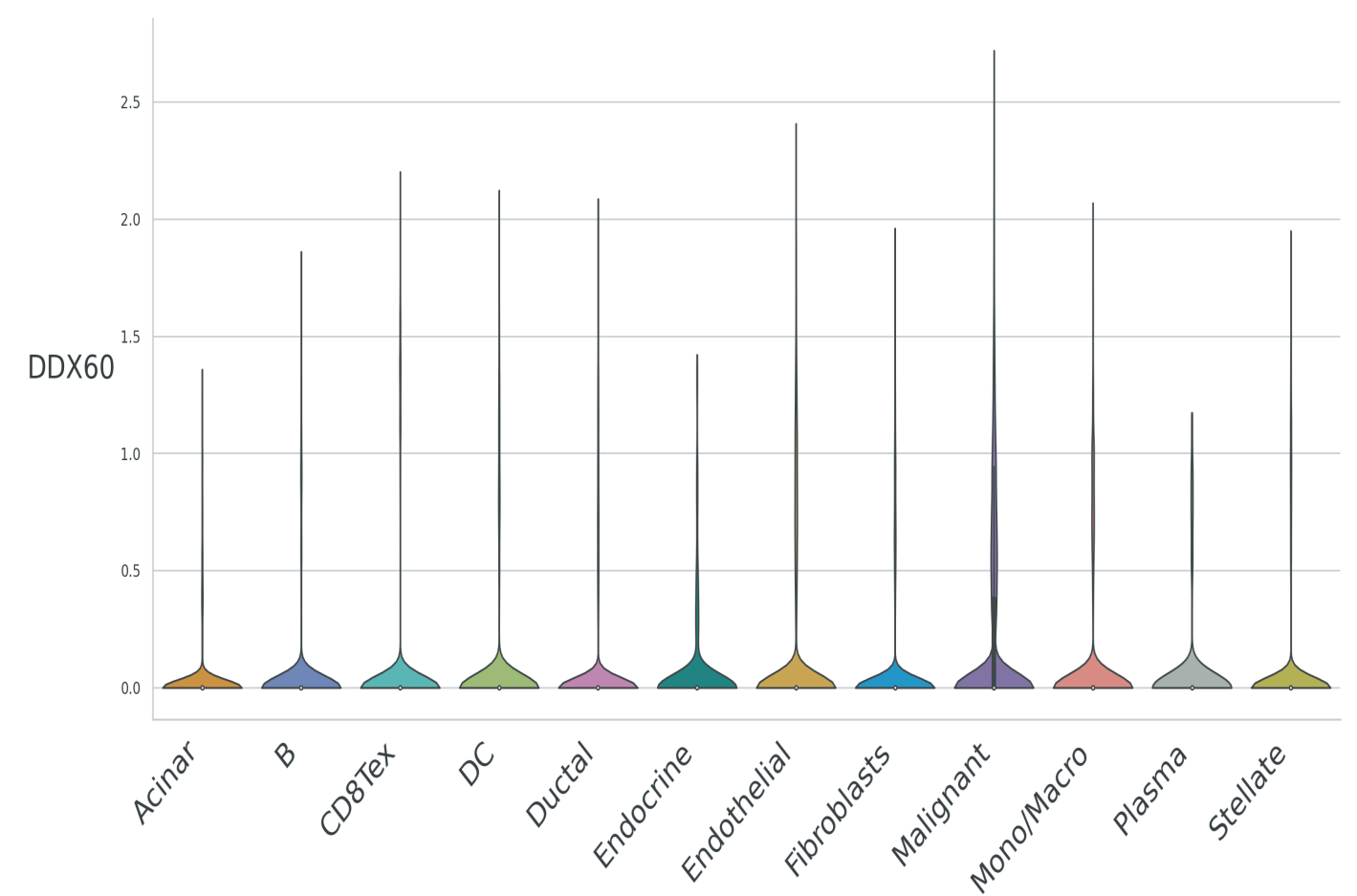

Supplement: Supplementary file 2 — Supplementary Material 2: Supplement Figure2. High expression DDX60 positive with malignant cells in PAAD_CRA001160. [file 41065_2024_361_MOESM2_ESM.pdf]

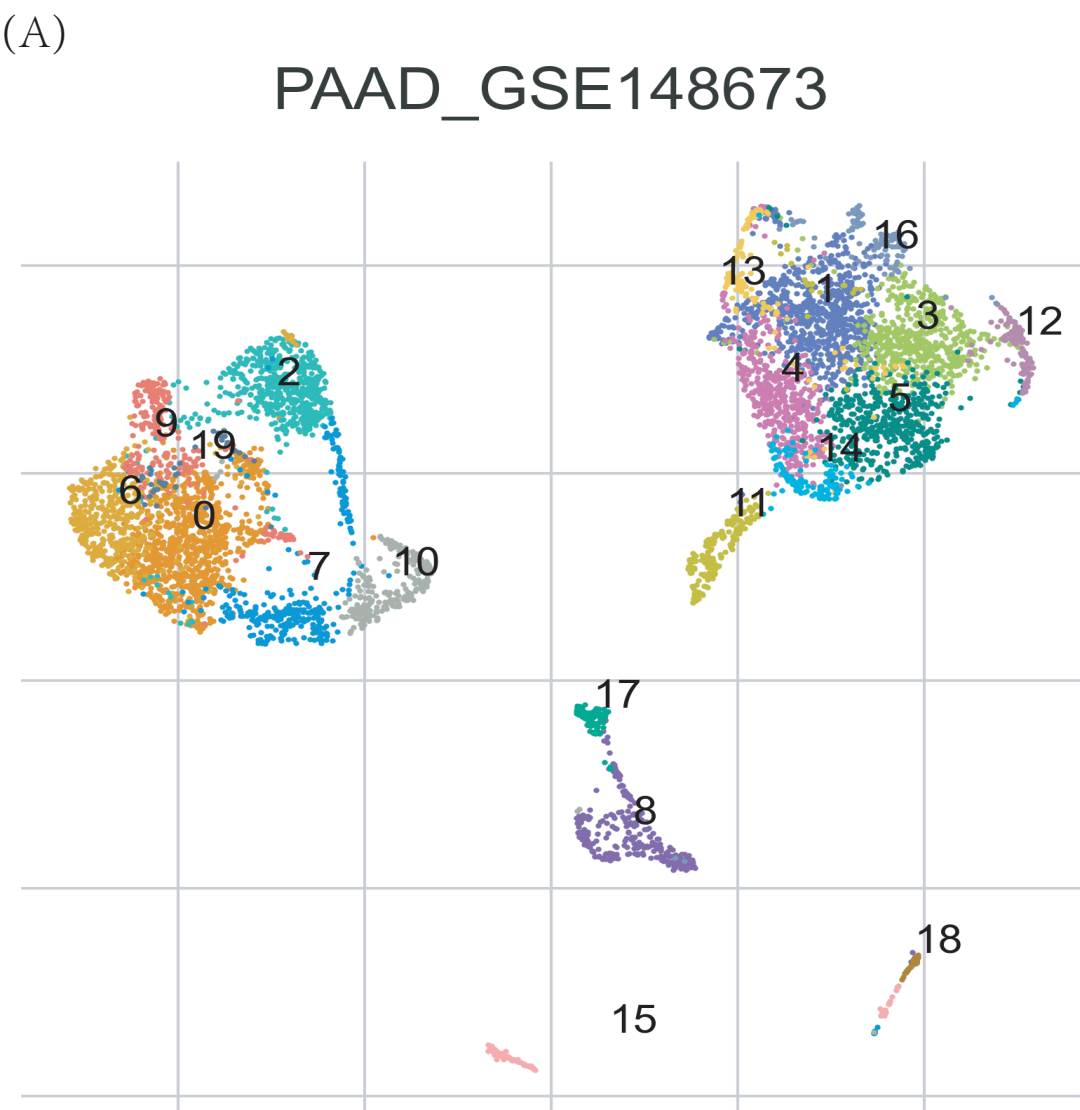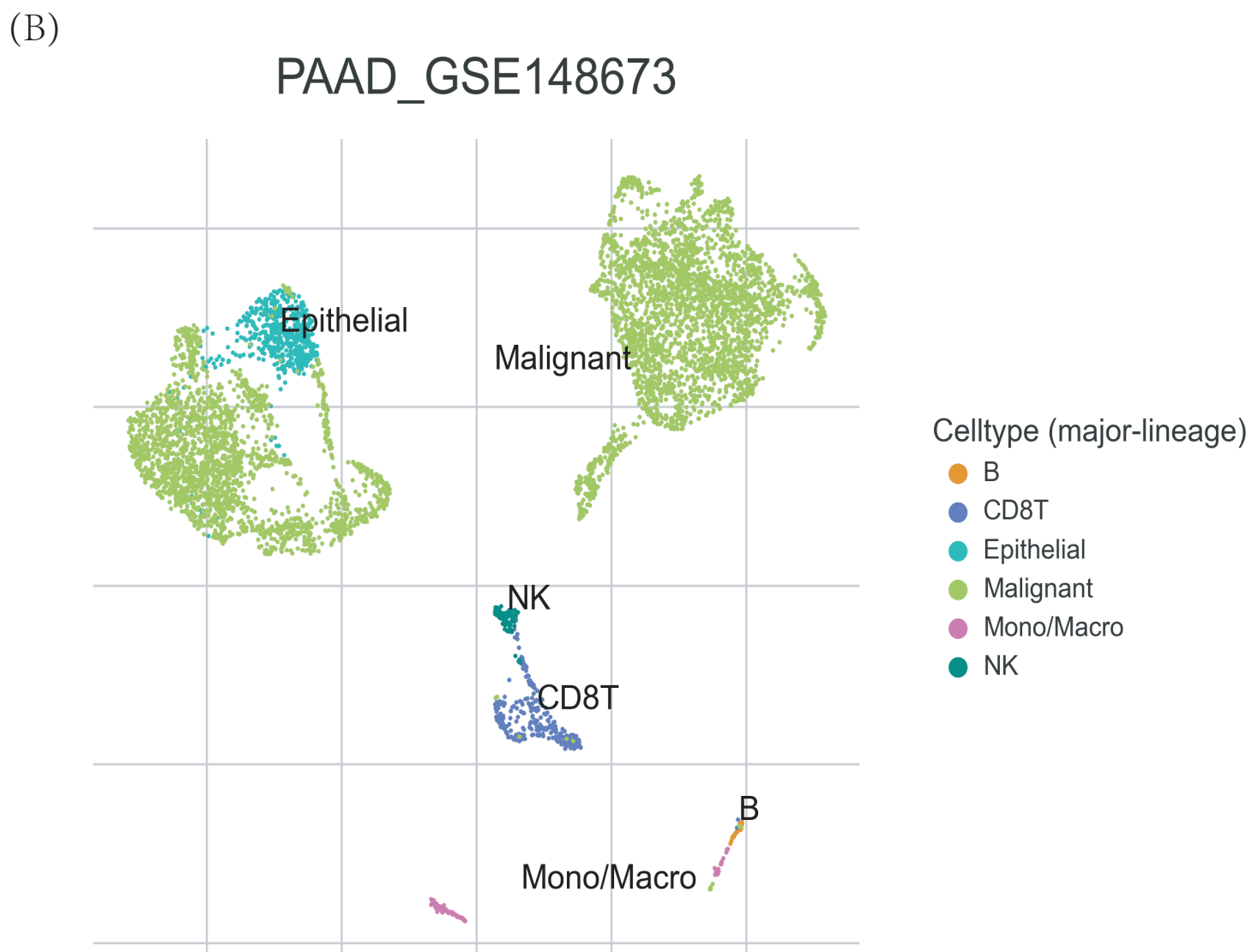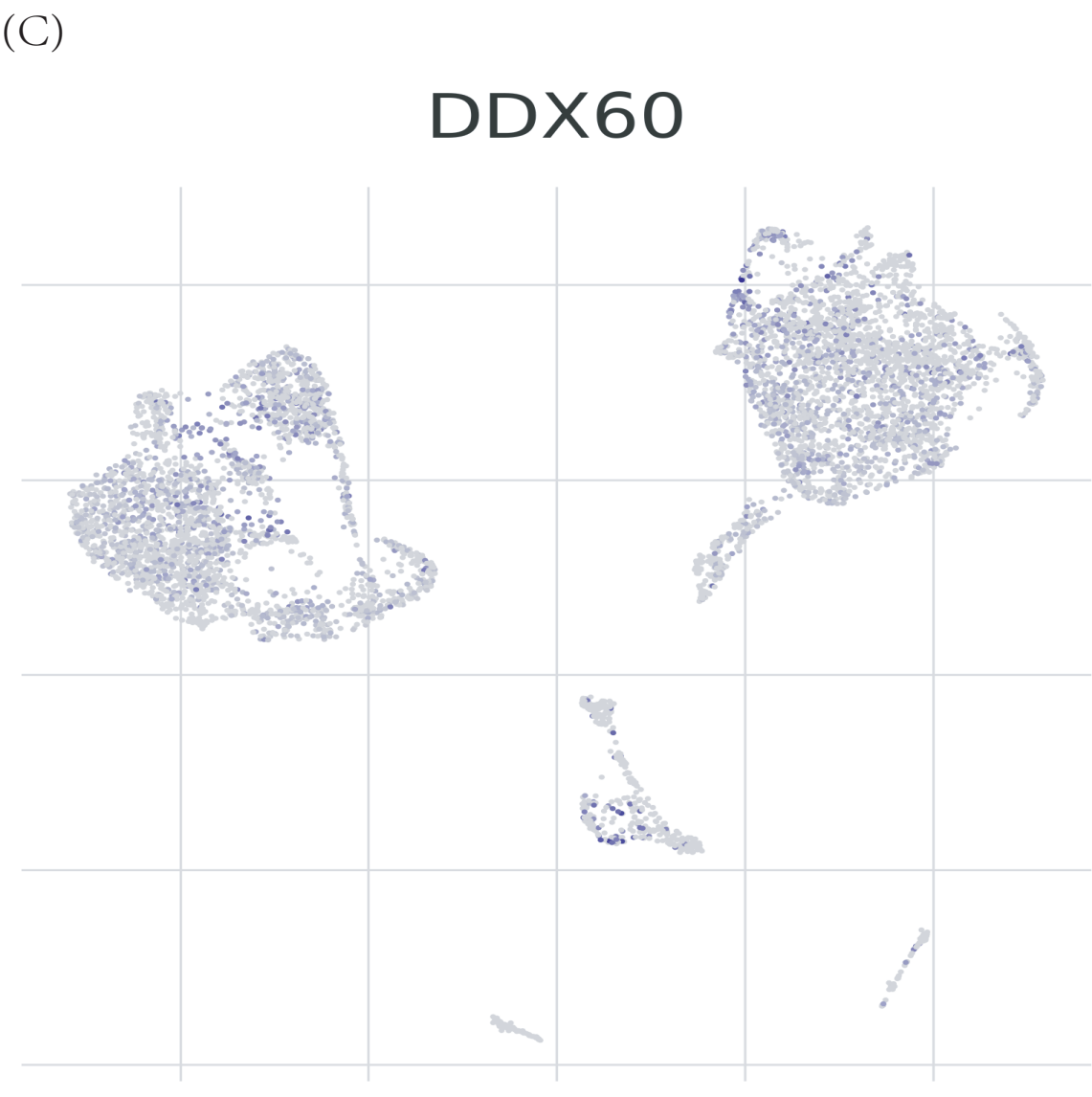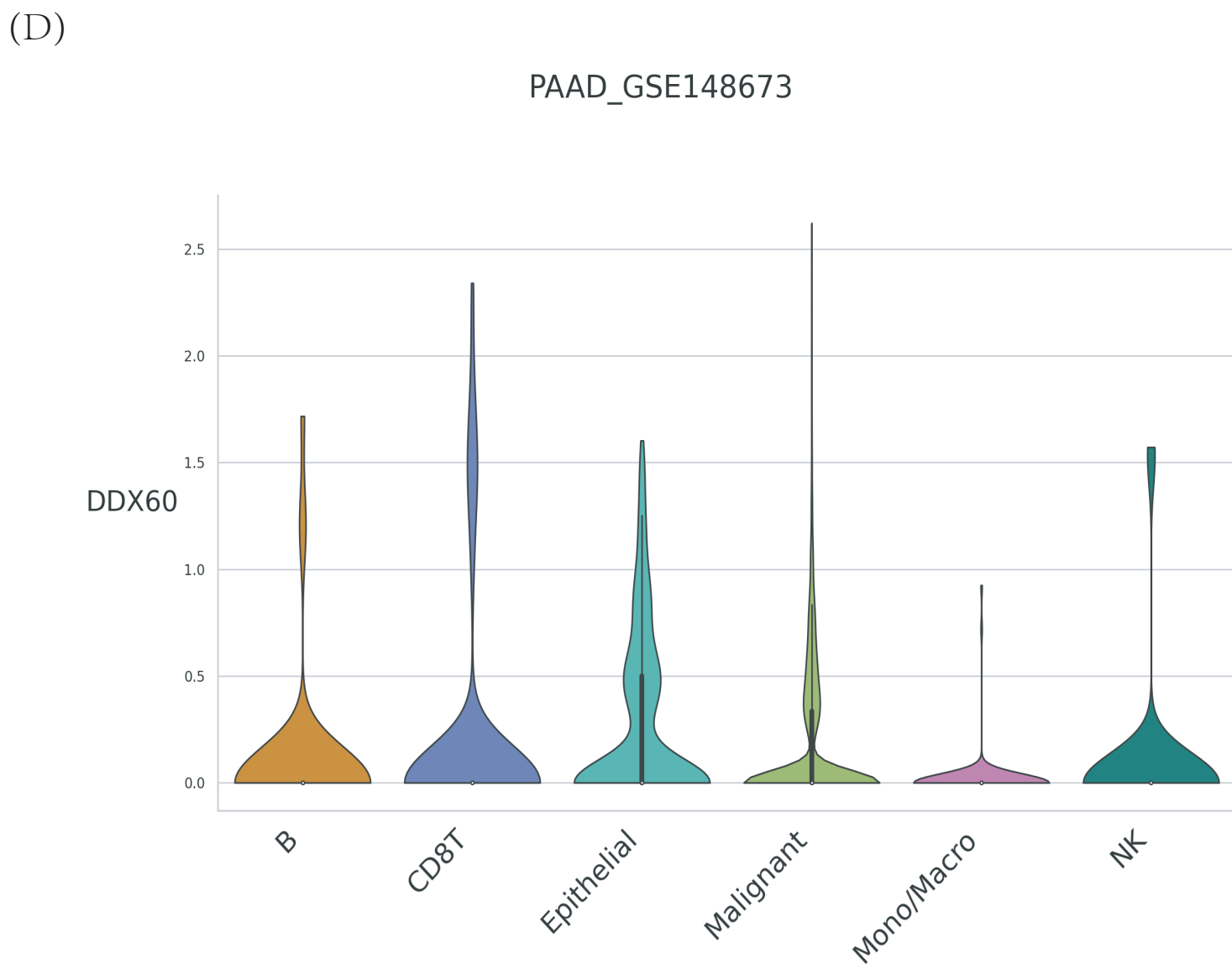

Supplement: Supplementary file 3 — Supplementary Material 3: Supplement Figure3. High expression DDX60 positive with malignant cells in PAAD_GSE148673. [file 41065_2024_361_MOESM3_ESM.pdf]

(A)

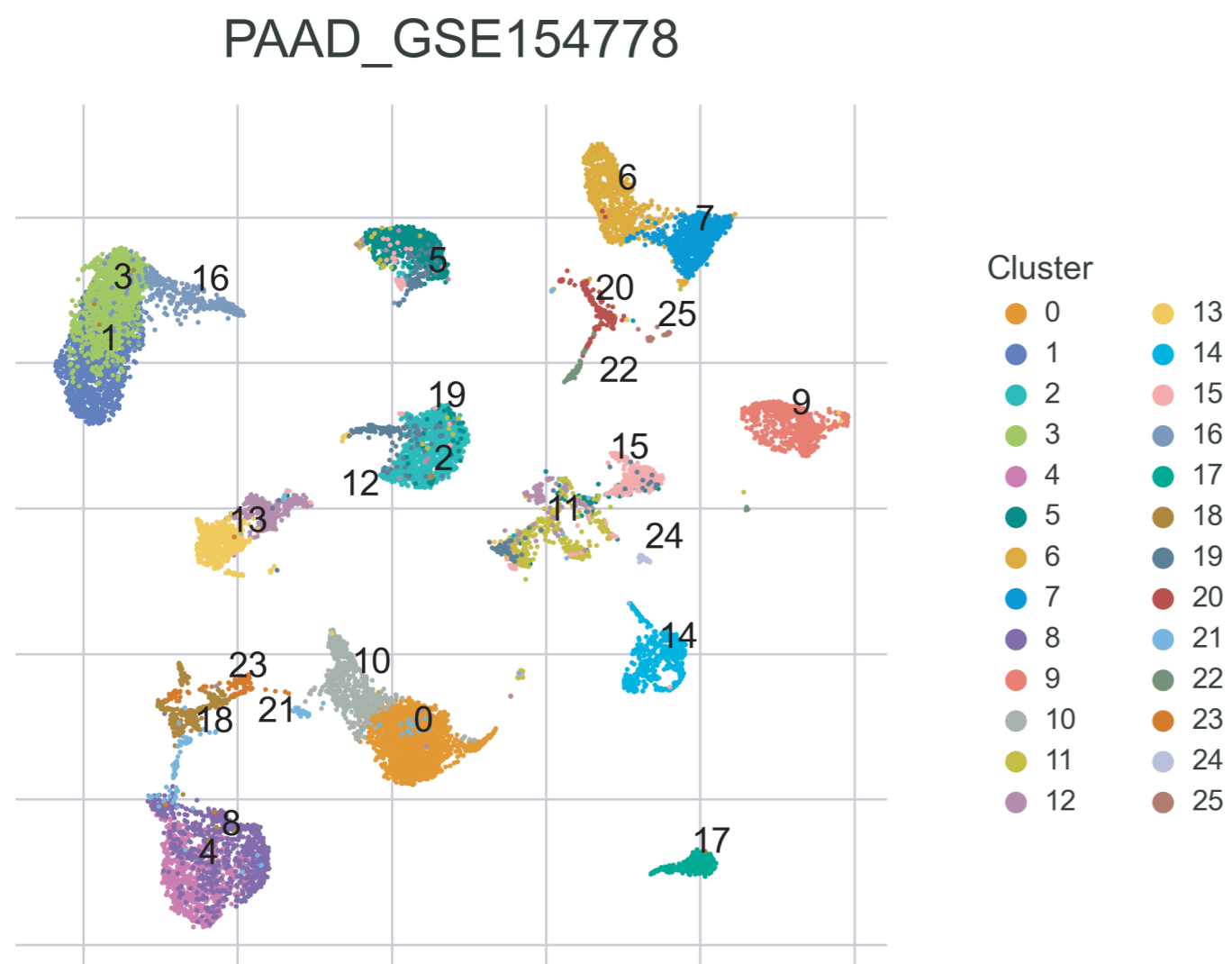

(B)

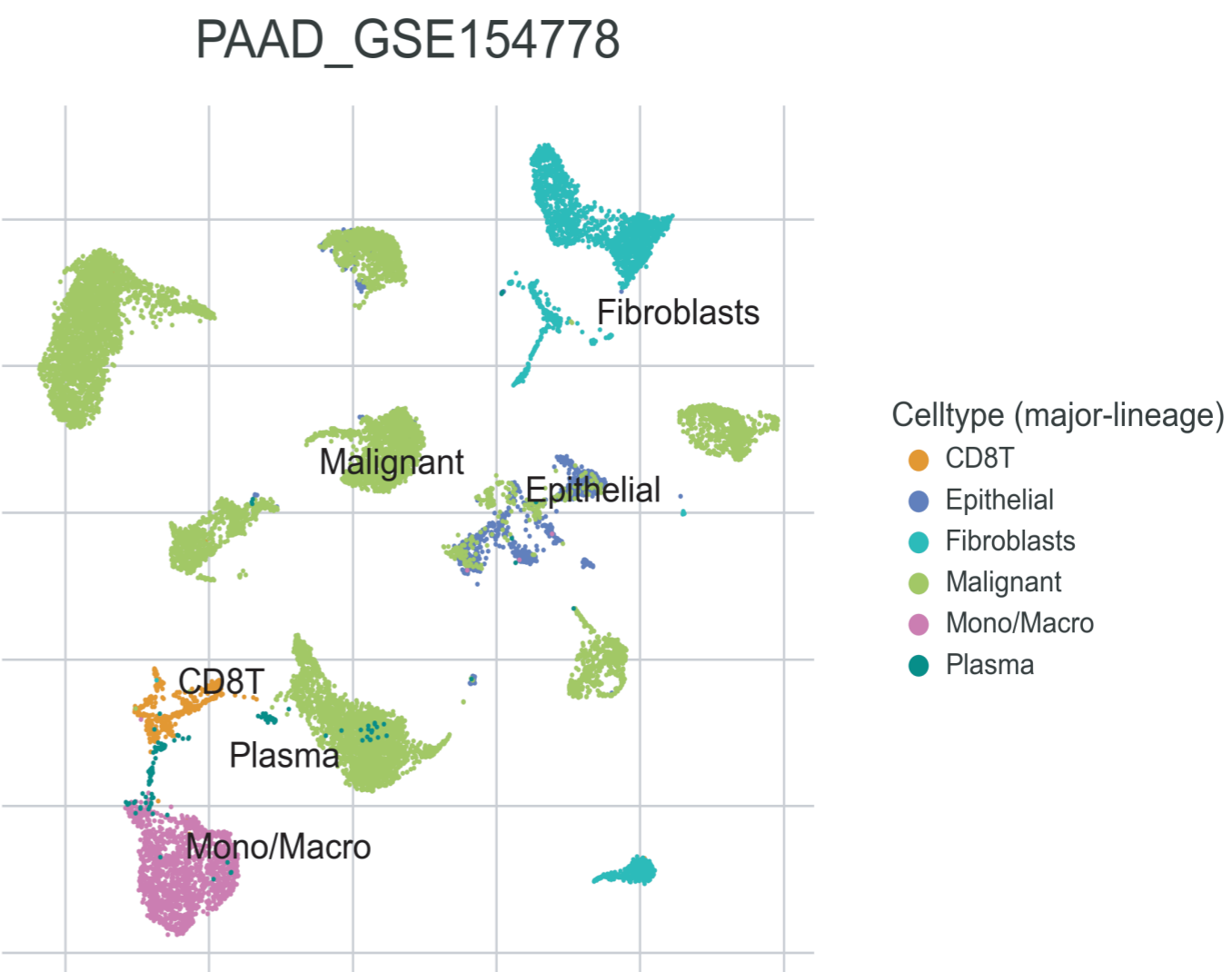

(C)

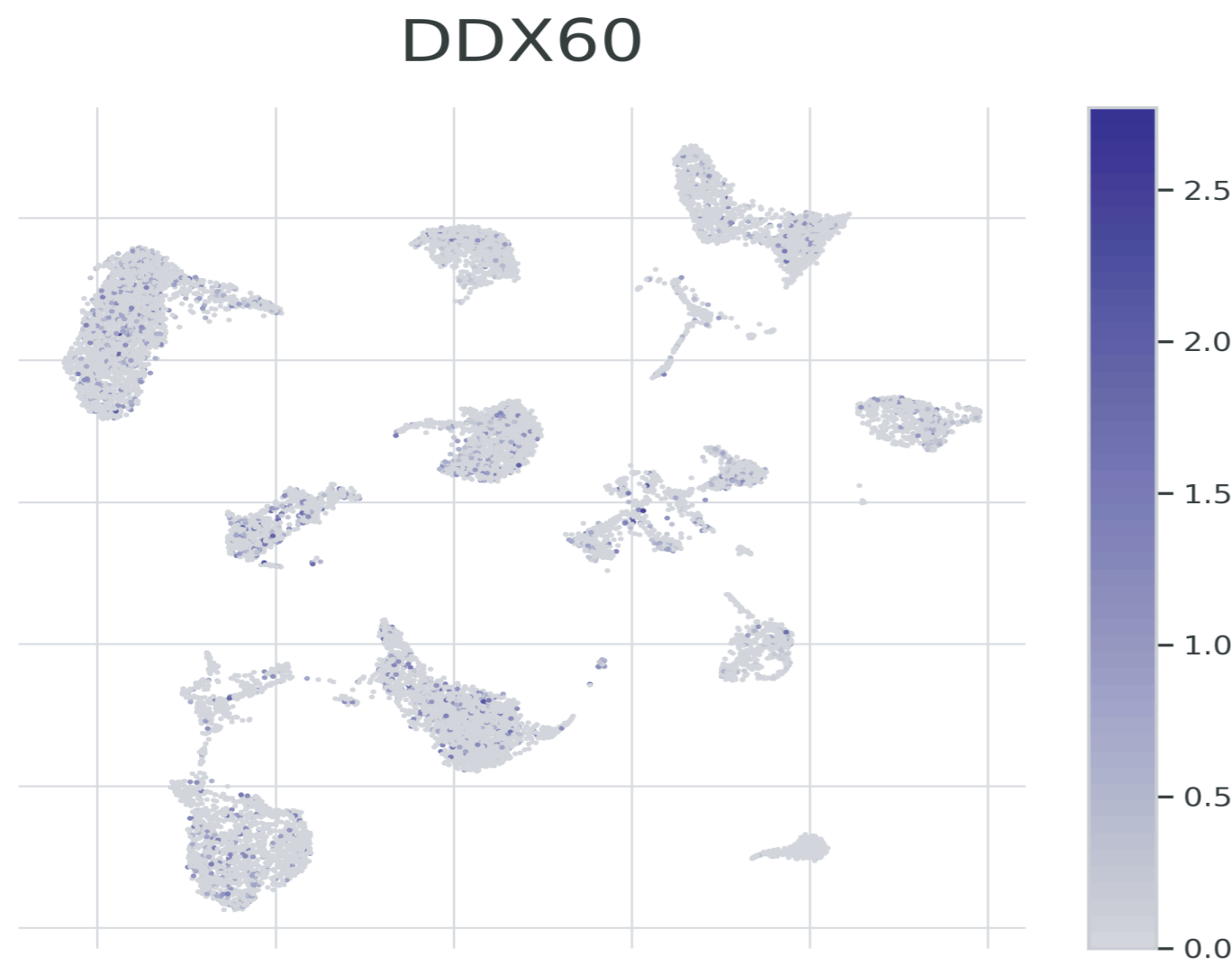

(D)

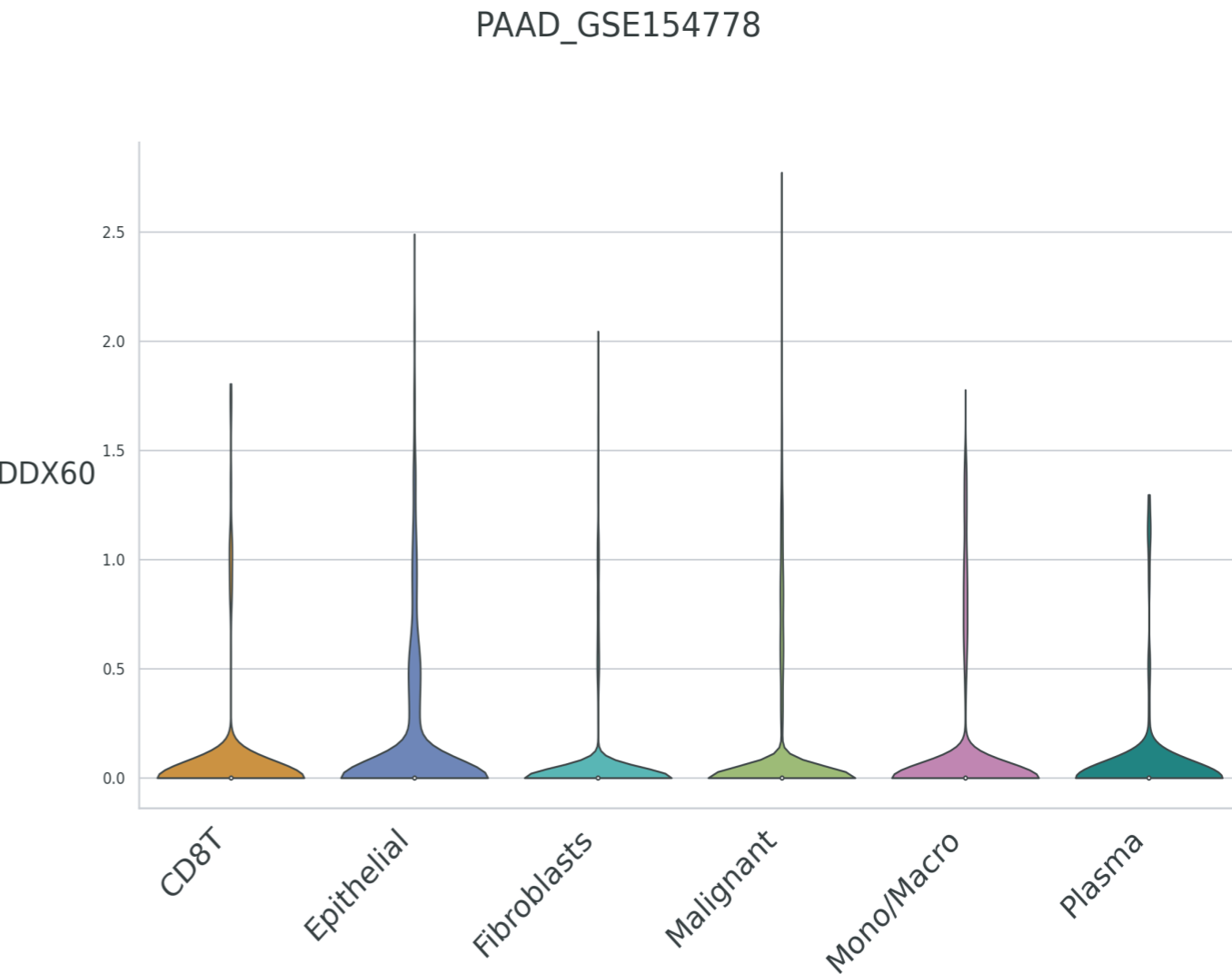

Supplement: Supplementary file 4 — Supplementary Material 4: Supplement Figure4. High expression DDX60 positive with malignant cells in PAAD_GSE154778. [file 41065_2024_361_MOESM4_ESM.pdf]

(A)

PAAD\_GSE165399

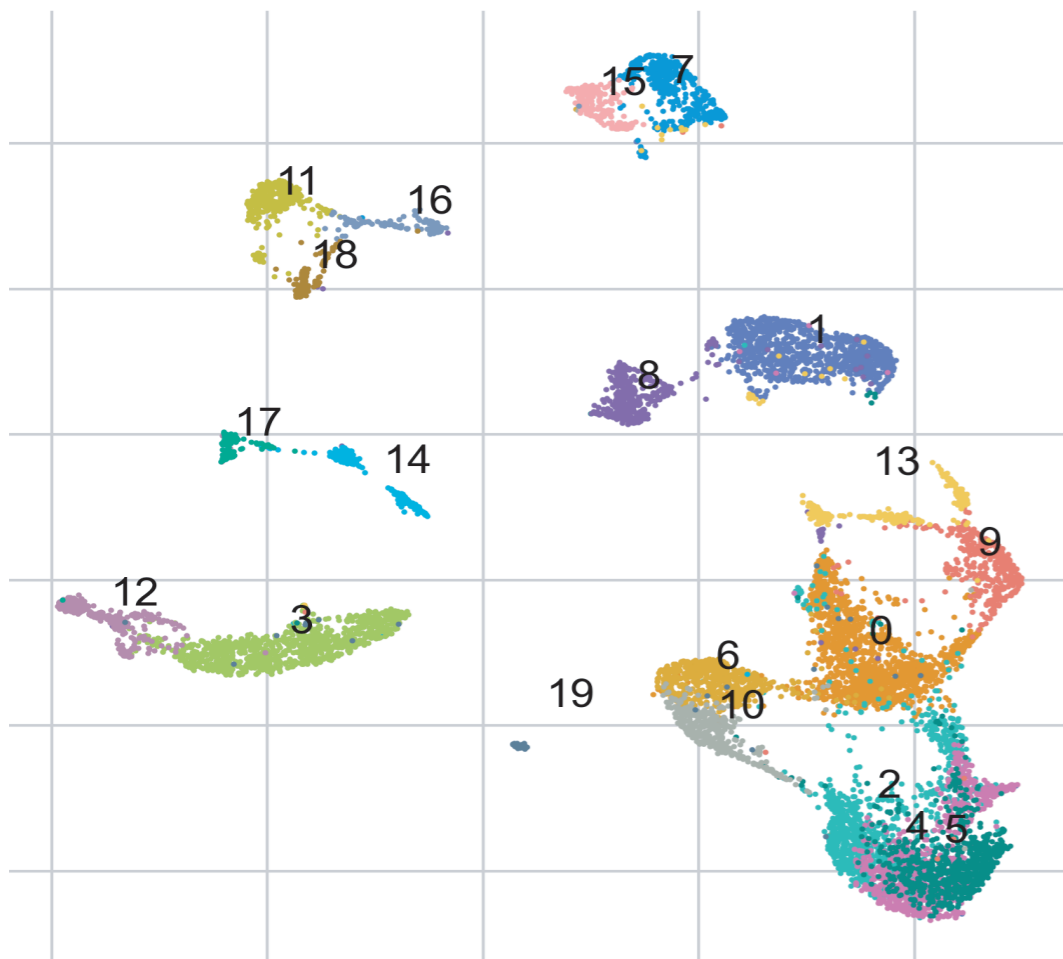

(B)

PAAD\_GSE165399

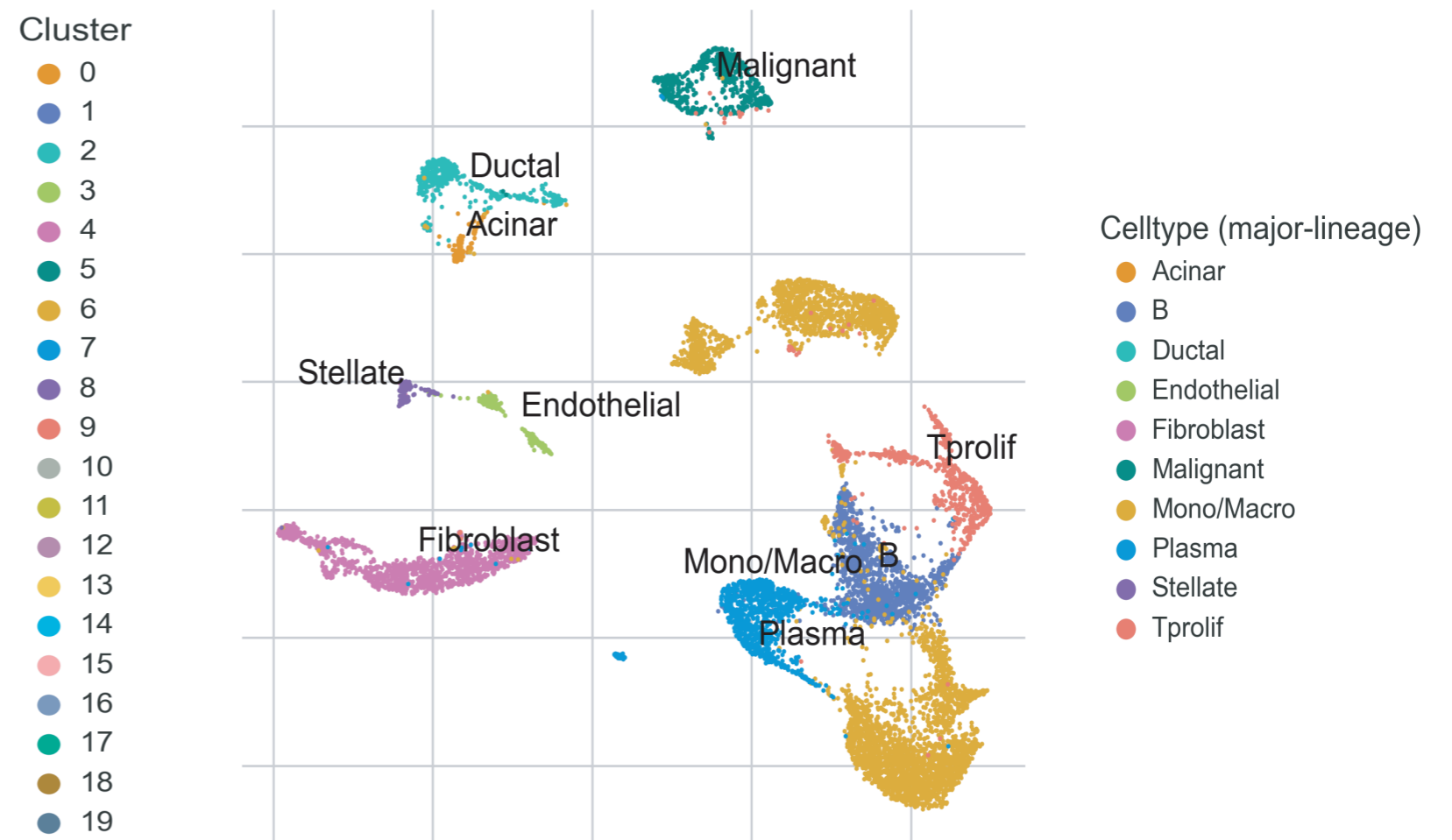

(C)

DDX60

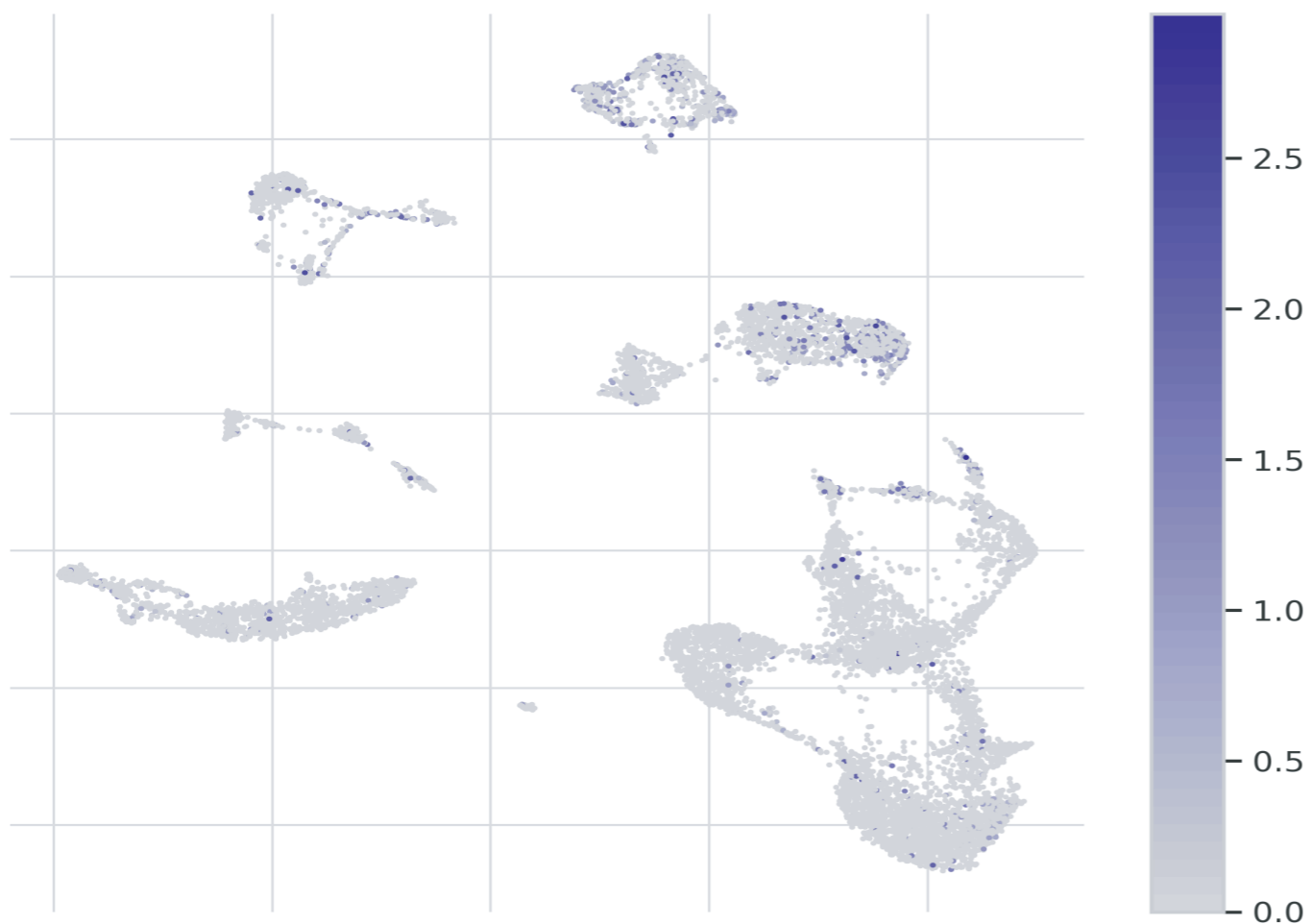

(D)

PAAD\_GSE165399

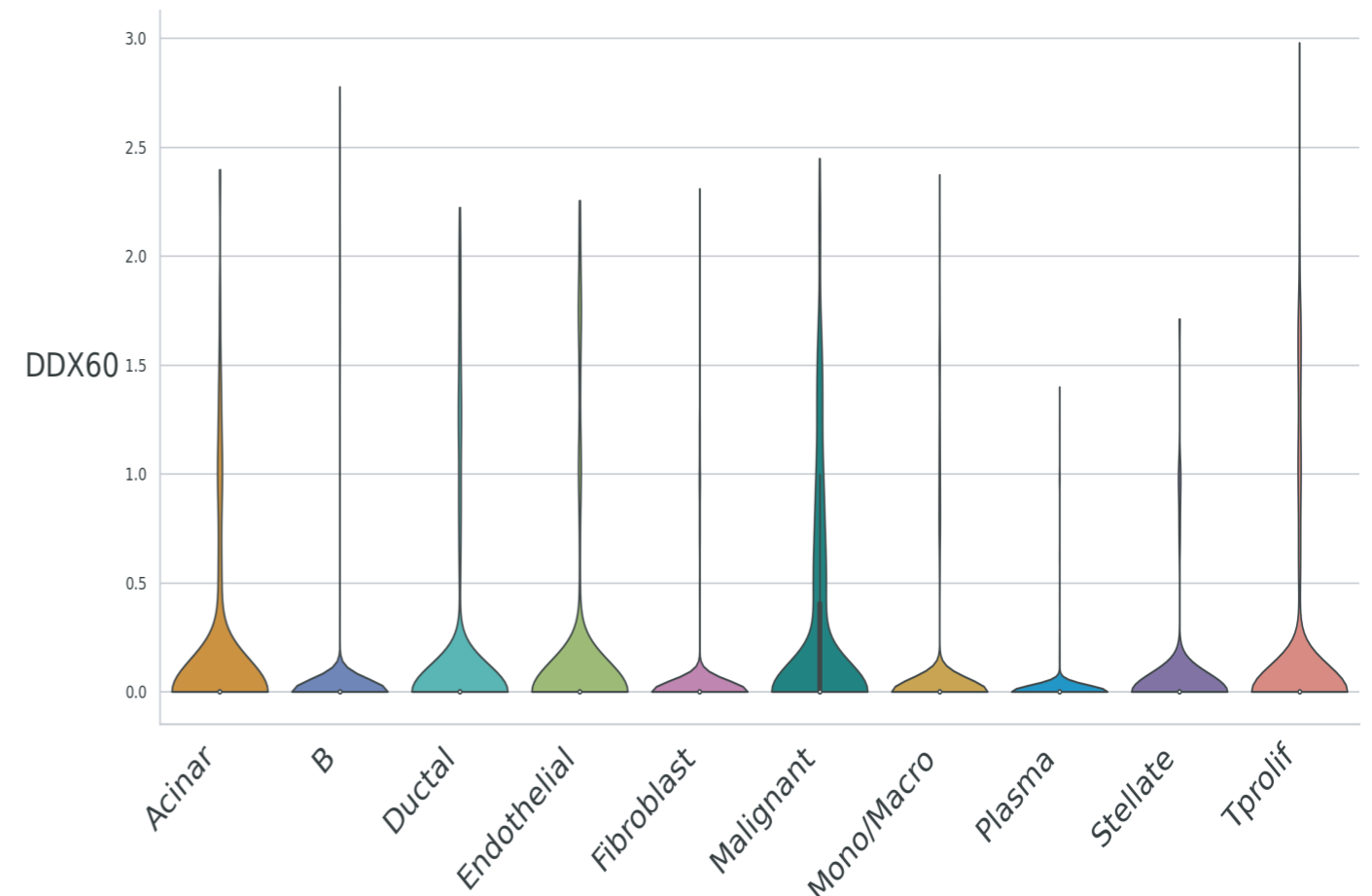

Supplement: Supplementary file 5 — Supplementary Material 5: Supplement Figure5. High expression DDX60 positive with malignant cells in PAAD_GSE165399. [file 41065_2024_361_MOESM5_ESM.pdf]

DDX60 Low High

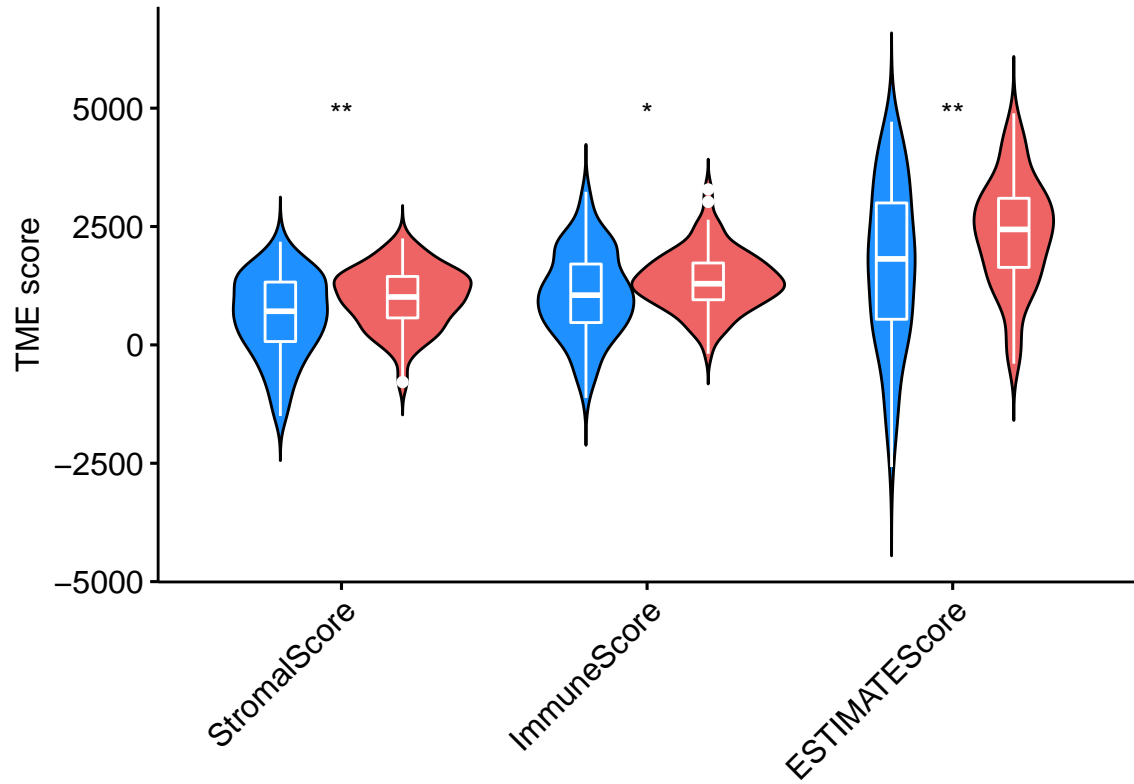

Supplement: Supplementary file 6 — Supplementary Material 6: Supplement Figure6. High expression of DDX60 promotes immune infiltration of pancreatic cancer. DDX60 was positively correlated with stromal scores, immune scores and estimate score in the tumor microenvironment. [file 41065_2024_361_MOESM6_ESM.pdf]

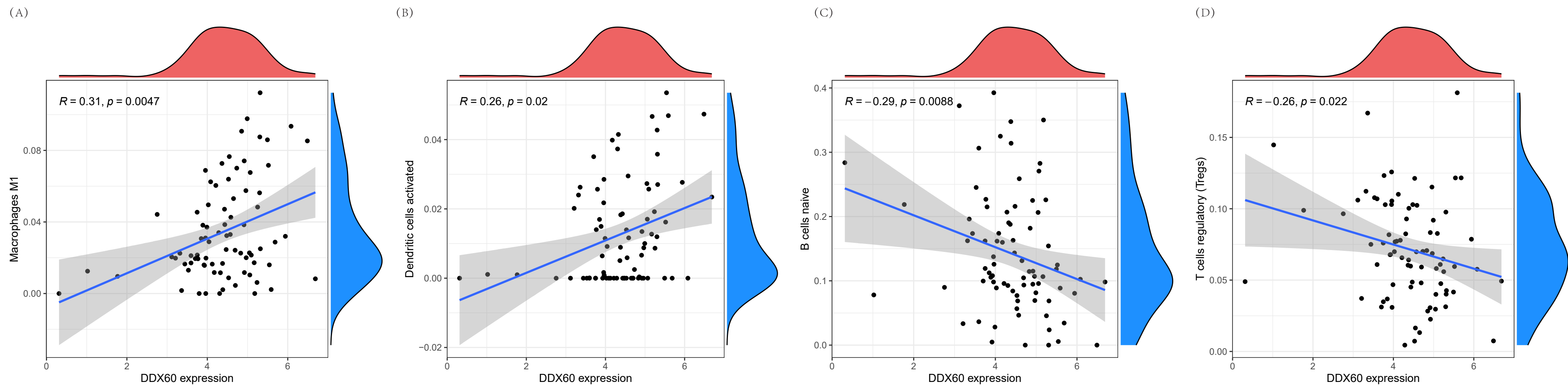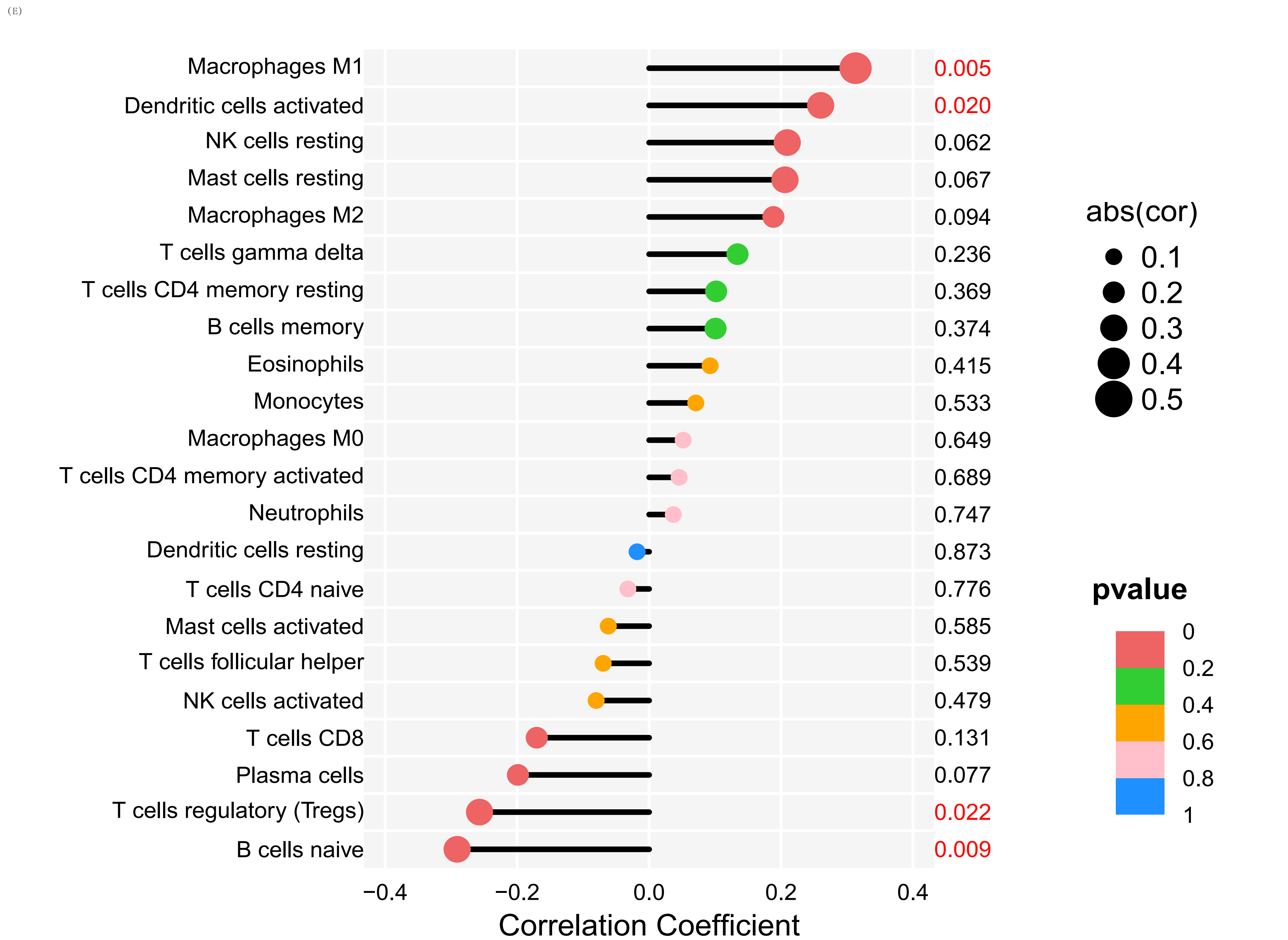

Supplement: Supplementary file 7 — Supplementary Material 7: Supplement Figure7. The relationship between DDX60 and 21 immune cells. A DDX60 positively correlated with Macrophages M1 (cor=0.31; P=0.0047). B DDX60 positively correlated with dendritic cell activated (cor=0.26; P=0.02). C DDX60 negatively correlated with B cells naive (cor=-0.29; P=0.0088). D DDX60 negatively correlated with T cells regulatory (Tregs) (cor=-0.26; P=0.022). E A lollipop chart of the relationship between DDX60 and 21 immune cells in pancreatic cancer. [file 41065_2024_361_MOESM7_ESM.pdf]

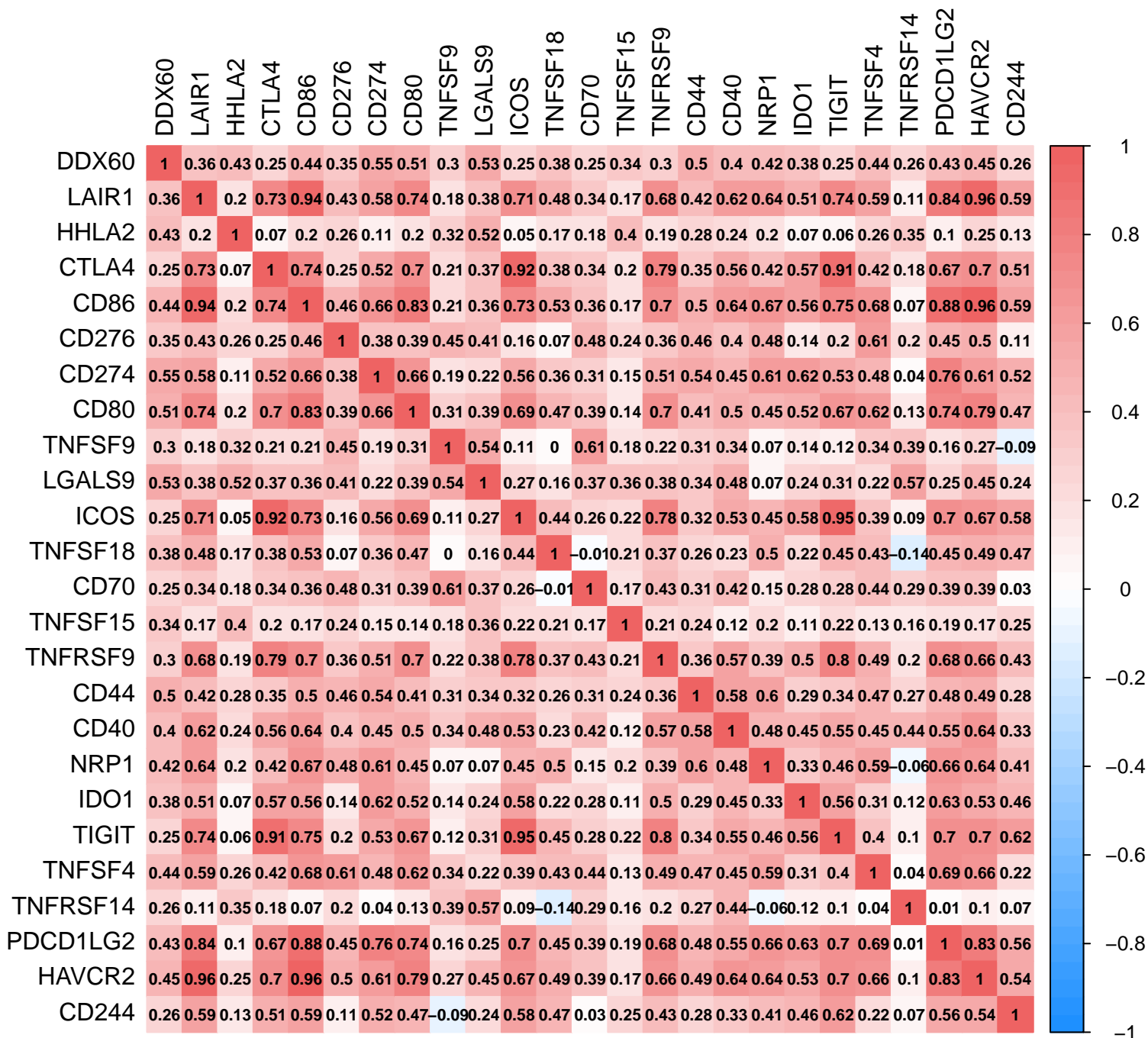

Supplement: Supplementary file 8 — Supplementary Material 8: Supplement Figure8. The relationship between DDX60 and immune checkpoints. DDX60 was positively correlated with immune checkpoint CD274，LGALS9, CD80, CD44, HAVCR2, CD86, TNFSF4, HHLA2, PDCD1LG2, NRP1, CD40, IDO1, TNFSF18, LAIR1, CD276, TNFSF15, TNFRSF9, TNFSF9, TNFRSF14, CD244, CD70, CTLA4, ICOS, and TIGIT (P＜0.05). [file 41065_2024_361_MOESM8_ESM.pdf]

(A)

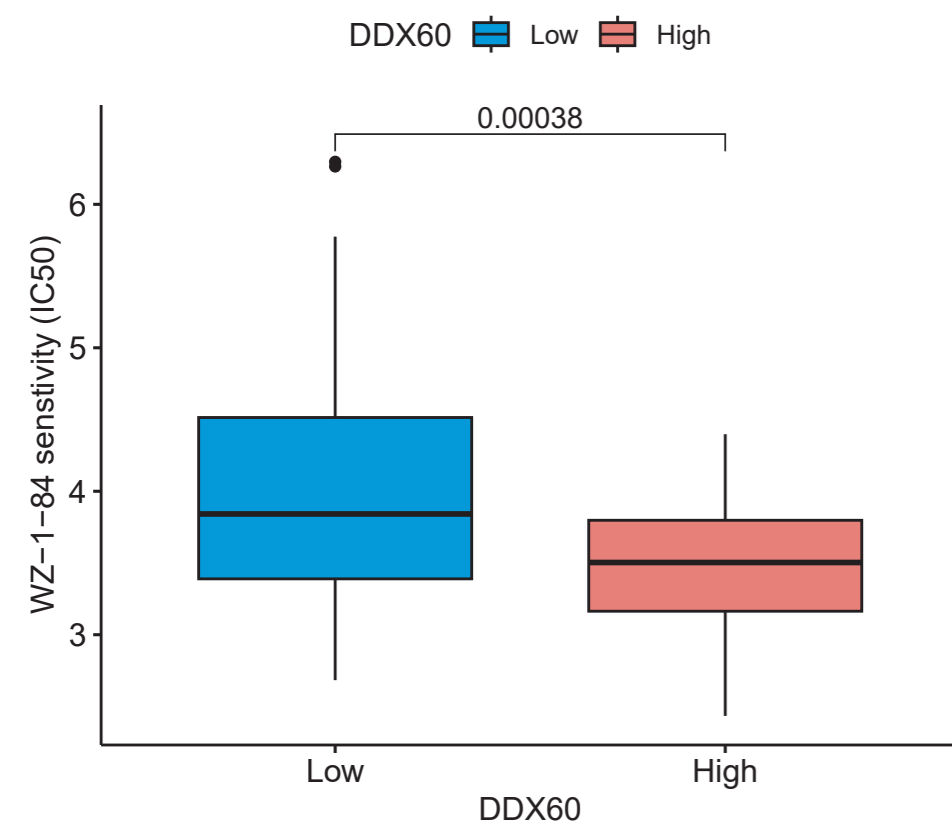

(B)

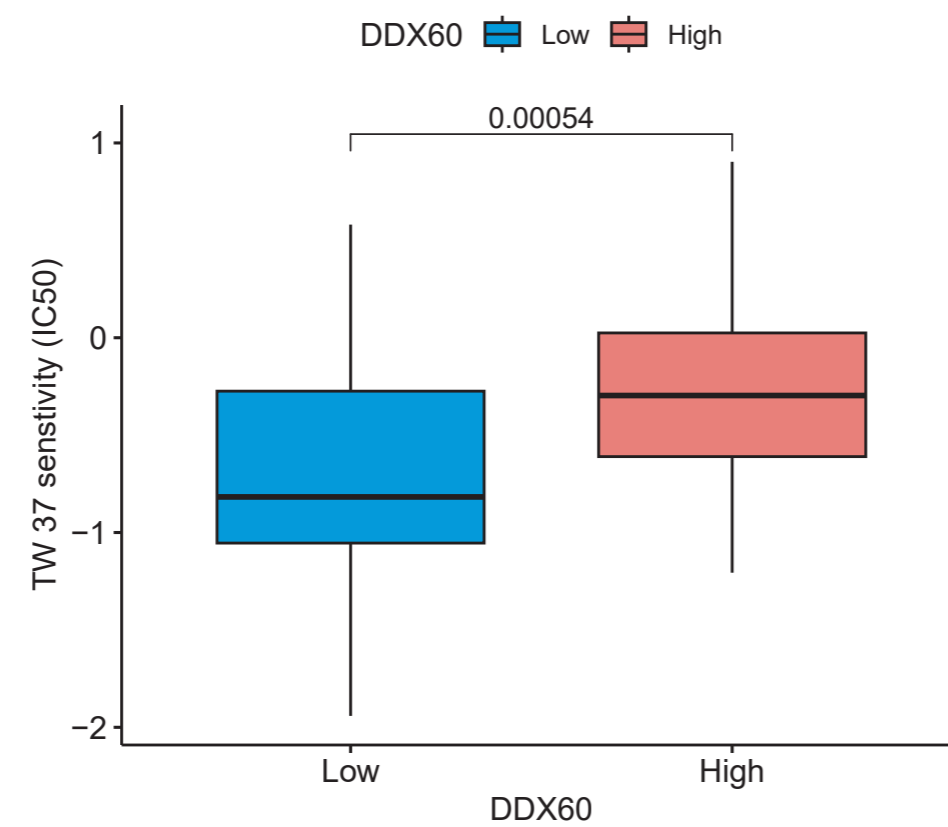

(C)

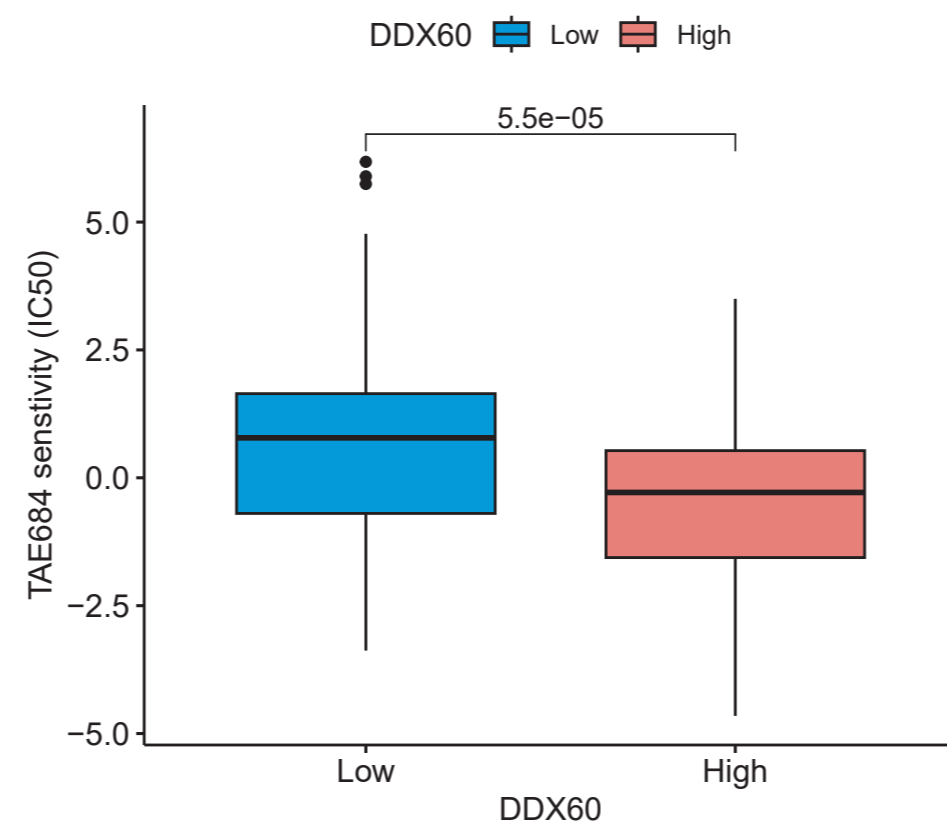

(D)

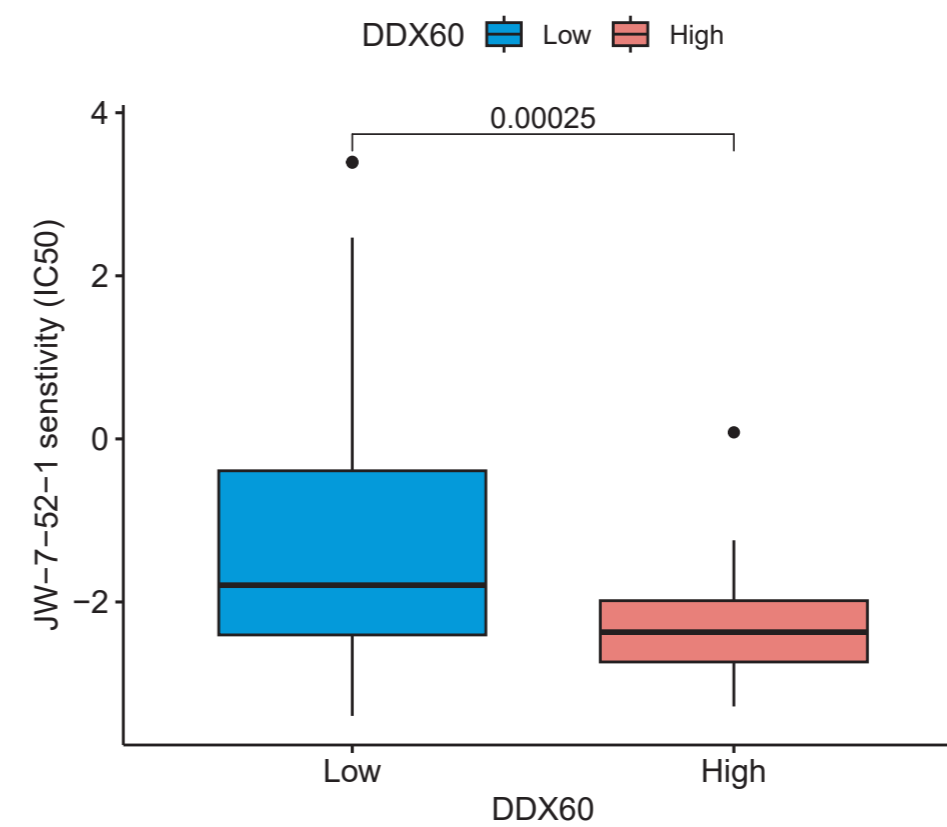

(E)

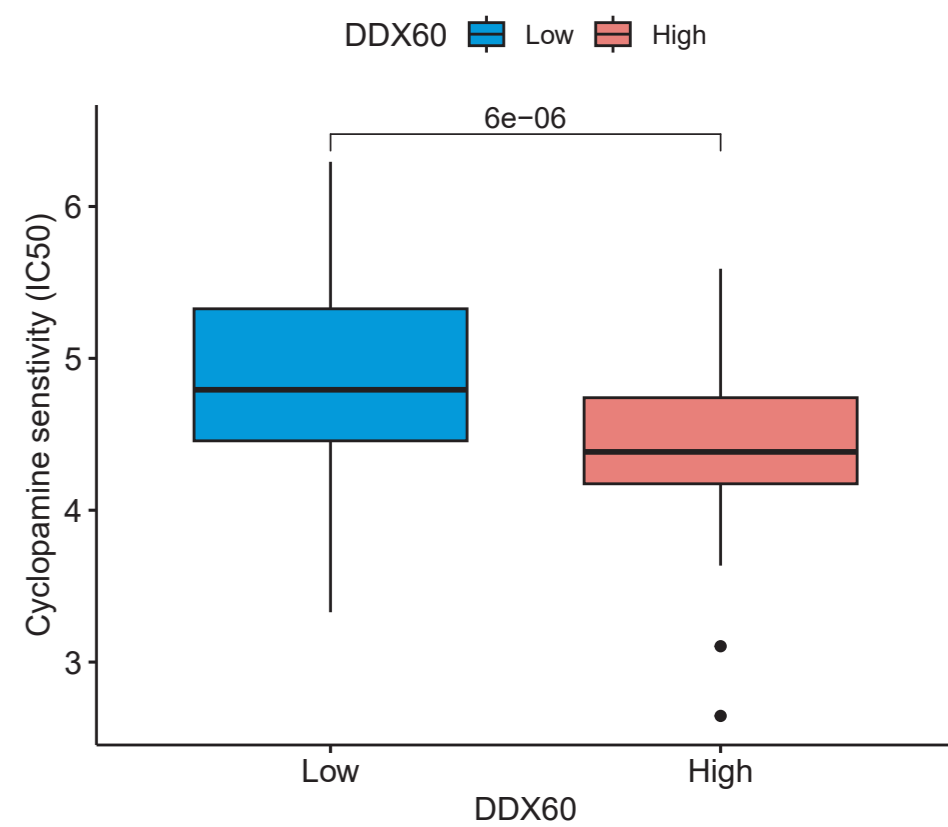

(F)

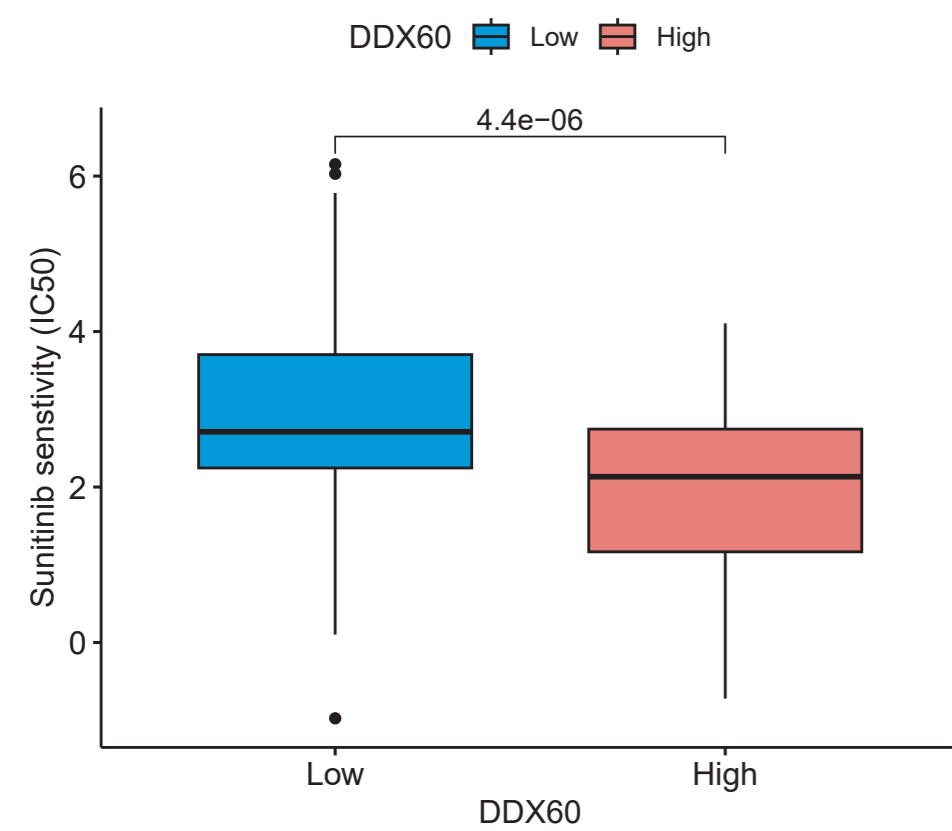

(G)

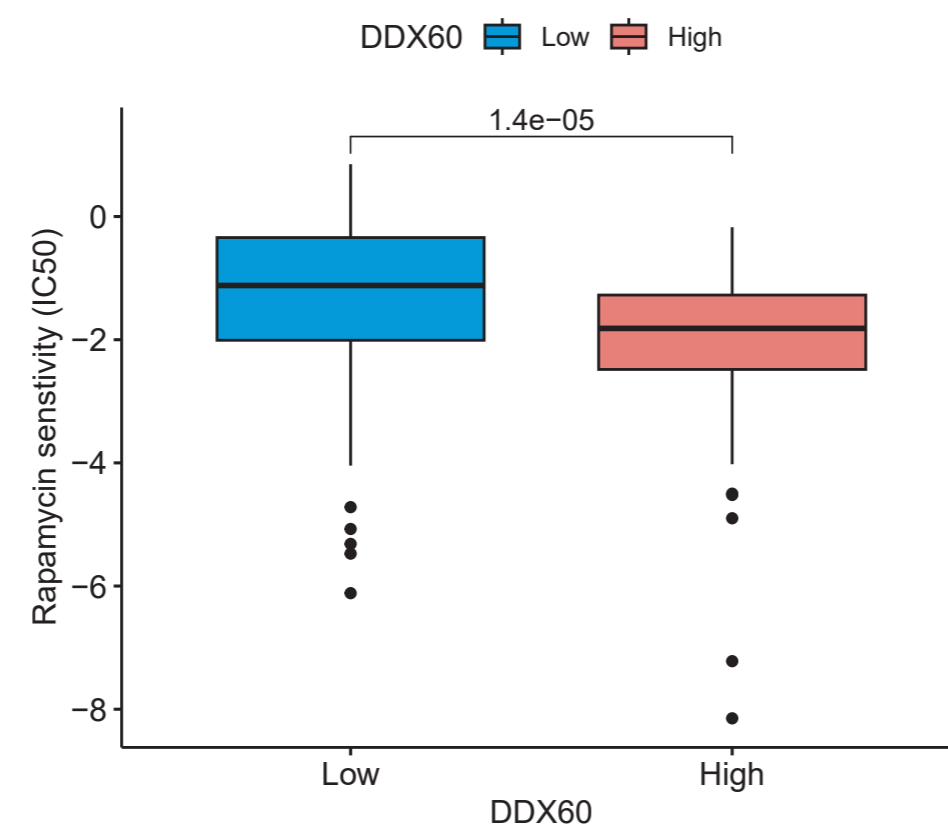

(H)

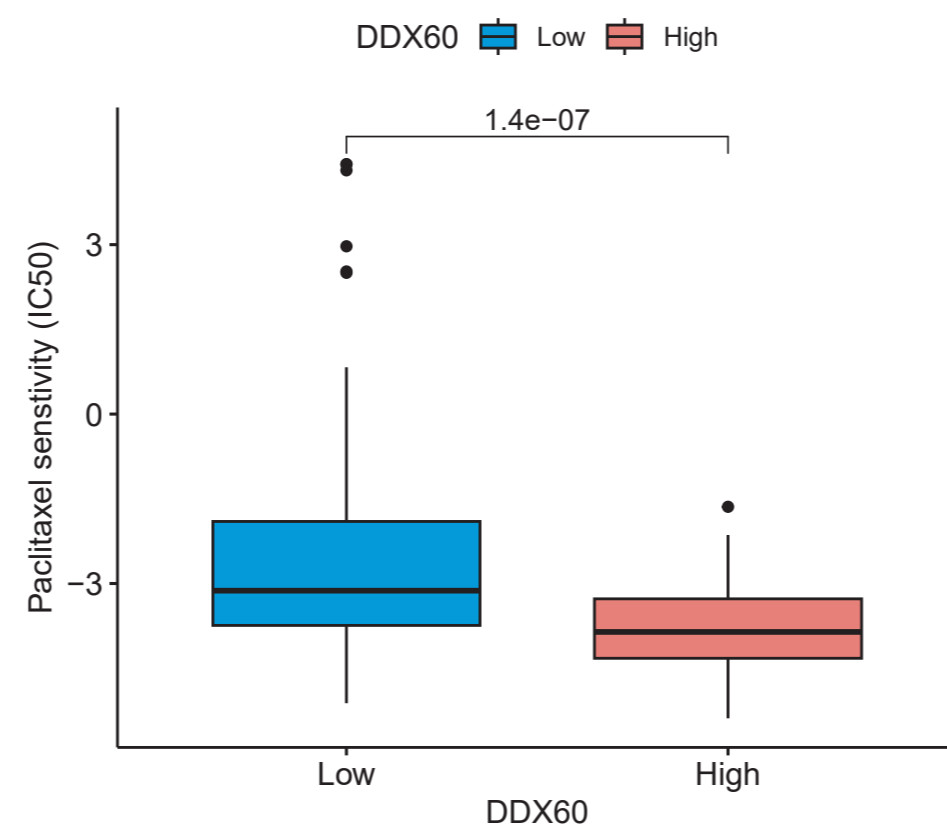

(I)

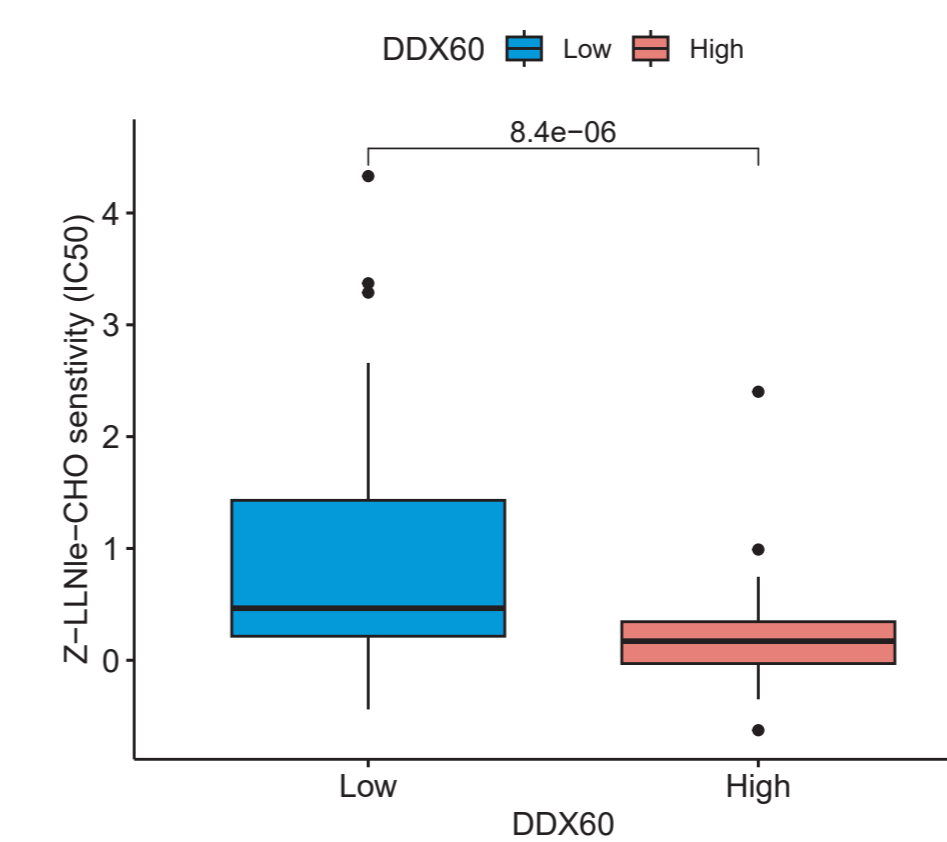

(J)

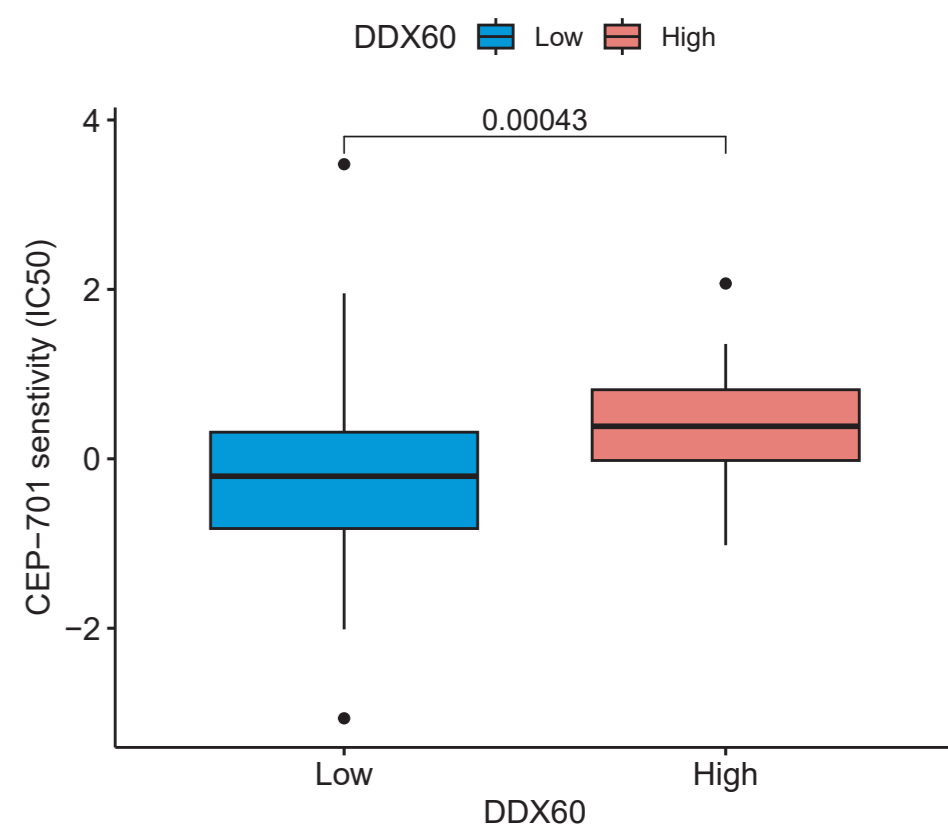

Supplement: Supplementary file 9 — Supplementary Material 9: Supplement Figure9. The relationship between DDX60 and 132 chemotherapeutic drugs contained in “pRRophetic” package. There was IC50 sensitivity difference between 8 chemotherapy drugs and DDX60 high risk group and low risk group. (A)PAAD patients in the DDX60 high-risk group were more sensitive to WZ-1-84 (P=0.00038). (B)PAAD patients in the DDX60 low-risk group were more sensitive to TW37 (P=0.00054). (C)PAAD patients in the DDX60 high-risk group were more sensitive to TAE684 (P=5.5e-05). (D)PAAD patients in the DDX60 high-risk group were more sensitive to JW-7-52-1 (P=0.00025). (E)PAAD patients in the DDX60 high-risk group were more sensitive to Cyclopamine (P=6e-06). (F)PAAD patients in the DDX60 high-risk group were more sensitive to Sunitinib (P=4.4e-06). (G)PAAD patients in the DDX60 high-risk group were more sensitive to Rapamycin (P=1.4e-05). (H)PAAD patients in the DDX60 high-risk group were more sensitive to Paclitaxel (P=1.4e-07). (I)PAAD patients in the DDX60 high-risk group were more sensitive to Z-LLNle-CHO(P=8.4e-06). (J)PAAD patients in the DDX60 low-risk group were more sensitive to CEP-701(P=0.00043). [file 41065_2024_361_MOESM9_ESM.pdf]

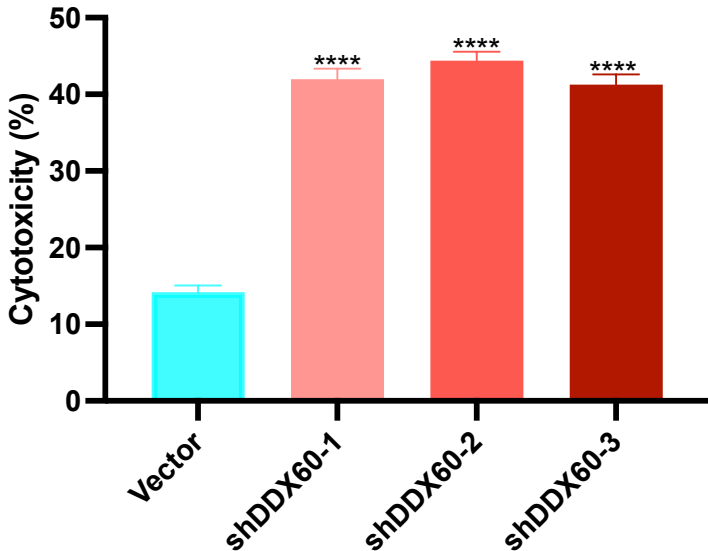

Supplement: Supplementary file 10 — Supplementary Material 10: Supplement Figure10. LDH results confirmed that pancreatic cancer cell lines in the knockdown group were more sensitive to Dabrafenib. [file 41065_2024_361_MOESM10_ESM.pdf]

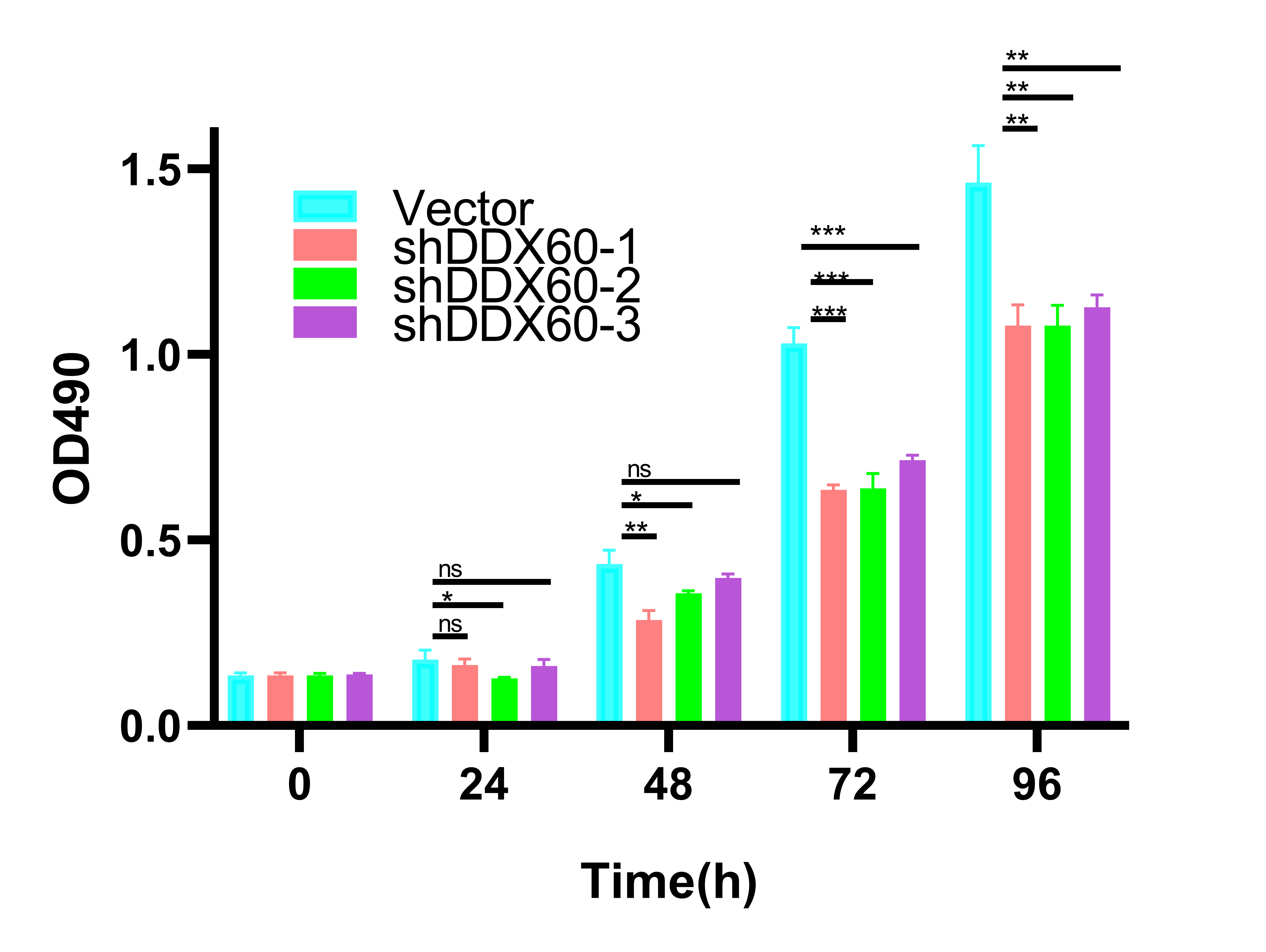

Supplement: Supplementary file 11 — Supplementary Material 11. [file 41065_2024_361_MOESM11_ESM.tif]

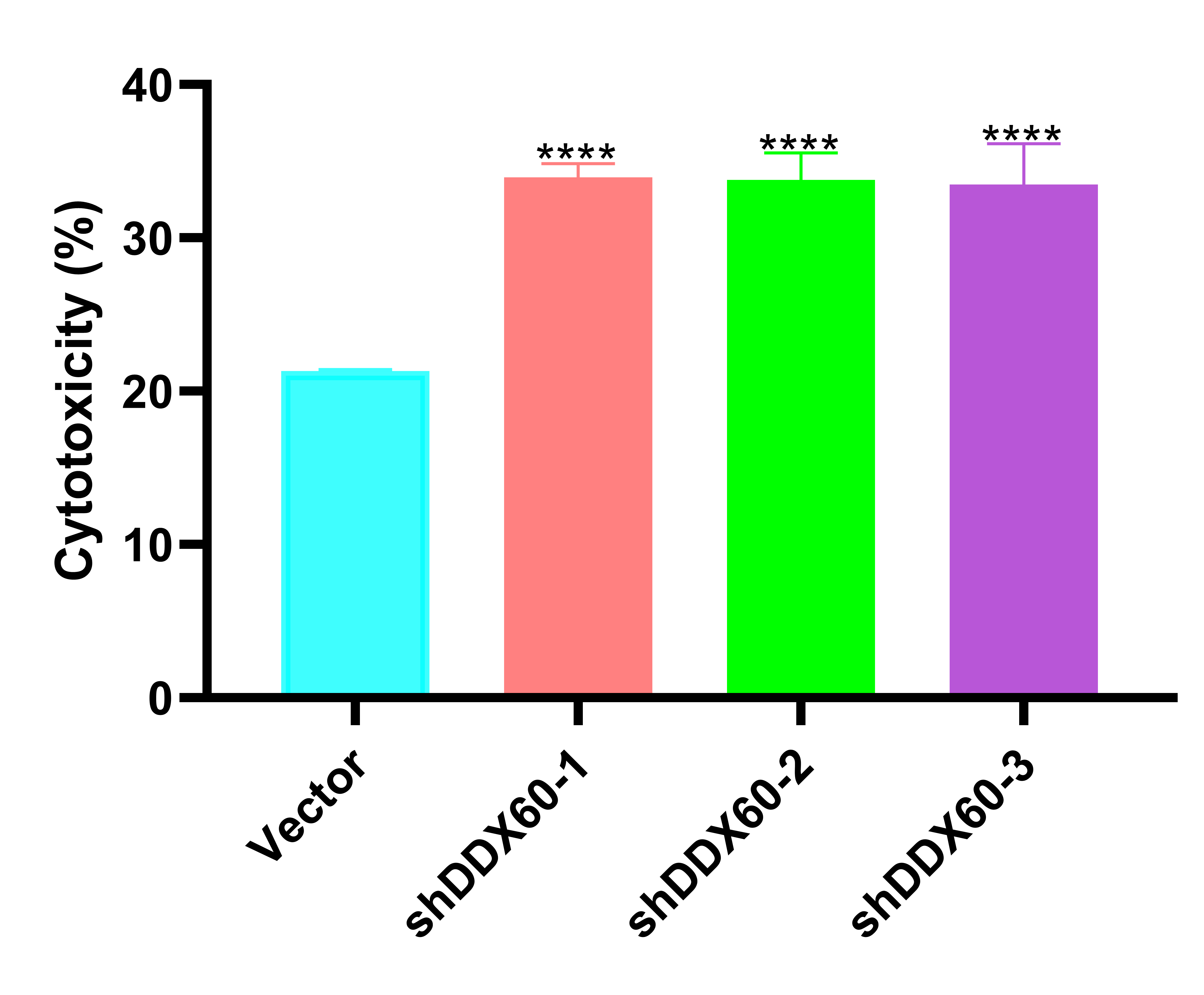

Supplement: Supplementary file 12 — Supplementary Material 12. [file 41065_2024_361_MOESM12_ESM.tif]

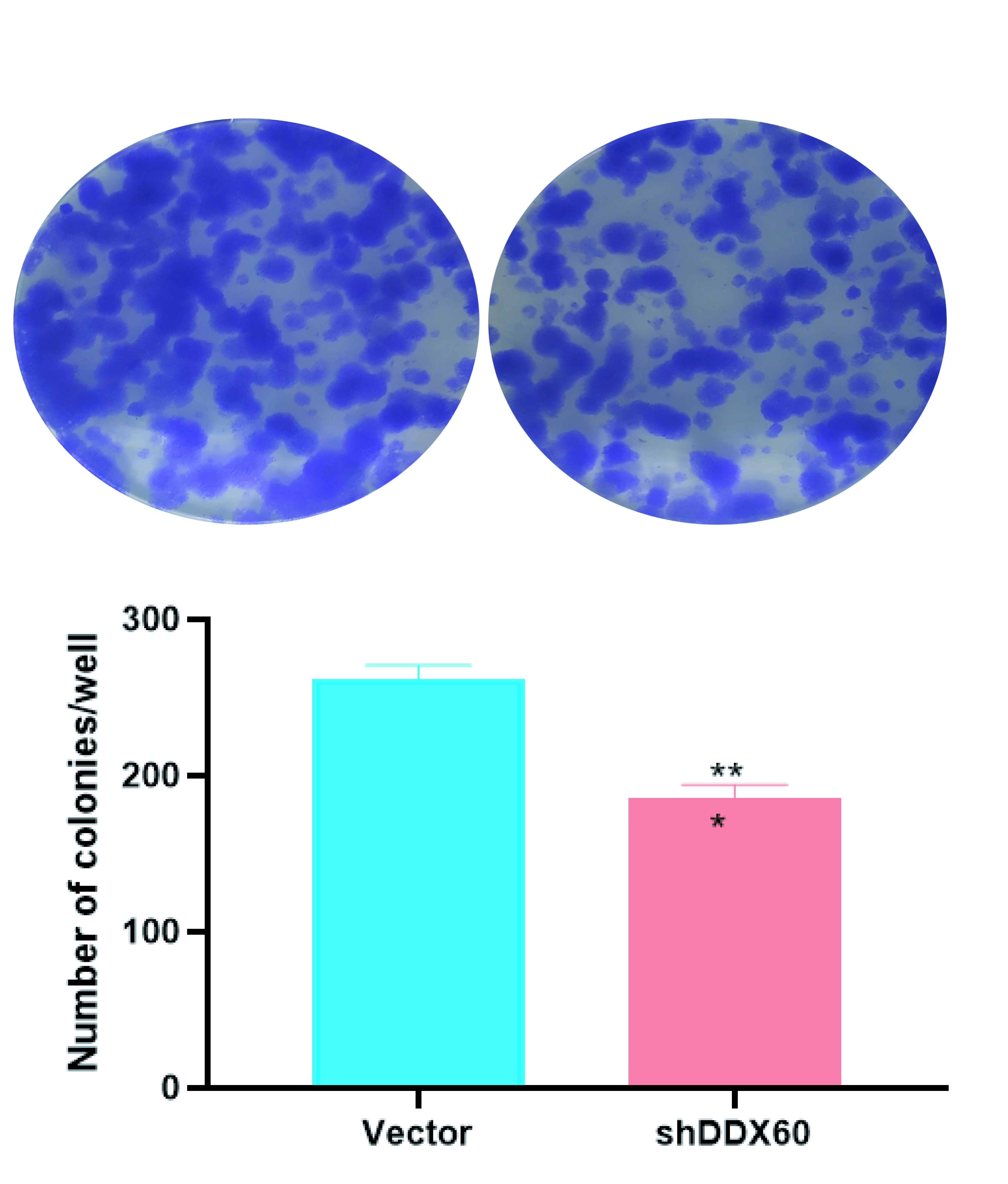

Supplement: Supplementary file 13 — Supplementary Material 13. [file 41065_2024_361_MOESM13_ESM.tif]

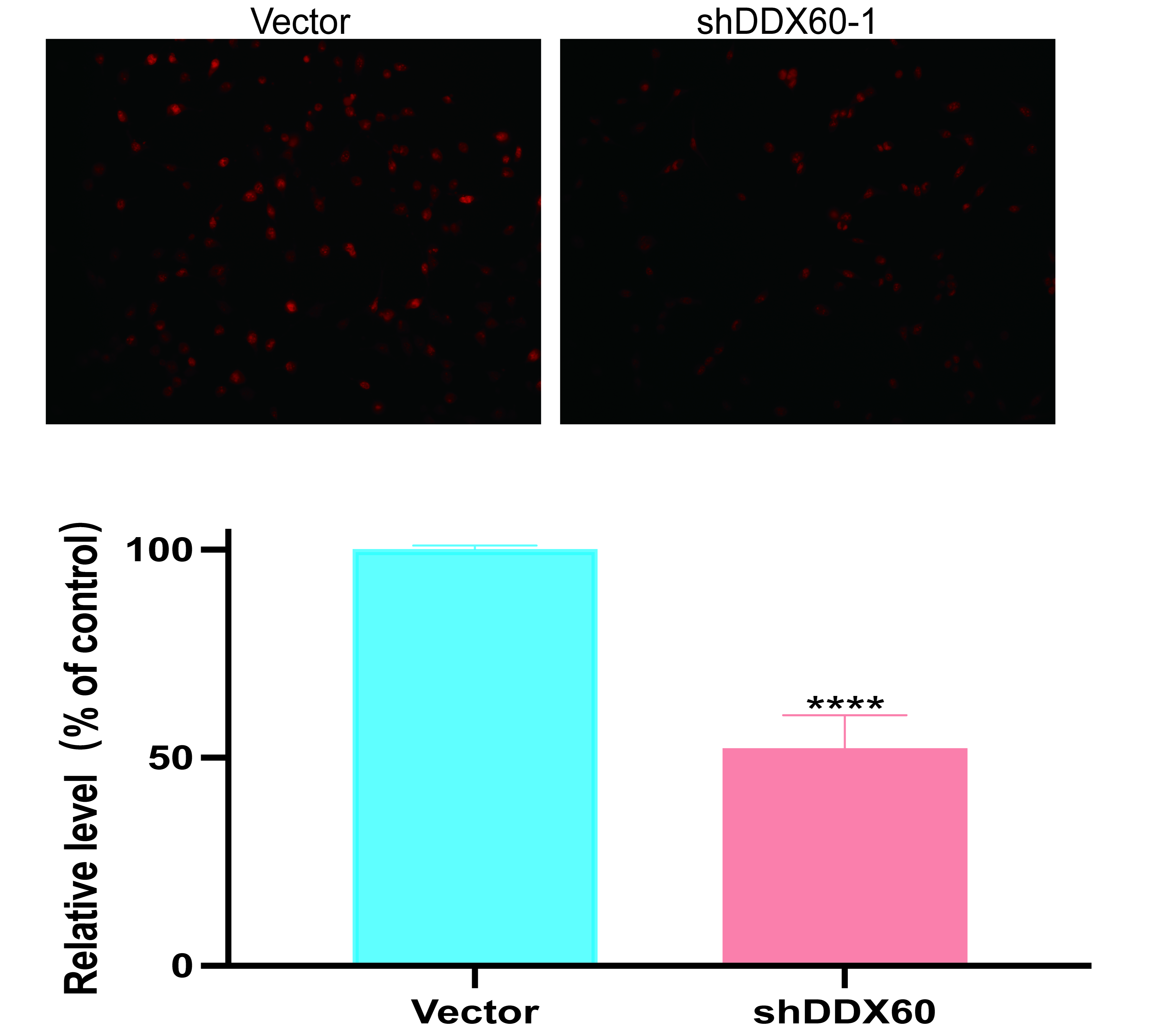

Supplement: Supplementary file 14 — Supplementary Material 14. [file 41065_2024_361_MOESM14_ESM.tif]

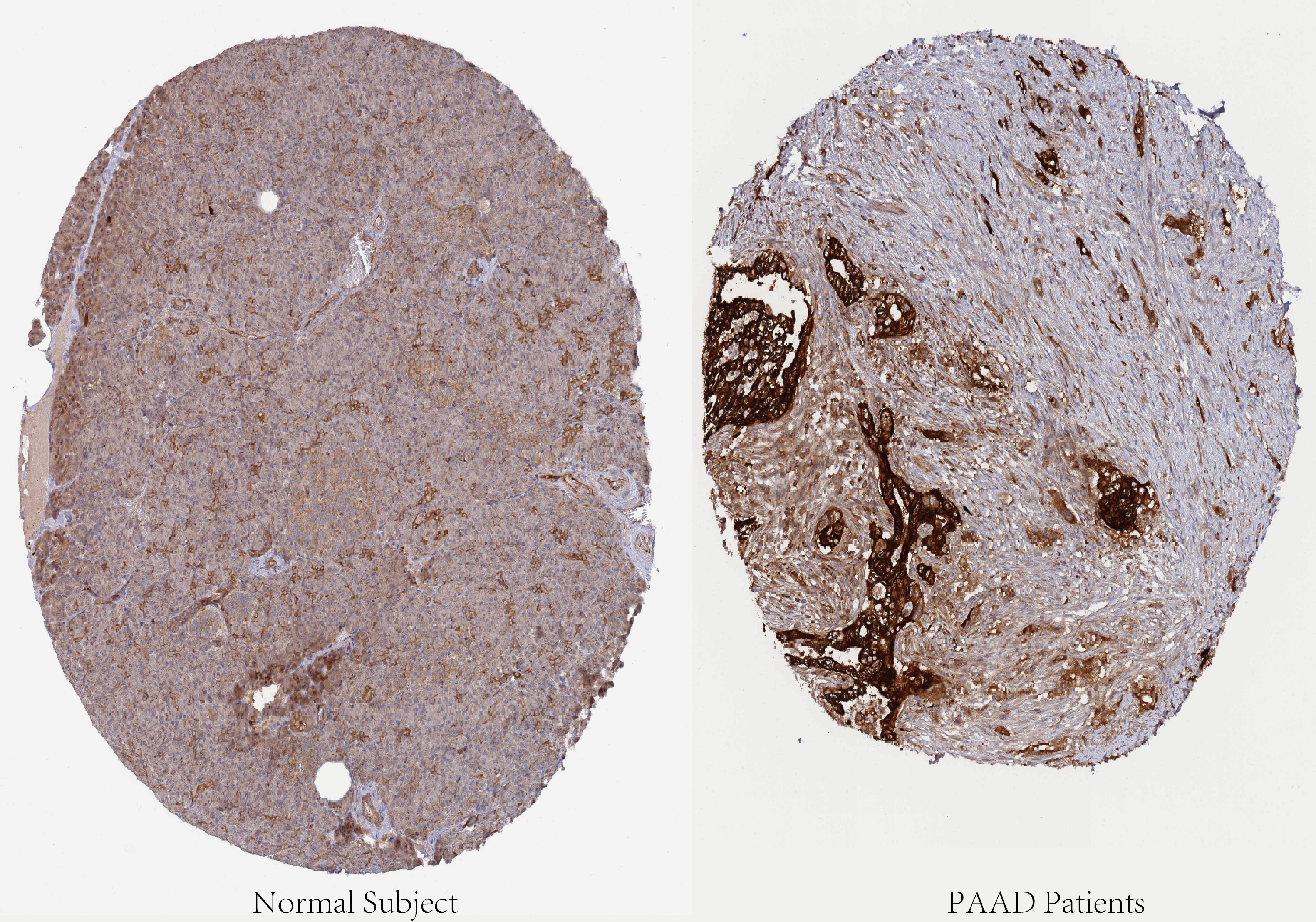

Supplement: Supplementary file 15 — Supplementary Material 15: Supplement Figure11. The IHC-based protein expression of DDX60 in PAAD tissues and normal pancreatic tissue. Normal subject information: Female, age 74, Patients id: 2162. HPA number: HPA046952. PAAD subject information: Female, age 73, Patient id: 3719. HPA number: HPA046952. [file 41065_2024_361_MOESM15_ESM.png]

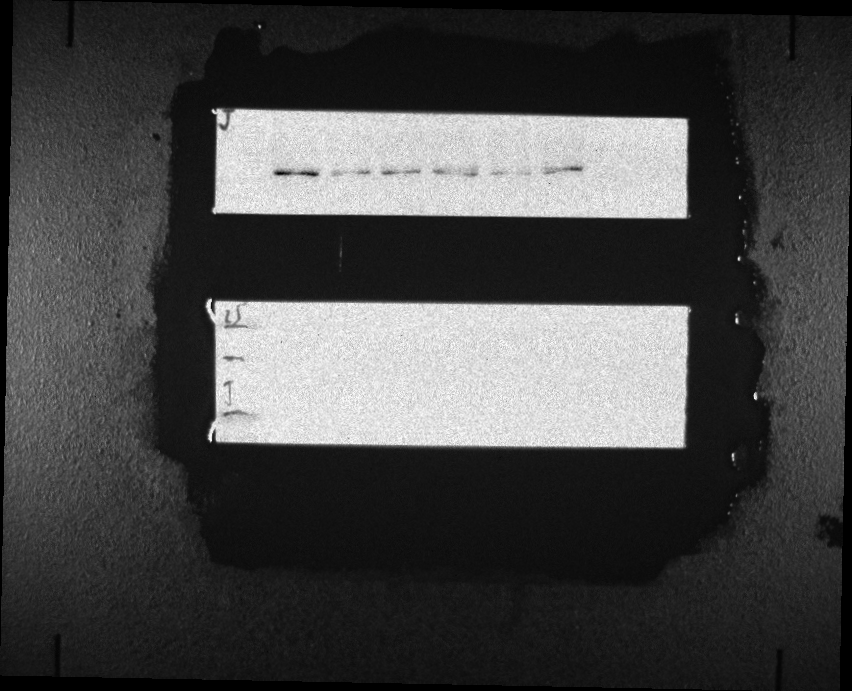


100kd


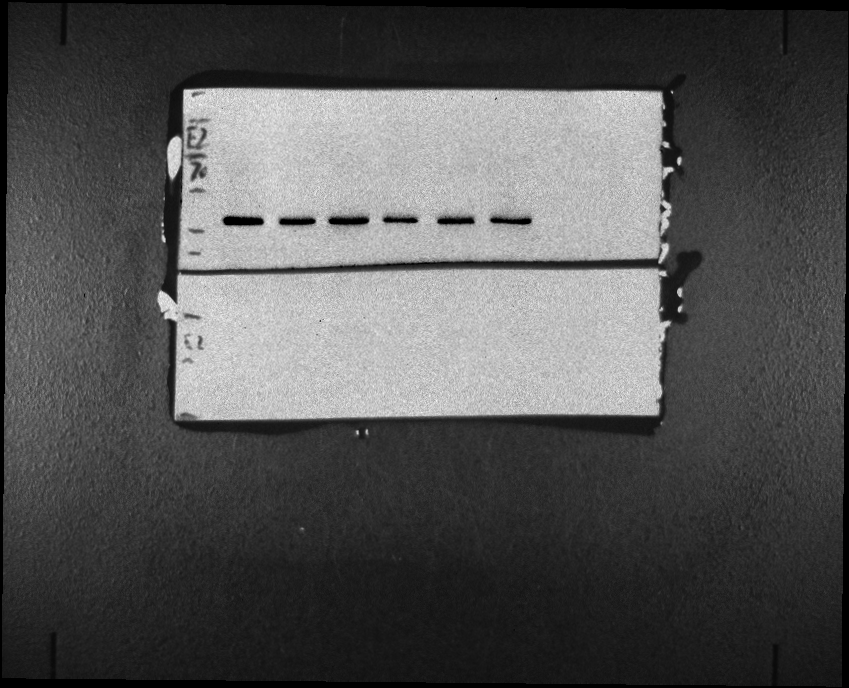


42kd

Supplement: Supplementary file 23 — Supplementary Material 23. [file 41065_2024_361_MOESM23_ESM.doc]
