# Supplementary material for: Deciphering the potential ability of DExD/H-box helicase 60 (DDX60) on the proliferation, diagnostic and prognostic biomarker in pancreatic cancer: a research based on silico, RNA-seq and molecular biology experiment
Source: Hereditas. 2025 Jan 22;162:6. doi: 10.1186/s41065-024-00361-9 (PMC11753068; doi:10.1186/s41065-024-00361-9)
Supplement: Supplementary file 16 — Supplementary Material 16: Supplement Table 1. The top ten GO and KEGG enrichment analysis of DEGs in GSE71729. [file 41065_2024_361_MOESM16_ESM.doc]

| **Supplement Table1.** The top ten GO and KEGG enrichment analysis of DEGs in GSE71729. | | | | |
| --- | --- | --- | --- | --- |
| Description | Term | Count | PValue | FDR |
| GOTERM_BP_DIRECT | GO:0007155~cell adhesion | 62 | 3.68E-12 | 1.36E-08 |
| GOTERM_BP_DIRECT | GO:0006508~proteolysis | 51 | 1.68E-11 | 3.12E-08 |
| GOTERM_BP_DIRECT | GO:0061844~antimicrobial humoral immune response mediated by antimicrobial peptide | 23 | 5.90E-10 | 7.28E-07 |
| GOTERM_BP_DIRECT | GO:0045926~negative regulation of growth | 11 | 9.39E-10 | 8.69E-07 |
| GOTERM_BP_DIRECT | GO:0030198~extracellular matrix organization | 26 | 2.03E-08 | 1.49E-05 |
| GOTERM_BP_DIRECT | GO:0098609~cell-cell adhesion | 28 | 2.52E-08 | 1.49E-05 |
| GOTERM_BP_DIRECT | GO:0007267~cell-cell signaling | 31 | 2.81E-08 | 1.49E-05 |
| GOTERM_BP_DIRECT | GO:0010273~detoxification of copper ion | 9 | 8.11E-08 | 3.52E-05 |
| GOTERM_BP_DIRECT | GO:0006954~inflammatory response | 43 | 8.55E-08 | 3.52E-05 |
| GOTERM_BP_DIRECT | GO:0035987~endodermal cell differentiation | 11 | 2.86E-07 | 1.06E-04 |
| GOTERM_CC_DIRECT | GO:0005615~extracellular space | 226 | 3.54E-50 | 1.76E-47 |
| GOTERM_CC_DIRECT | GO:0005576~extracellular region | 224 | 3.72E-42 | 9.26E-40 |
| GOTERM_CC_DIRECT | GO:0070062~extracellular exosome | 192 | 1.23E-24 | 2.05E-22 |
| GOTERM_CC_DIRECT | GO:0009986~cell surface | 79 | 3.48E-18 | 4.33E-16 |
| GOTERM_CC_DIRECT | GO:0031012~extracellular matrix | 40 | 2.03E-12 | 2.02E-10 |
| GOTERM_CC_DIRECT | GO:0016324~apical plasma membrane | 48 | 1.34E-11 | 1.11E-09 |
| GOTERM_CC_DIRECT | GO:0005886~plasma membrane | 293 | 2.89E-09 | 2.06E-07 |
| GOTERM_CC_DIRECT | GO:0005788~endoplasmic reticulum lumen | 37 | 1.29E-08 | 8.04E-07 |
| GOTERM_CC_DIRECT | GO:0001533~cornified envelope | 15 | 9.80E-08 | 5.42E-06 |
| GOTERM_CC_DIRECT | GO:0016323~basolateral plasma membrane | 30 | 2.68E-07 | 1.33E-05 |
| GOTERM_MF_DIRECT | GO:0005201~extracellular matrix structural constituent | 30 | 1.22E-12 | 9.72E-10 |
| GOTERM_MF_DIRECT | GO:0005509~calcium ion binding | 78 | 2.03E-12 | 9.72E-10 |
| GOTERM_MF_DIRECT | GO:0004252~serine-type endopeptidase activity | 35 | 2.89E-12 | 9.72E-10 |
| GOTERM_MF_DIRECT | GO:0005102~receptor binding | 48 | 4.83E-10 | 1.22E-07 |
| GOTERM_MF_DIRECT | GO:0008201~heparin binding | 25 | 6.99E-07 | 1.41E-04 |
| GOTERM_MF_DIRECT | GO:0030020~extracellular matrix structural constituent conferring tensile strength | 12 | 9.47E-07 | 1.59E-04 |
| GOTERM_MF_DIRECT | GO:0005178~integrin binding | 23 | 1.95E-06 | 2.63E-04 |
| GOTERM_MF_DIRECT | GO:0004867~serine-type endopeptidase inhibitor activity | 18 | 2.09E-06 | 2.63E-04 |
| GOTERM_MF_DIRECT | GO:0004601~peroxidase activity | 11 | 2.76E-06 | 3.09E-04 |
| GOTERM_MF_DIRECT | GO:0008009~chemokine activity | 12 | 7.83E-06 | 7.89E-04 |
| KEGG_PATHWAY | hsa04974:Protein digestion and absorption | 29 | 9.32E-14 | 2.54E-11 |
| KEGG_PATHWAY | hsa04972:Pancreatic secretion | 26 | 2.51E-11 | 3.41E-09 |
| KEGG_PATHWAY | hsa04978:Mineral absorption | 16 | 1.96E-07 | 1.78E-05 |
| KEGG_PATHWAY | hsa04512:ECM-receptor interaction | 18 | 1.59E-06 | 1.08E-04 |
| KEGG_PATHWAY | hsa00982:Drug metabolism - cytochrome P450 | 14 | 5.71E-05 | 0.003103541 |
| KEGG_PATHWAY | hsa04510:Focal adhesion | 25 | 7.85E-05 | 0.003444204 |
| KEGG_PATHWAY | hsa04918:Thyroid hormone synthesis | 14 | 8.86E-05 | 0.003444204 |
| KEGG_PATHWAY | hsa04971:Gastric acid secretion | 14 | 1.02E-04 | 0.003470981 |
| KEGG_PATHWAY | hsa00830:Retinol metabolism | 13 | 1.38E-04 | 0.003562237 |
| KEGG_PATHWAY | hsa04976:Bile secretion | 15 | 1.42E-04 | 0.003562237 |
