# Supplementary material for: Deciphering the potential ability of DExD/H-box helicase 60 (DDX60) on the proliferation, diagnostic and prognostic biomarker in pancreatic cancer: a research based on silico, RNA-seq and molecular biology experiment
Source: Hereditas. 2025 Jan 22;162:6. doi: 10.1186/s41065-024-00361-9 (PMC11753068; doi:10.1186/s41065-024-00361-9)
Supplement: Supplementary file 17 — Supplementary Material 17: Supplement Table 2. The top ten GO and KEGG enrichment analysis of DEGs in GSE183795. [file 41065_2024_361_MOESM17_ESM.doc]

| **Supplement Table2.** The top ten GO and KEGG enrichment analysis of DEGs in GSE183795. | | | | |
| --- | --- | --- | --- | --- |
| Description | Term | Count | PValue | FDR |
| GOTERM_BP_DIRECT | GO:0007155~cell adhesion | 62 | 2.30E-18 | 6.90E-15 |
| GOTERM_BP_DIRECT | GO:0030199~collagen fibril organization | 20 | 7.67E-15 | 1.15E-11 |
| GOTERM_BP_DIRECT | GO:0098609~cell-cell adhesion | 26 | 9.14E-10 | 9.12E-07 |
| GOTERM_BP_DIRECT | GO:0030198~extracellular matrix organization | 23 | 6.61E-09 | 4.95E-06 |
| GOTERM_BP_DIRECT | GO:0006508~proteolysis | 37 | 2.40E-08 | 1.44E-05 |
| GOTERM_BP_DIRECT | GO:0042060~wound healing | 17 | 8.89E-08 | 4.44E-05 |
| GOTERM_BP_DIRECT | GO:0007160~cell-matrix adhesion | 17 | 1.53E-07 | 6.56E-05 |
| GOTERM_BP_DIRECT | GO:0009615~response to virus | 17 | 2.58E-07 | 9.64E-05 |
| GOTERM_BP_DIRECT | GO:0043434~response to peptide hormone | 12 | 8.34E-07 | 2.58E-04 |
| GOTERM_BP_DIRECT | GO:0001666~response to hypoxia | 21 | 8.61E-07 | 2.58E-04 |
| GOTERM_CC_DIRECT | GO:0005615~extracellular space | 180 | 8.67E-46 | 3.67E-43 |
| GOTERM_CC_DIRECT | GO:0005576~extracellular region | 179 | 1.72E-39 | 3.64E-37 |
| GOTERM_CC_DIRECT | GO:0070062~extracellular exosome | 172 | 2.55E-33 | 3.59E-31 |
| GOTERM_CC_DIRECT | GO:0009986~cell surface | 71 | 2.31E-21 | 2.45E-19 |
| GOTERM_CC_DIRECT | GO:0031012~extracellular matrix | 41 | 9.08E-18 | 7.68E-16 |
| GOTERM_CC_DIRECT | GO:0005886~plasma membrane | 248 | 8.47E-16 | 5.97E-14 |
| GOTERM_CC_DIRECT | GO:0005788~endoplasmic reticulum lumen | 38 | 4.30E-13 | 2.42E-11 |
| GOTERM_CC_DIRECT | GO:0005887~integral component of plasma membrane | 94 | 4.57E-13 | 2.42E-11 |
| GOTERM_CC_DIRECT | GO:0016324~apical plasma membrane | 41 | 4.28E-12 | 2.01E-10 |
| GOTERM_CC_DIRECT | GO:0005581~collagen trimer | 18 | 1.55E-09 | 6.54E-08 |
| GOTERM_MF_DIRECT | GO:0005201~extracellular matrix structural constituent | 37 | 2.94E-23 | 2.52E-20 |
| GOTERM_MF_DIRECT | GO:0005178~integrin binding | 30 | 2.41E-14 | 1.03E-11 |
| GOTERM_MF_DIRECT | GO:0005518~collagen binding | 17 | 2.56E-10 | 7.33E-08 |
| GOTERM_MF_DIRECT | GO:0004252~serine-type endopeptidase activity | 26 | 2.26E-09 | 4.86E-07 |
| GOTERM_MF_DIRECT | GO:0005509~calcium ion binding | 54 | 3.91E-08 | 6.71E-06 |
| GOTERM_MF_DIRECT | GO:0004867~serine-type endopeptidase inhibitor activity | 17 | 1.41E-07 | 2.01E-05 |
| GOTERM_MF_DIRECT | GO:0070492~oligosaccharide binding | 6 | 3.22E-05 | 0.003845594 |
| GOTERM_MF_DIRECT | GO:0030020~extracellular matrix structural constituent conferring tensile strength | 9 | 3.59E-05 | 0.003845594 |
| GOTERM_MF_DIRECT | GO:0002020~protease binding | 14 | 4.80E-05 | 0.004384111 |
| GOTERM_MF_DIRECT | GO:0001968~fibronectin binding | 8 | 5.11E-05 | 0.004384111 |
| KEGG_PATHWAY | hsa04512:ECM-receptor interaction | 22 | 2.00E-11 | 5.51E-09 |
| KEGG_PATHWAY | hsa04972:Pancreatic secretion | 22 | 3.91E-10 | 4.35E-08 |
| KEGG_PATHWAY | hsa04974:Protein digestion and absorption | 22 | 4.73E-10 | 4.35E-08 |
| KEGG_PATHWAY | hsa04510:Focal adhesion | 27 | 1.00E-07 | 6.90E-06 |
| KEGG_PATHWAY | hsa04610:Complement and coagulation cascades | 15 | 6.73E-06 | 3.72E-04 |
| KEGG_PATHWAY | hsa04151:PI3K-Akt signaling pathway | 31 | 7.49E-05 | 0.003445741 |
| KEGG_PATHWAY | hsa01230:Biosynthesis of amino acids | 11 | 7.46E-04 | 0.029402061 |
| KEGG_PATHWAY | hsa05412:Arrhythmogenic right ventricular cardiomyopathy | 11 | 9.20E-04 | 0.031725672 |
| KEGG_PATHWAY | hsa04810:Regulation of actin cytoskeleton | 20 | 0.002003766 | 0.061448833 |
| KEGG_PATHWAY | hsa05146:Amoebiasis | 12 | 0.00242876 | 0.067033783 |
