# Supplementary material for: Deciphering the potential ability of DExD/H-box helicase 60 (DDX60) on the proliferation, diagnostic and prognostic biomarker in pancreatic cancer: a research based on silico, RNA-seq and molecular biology experiment
Source: Hereditas. 2025 Jan 22;162:6. doi: 10.1186/s41065-024-00361-9 (PMC11753068; doi:10.1186/s41065-024-00361-9)
Supplement: Supplementary file 18 — Supplementary Material 18: Supplement Table 3. The top ten GO and KEGG enrichment analysis of DEGs in GSE16515. [file 41065_2024_361_MOESM18_ESM.doc]

| **Supplement Table3.** The top ten GO and KEGG enrichment analysis of DEGs in GSE16515. | | | | |
| --- | --- | --- | --- | --- |
| Description | Term | Count | PValue | FDR |
| GOTERM_BP_DIRECT | GO:0007155~cell adhesion | 120 | 1.21E-15 | 7.63E-12 |
| GOTERM_BP_DIRECT | GO:0016477~cell migration | 71 | 1.03E-13 | 3.26E-10 |
| GOTERM_BP_DIRECT | GO:0098609~cell-cell adhesion | 56 | 4.02E-13 | 8.44E-10 |
| GOTERM_BP_DIRECT | GO:0043066~negative regulation of apoptotic process | 107 | 4.83E-12 | 6.88E-09 |
| GOTERM_BP_DIRECT | GO:0009615~response to virus | 39 | 5.45E-12 | 6.88E-09 |
| GOTERM_BP_DIRECT | GO:0007165~signal transduction | 201 | 7.23E-11 | 7.60E-08 |
| GOTERM_BP_DIRECT | GO:0008284~positive regulation of cell proliferation | 106 | 1.50E-10 | 1.35E-07 |
| GOTERM_BP_DIRECT | GO:0042493~response to drug | 67 | 3.44E-10 | 2.71E-07 |
| GOTERM_BP_DIRECT | GO:0030198~extracellular matrix organization | 46 | 3.86E-10 | 2.71E-07 |
| GOTERM_BP_DIRECT | GO:0051301~cell division | 80 | 5.82E-10 | 3.67E-07 |
| GOTERM_CC_DIRECT | GO:0070062~extracellular exosome | 455 | 8.44E-58 | 7.12E-55 |
| GOTERM_CC_DIRECT | GO:0005576~extracellular region | 368 | 3.99E-29 | 1.68E-26 |
| GOTERM_CC_DIRECT | GO:0005615~extracellular space | 339 | 1.08E-27 | 3.05E-25 |
| GOTERM_CC_DIRECT | GO:0005886~plasma membrane | 714 | 3.66E-23 | 7.73E-21 |
| GOTERM_CC_DIRECT | GO:0009986~cell surface | 147 | 1.12E-22 | 1.88E-20 |
| GOTERM_CC_DIRECT | GO:0005737~cytoplasm | 739 | 5.59E-22 | 7.86E-20 |
| GOTERM_CC_DIRECT | GO:0005925~focal adhesion | 106 | 2.28E-19 | 2.75E-17 |
| GOTERM_CC_DIRECT | GO:0005829~cytosol | 716 | 5.70E-19 | 6.01E-17 |
| GOTERM_CC_DIRECT | GO:0016020~membrane | 524 | 1.60E-18 | 1.50E-16 |
| GOTERM_CC_DIRECT | GO:0016324~apical plasma membrane | 93 | 2.60E-16 | 2.20E-14 |
| GOTERM_MF_DIRECT | GO:0005515~protein binding | 1508 | 4.27E-20 | 7.63E-17 |
| GOTERM_MF_DIRECT | GO:0042802~identical protein binding | 288 | 2.32E-16 | 2.08E-13 |
| GOTERM_MF_DIRECT | GO:0005178~integrin binding | 51 | 8.63E-13 | 5.14E-10 |
| GOTERM_MF_DIRECT | GO:0045296~cadherin binding | 75 | 2.66E-11 | 1.07E-08 |
| GOTERM_MF_DIRECT | GO:0005201~extracellular matrix structural constituent | 44 | 2.98E-11 | 1.07E-08 |
| GOTERM_MF_DIRECT | GO:0002020~protease binding | 38 | 7.98E-11 | 2.38E-08 |
| GOTERM_MF_DIRECT | GO:0003779~actin binding | 75 | 8.22E-10 | 2.10E-07 |
| GOTERM_MF_DIRECT | GO:0005509~calcium ion binding | 132 | 3.80E-09 | 8.50E-07 |
| GOTERM_MF_DIRECT | GO:0051015~actin filament binding | 55 | 5.97E-09 | 1.19E-06 |
| GOTERM_MF_DIRECT | GO:0004252~serine-type endopeptidase activity | 49 | 1.05E-08 | 1.89E-06 |
| KEGG_PATHWAY | hsa05200:Pathways in cancer | 108 | 2.96E-07 | 6.23E-05 |
| KEGG_PATHWAY | hsa04510:Focal adhesion | 52 | 5.23E-07 | 6.23E-05 |
| KEGG_PATHWAY | hsa04974:Protein digestion and absorption | 33 | 6.55E-07 | 6.23E-05 |
| KEGG_PATHWAY | hsa04670:Leukocyte transendothelial migration | 35 | 8.56E-07 | 6.23E-05 |
| KEGG_PATHWAY | hsa04972:Pancreatic secretion | 32 | 1.66E-06 | 7.97E-05 |
| KEGG_PATHWAY | hsa05146:Amoebiasis | 32 | 1.66E-06 | 7.97E-05 |
| KEGG_PATHWAY | hsa04512:ECM-receptor interaction | 29 | 1.92E-06 | 7.97E-05 |
| KEGG_PATHWAY | hsa05205:Proteoglycans in cancer | 50 | 5.50E-06 | 2.00E-04 |
| KEGG_PATHWAY | hsa04810:Regulation of actin cytoskeleton | 54 | 6.34E-06 | 2.05E-04 |
| KEGG_PATHWAY | hsa05132:Salmonella infection | 57 | 8.73E-06 | 2.38E-04 |
