# Supplementary material for: Deciphering the potential ability of DExD/H-box helicase 60 (DDX60) on the proliferation, diagnostic and prognostic biomarker in pancreatic cancer: a research based on silico, RNA-seq and molecular biology experiment
Source: Hereditas. 2025 Jan 22;162:6. doi: 10.1186/s41065-024-00361-9 (PMC11753068; doi:10.1186/s41065-024-00361-9)
Supplement: Supplementary file 19 — Supplementary Material 19: Supplement Table 4. The top ten GO and KEGG enrichment analysis of DEGs in GSE28735. [file 41065_2024_361_MOESM19_ESM.doc]

| **Supplement Table4.** The top ten GO and KEGG enrichment analysis of DEGs in GSE28735. | | | | |
| --- | --- | --- | --- | --- |
| Description | Term | Count | PValue | FDR |
| GOTERM_BP_DIRECT | GO:0007155~cell adhesion | 75 | 1.51E-17 | 5.89E-14 |
| GOTERM_BP_DIRECT | GO:0030198~extracellular matrix organization | 33 | 1.58E-12 | 3.10E-09 |
| GOTERM_BP_DIRECT | GO:0030199~collagen fibril organization | 20 | 6.32E-12 | 8.24E-09 |
| GOTERM_BP_DIRECT | GO:0006508~proteolysis | 50 | 6.33E-10 | 5.54E-07 |
| GOTERM_BP_DIRECT | GO:0042060~wound healing | 23 | 7.08E-10 | 5.54E-07 |
| GOTERM_BP_DIRECT | GO:0035987~endodermal cell differentiation | 13 | 3.64E-09 | 2.37E-06 |
| GOTERM_BP_DIRECT | GO:0098609~cell-cell adhesion | 30 | 7.12E-09 | 3.98E-06 |
| GOTERM_BP_DIRECT | GO:0016477~cell migration | 36 | 1.57E-08 | 7.69E-06 |
| GOTERM_BP_DIRECT | GO:0048146~positive regulation of fibroblast proliferation | 16 | 2.36E-08 | 1.02E-05 |
| GOTERM_BP_DIRECT | GO:0007160~cell-matrix adhesion | 21 | 4.62E-08 | 1.81E-05 |
| GOTERM_CC_DIRECT | GO:0005615~extracellular space | 219 | 1.35E-41 | 6.87E-39 |
| GOTERM_CC_DIRECT | GO:0005576~extracellular region | 227 | 3.78E-39 | 9.65E-37 |
| GOTERM_CC_DIRECT | GO:0070062~extracellular exosome | 217 | 9.94E-32 | 1.69E-29 |
| GOTERM_CC_DIRECT | GO:0031012~extracellular matrix | 53 | 1.08E-20 | 1.38E-18 |
| GOTERM_CC_DIRECT | GO:0005886~plasma membrane | 352 | 6.61E-20 | 5.92E-18 |
| GOTERM_CC_DIRECT | GO:0009986~cell surface | 85 | 6.97E-20 | 5.92E-18 |
| GOTERM_CC_DIRECT | GO:0005788~endoplasmic reticulum lumen | 49 | 9.51E-15 | 6.93E-13 |
| GOTERM_CC_DIRECT | GO:0016324~apical plasma membrane | 55 | 1.44E-14 | 9.16E-13 |
| GOTERM_CC_DIRECT | GO:0005887~integral component of plasma membrane | 117 | 3.05E-11 | 1.73E-09 |
| GOTERM_CC_DIRECT | GO:0005604~basement membrane | 23 | 8.46E-11 | 4.31E-09 |
| GOTERM_MF_DIRECT | GO:0005201~extracellular matrix structural constituent | 43 | 9.83E-24 | 1.08E-20 |
| GOTERM_MF_DIRECT | GO:0004252~serine-type endopeptidase activity | 37 | 3.65E-13 | 2.01E-10 |
| GOTERM_MF_DIRECT | GO:0005178~integrin binding | 32 | 6.72E-12 | 2.46E-09 |
| GOTERM_MF_DIRECT | GO:0005518~collagen binding | 21 | 1.12E-11 | 3.09E-09 |
| GOTERM_MF_DIRECT | GO:0005509~calcium ion binding | 76 | 9.70E-11 | 2.13E-08 |
| GOTERM_MF_DIRECT | GO:0004867~serine-type endopeptidase inhibitor activity | 20 | 1.63E-07 | 2.56E-05 |
| GOTERM_MF_DIRECT | GO:0030020~extracellular matrix structural constituent conferring tensile strength | 13 | 1.63E-07 | 2.56E-05 |
| GOTERM_MF_DIRECT | GO:0042802~identical protein binding | 117 | 8.55E-06 | 0.001173991 |
| GOTERM_MF_DIRECT | GO:0002020~protease binding | 18 | 1.02E-05 | 0.001242883 |
| GOTERM_MF_DIRECT | GO:0005102~receptor binding | 39 | 1.21E-05 | 0.001331241 |
| KEGG_PATHWAY | hsa04974:Protein digestion and absorption | 29 | 1.18E-12 | 3.32E-10 |
| KEGG_PATHWAY | hsa04512:ECM-receptor interaction | 26 | 6.75E-12 | 9.44E-10 |
| KEGG_PATHWAY | hsa04972:Pancreatic secretion | 27 | 3.81E-11 | 3.55E-09 |
| KEGG_PATHWAY | hsa04510:Focal adhesion | 32 | 2.40E-07 | 1.68E-05 |
| KEGG_PATHWAY | hsa04151:PI3K-Akt signaling pathway | 41 | 1.66E-05 | 9.31E-04 |
| KEGG_PATHWAY | hsa05205:Proteoglycans in cancer | 28 | 2.94E-05 | 0.001373731 |
| KEGG_PATHWAY | hsa00830:Retinol metabolism | 14 | 8.77E-05 | 0.003508828 |
| KEGG_PATHWAY | hsa00010:Glycolysis / Gluconeogenesis | 13 | 3.10E-04 | 0.010851278 |
| KEGG_PATHWAY | hsa05146:Amoebiasis | 16 | 5.43E-04 | 0.016888179 |
| KEGG_PATHWAY | hsa00982:Drug metabolism - cytochrome P450 | 13 | 6.15E-04 | 0.017218149 |
