# Supplementary material for: Deciphering the potential ability of DExD/H-box helicase 60 (DDX60) on the proliferation, diagnostic and prognostic biomarker in pancreatic cancer: a research based on silico, RNA-seq and molecular biology experiment
Source: Hereditas. 2025 Jan 22;162:6. doi: 10.1186/s41065-024-00361-9 (PMC11753068; doi:10.1186/s41065-024-00361-9)
Supplement: Supplementary file 20 — Supplementary Material 20: Supplement Table 5. The top ten GO and KEGG enrichment analysis of DEGs in GSE62452. [file 41065_2024_361_MOESM20_ESM.doc]

| **Supplement Table5.** The top ten GO and KEGG enrichment analysis of DEGs in GSE62452. | | | | |
| --- | --- | --- | --- | --- |
| Description | Term | Count | PValue | FDR |
| GOTERM_BP_DIRECT | GO:0007155~cell adhesion | 69 | 7.72E-20 | 2.58E-16 |
| GOTERM_BP_DIRECT | GO:0006508~proteolysis | 49 | 3.69E-13 | 6.17E-10 |
| GOTERM_BP_DIRECT | GO:0030199~collagen fibril organization | 19 | 1.06E-12 | 1.18E-09 |
| GOTERM_BP_DIRECT | GO:0030198~extracellular matrix organization | 27 | 1.11E-10 | 9.29E-08 |
| GOTERM_BP_DIRECT | GO:0035987~endodermal cell differentiation | 12 | 3.76E-09 | 2.52E-06 |
| GOTERM_BP_DIRECT | GO:0007160~cell-matrix adhesion | 19 | 2.89E-08 | 1.61E-05 |
| GOTERM_BP_DIRECT | GO:0030335~positive regulation of cell migration | 29 | 1.75E-07 | 8.35E-05 |
| GOTERM_BP_DIRECT | GO:0043434~response to peptide hormone | 13 | 3.94E-07 | 1.65E-04 |
| GOTERM_BP_DIRECT | GO:0042060~wound healing | 17 | 5.23E-07 | 1.95E-04 |
| GOTERM_BP_DIRECT | GO:0098609~cell-cell adhesion | 23 | 9.07E-07 | 3.04E-04 |
| GOTERM_CC_DIRECT | GO:0005615~extracellular space | 191 | 7.32E-44 | 3.32E-41 |
| GOTERM_CC_DIRECT | GO:0005576~extracellular region | 189 | 6.66E-37 | 1.51E-34 |
| GOTERM_CC_DIRECT | GO:0070062~extracellular exosome | 189 | 1.63E-34 | 2.46E-32 |
| GOTERM_CC_DIRECT | GO:0005886~plasma membrane | 290 | 9.25E-21 | 1.05E-18 |
| GOTERM_CC_DIRECT | GO:0009986~cell surface | 74 | 4.28E-20 | 3.88E-18 |
| GOTERM_CC_DIRECT | GO:0031012~extracellular matrix | 46 | 9.29E-20 | 7.02E-18 |
| GOTERM_CC_DIRECT | GO:0016324~apical plasma membrane | 50 | 6.51E-16 | 4.22E-14 |
| GOTERM_CC_DIRECT | GO:0005887~integral component of plasma membrane | 100 | 4.21E-12 | 2.17E-10 |
| GOTERM_CC_DIRECT | GO:0005788~endoplasmic reticulum lumen | 39 | 4.31E-12 | 2.17E-10 |
| GOTERM_CC_DIRECT | GO:0005604~basement membrane | 22 | 5.31E-12 | 2.40E-10 |
| GOTERM_MF_DIRECT | GO:0005201~extracellular matrix structural constituent | 38 | 2.03E-22 | 1.89E-19 |
| GOTERM_MF_DIRECT | GO:0005178~integrin binding | 32 | 1.40E-14 | 6.52E-12 |
| GOTERM_MF_DIRECT | GO:0004252~serine-type endopeptidase activity | 34 | 7.01E-14 | 2.18E-11 |
| GOTERM_MF_DIRECT | GO:0005509~calcium ion binding | 68 | 5.44E-12 | 1.27E-09 |
| GOTERM_MF_DIRECT | GO:0005518~collagen binding | 19 | 1.83E-11 | 3.41E-09 |
| GOTERM_MF_DIRECT | GO:0002020~protease binding | 19 | 8.09E-08 | 1.26E-05 |
| GOTERM_MF_DIRECT | GO:0030020~extracellular matrix structural constituent conferring tensile strength | 12 | 1.38E-07 | 1.83E-05 |
| GOTERM_MF_DIRECT | GO:0042802~identical protein binding | 102 | 4.81E-07 | 5.61E-05 |
| GOTERM_MF_DIRECT | GO:0001968~fibronectin binding | 9 | 1.29E-05 | 0.001330984 |
| GOTERM_MF_DIRECT | GO:0004867~serine-type endopeptidase inhibitor activity | 15 | 1.83E-05 | 0.001708383 |
| KEGG_PATHWAY | hsa04974:Protein digestion and absorption | 27 | 4.10E-13 | 1.13E-10 |
| KEGG_PATHWAY | hsa04512:ECM-receptor interaction | 23 | 3.40E-11 | 4.69E-09 |
| KEGG_PATHWAY | hsa04972:Pancreatic secretion | 24 | 1.16E-10 | 1.07E-08 |
| KEGG_PATHWAY | hsa04510:Focal adhesion | 29 | 1.10E-07 | 7.56E-06 |
| KEGG_PATHWAY | hsa04610:Complement and coagulation cascades | 14 | 1.29E-04 | 0.007115473 |
| KEGG_PATHWAY | hsa04151:PI3K-Akt signaling pathway | 33 | 1.59E-04 | 0.007331078 |
| KEGG_PATHWAY | hsa01230:Biosynthesis of amino acids | 12 | 5.51E-04 | 0.021718388 |
| KEGG_PATHWAY | hsa05146:Amoebiasis | 14 | 7.15E-04 | 0.024666301 |
| KEGG_PATHWAY | hsa00830:Retinol metabolism | 11 | 9.56E-04 | 0.029314764 |
| KEGG_PATHWAY | hsa05205:Proteoglycans in cancer | 21 | 0.001079031 | 0.029781254 |
