# Supplementary material for: Deciphering the potential ability of DExD/H-box helicase 60 (DDX60) on the proliferation, diagnostic and prognostic biomarker in pancreatic cancer: a research based on silico, RNA-seq and molecular biology experiment
Source: Hereditas. 2025 Jan 22;162:6. doi: 10.1186/s41065-024-00361-9 (PMC11753068; doi:10.1186/s41065-024-00361-9)
Supplement: Supplementary file 21 — Supplementary Material 21: Supplement Table 6. Correlation analysis between DDX60 and 22 kinds of immune cells in pancreatic cancer (P＜0.05). [file 41065_2024_361_MOESM21_ESM.doc]

| **Supplement Table6.** Correlation analysis between DDX60 and 22 kinds of immune cells in pancreatic cancer (P＜0.05). | | |
| --- | --- | --- |
| Cell | cor | pvalue |
| Macrophages M1 | 0.312882431 | 0.004716567 |
| B cells naive | -0.290981973 | 0.00882954 |
| Dendritic cells activated | 0.259964274 | 0.019870236 |
| T cells regulatory (Tregs) | -0.257079231 | 0.021582143 |
