# Supplementary material for: Deciphering the potential ability of DExD/H-box helicase 60 (DDX60) on the proliferation, diagnostic and prognostic biomarker in pancreatic cancer: a research based on silico, RNA-seq and molecular biology experiment
Source: Hereditas. 2025 Jan 22;162:6. doi: 10.1186/s41065-024-00361-9 (PMC11753068; doi:10.1186/s41065-024-00361-9)
Supplement: Supplementary file 22 — Supplementary Material 22: Supplement Table 7. Correlation analysis between DDX60 and 24 kinds of immune checkpoint in pancreatic cancer (P＜0.05). [file 41065_2024_361_MOESM22_ESM.doc]

| **Supplement Table7.** Correlation analysis between DDX60 and 24 kinds of immune checkpoint in pancreatic cancer (P＜0.05). | | | |
| --- | --- | --- | --- |
| Biomarker | Gene | Cor | Pvalue |
| DDX60 | CD274 | 0.546325163 | 3.07E-15 |
| DDX60 | LGALS9 | 0.528682467 | 3.32E-14 |
| DDX60 | CD80 | 0.513965906 | 2.18E-13 |
| DDX60 | CD44 | 0.504864203 | 6.67E-13 |
| DDX60 | HAVCR2 | 0.449367015 | 3.14E-10 |
| DDX60 | CD86 | 0.443996068 | 5.38E-10 |
| DDX60 | TNFSF4 | 0.435331579 | 1.26E-09 |
| DDX60 | HHLA2 | 0.430683348 | 1.96E-09 |
| DDX60 | PDCD1LG2 | 0.430314861 | 2.03E-09 |
| DDX60 | NRP1 | 0.420537229 | 5.08E-09 |
| DDX60 | CD40 | 0.402290166 | 2.60E-08 |
| DDX60 | IDO1 | 0.382979523 | 1.32E-07 |
| DDX60 | TNFSF18 | 0.379430986 | 1.76E-07 |
| DDX60 | LAIR1 | 0.360373908 | 7.77E-07 |
| DDX60 | CD276 | 0.349170529 | 1.78E-06 |
| DDX60 | TNFSF15 | 0.336235351 | 4.46E-06 |
| DDX60 | TNFRSF9 | 0.302813371 | 3.98E-05 |
| DDX60 | TNFSF9 | 0.302271969 | 4.12E-05 |
| DDX60 | TNFRSF14 | 0.262933502 | 0.000391343 |
| DDX60 | CD244 | 0.260239685 | 0.000451126 |
| DDX60 | CD70 | 0.252730408 | 0.000665257 |
| DDX60 | CTLA4 | 0.25187633 | 0.000694799 |
| DDX60 | ICOS | 0.251868508 | 0.000695075 |
| DDX60 | TIGIT | 0.246603939 | 0.000905586 |
