# Supplementary material for: Deciphering the potential ability of DExD/H-box helicase 60 (DDX60) on the proliferation, diagnostic and prognostic biomarker in pancreatic cancer: a research based on silico, RNA-seq and molecular biology experiment
Source: Hereditas. 2025 Jan 22;162:6. doi: 10.1186/s41065-024-00361-9 (PMC11753068; doi:10.1186/s41065-024-00361-9)
Supplement: Supplementary file 24 — Supplementary Material 24. [file 41065_2024_361_MOESM24_ESM.doc]

**Single Cell Sequencing Analysis、Immune Analysis and Immune Checkpoint**

We used single cell datasets to analyze DDX60 and those biological functions at the cellular level. We use online databases Tumor Immune Single-cell Hub (TISCH, <http://tisch.comp-genomics.org/>) ^48^. The data set number included in this study are PAAD_CRA001160 ^49^, PAAD_GSE148673 ^50^, PAAD_GSE154778 ^51^, and PAAD_GSE165399 ^52^. We used four single cell sequencing data associated with pancreatic cancer to explore the potential relationship between DDX60 and tumor and immune cells. PAAD_CRA001160 contained 35 patients and 57,443 cells. PAAD_GSE148673 contained 2 patients and 6,196 cells. PAAD_GSE154778 contained 15 patients and 14,953 cells. PAAD_GSE165399 contained 3 patients and 9569 cells. Immune infiltration is becoming more and more popular. The occurrence and development of tumors are mostly related to immune activation or immunosuppression in vivo. This immune imbalance will disturb the ecological balance in the body. At the same time, there are many new ways of cancer treatment, such as tumor immunotherapy, PD-1 and PDL-1 drug use ^53-54^. There are a variety of immune checkpoints on the surface of tumor cells, which have a great influence on the effect of tumor immunotherapy. Some patients are sensitive to immunotherapy, and some patients are tolerant to immunotherapy. Therefore, it is particularly important to explore the sensitivity of people with special diseases to immunotherapy. Pearson test was used to test the correlation between DDX60 and 47 immune checkpoints.

**Single Cell Sequencing Analysis and Immune**

We first analyzed the distribution of DDX60 in different cell clusters at the single cell level. The analysis results of four single cell datasets showed that DDX60 had the most significant correlation with malignant cells. In PAAD_CRA001160 dataset、PAAD_GSE148673、PAAD_GSE154778 and PAAD_GSE165399, DDX60 has the best enrichment effect in pancreatic cancer malignant cells, and the expression level of DDX60 is positively correlated with malignancy. It is further confirmed that DDX60 plays the role of proto-oncogene in pancreatic cancer **(Supplement Figure2-5)**. From GSEA analysis, we know that DDX60 is involved in immune-related pathways in pancreatic cancer. High expression of DDX60 promotes immune infiltration of pancreatic tumors. TME usually occurs in a very complex tumor tissue microenvironment, which is mainly an immune microenvironment dominated by immune cells, and is related to tumor metastasis and invasion. Therefore, we explored an association between DDX60 pancreatic cancer and TME in the current study. The results showed that DDX60 expression was significantly positively correlated with Stromal score, Immune score and ESTIMATE score in TME **(Supplement Figure6)**. In addition, immune cell infiltration analysis showed that DDX60 expression was positively correlated with Macrophages M1 (cor=0.31; P=0.0047) and Dendritic cells activated (cor=0.26; P=0.0199). The expression of DDX60 was negatively correlated with B cells naive (cor=-0.29; P=0.0088) and T cells regulatory (Tregs) (cor=-0.26; P=0.0216) **(Supplement Figure7 and Supplement Table6)**. The above studies indicate that DDX60 interacts with immune-related cells in pancreatic cancer, which may activate the immune response of the human body. The expression of DDX60 was negatively correlated with T cells regulatory (Tregs) and B cells naive, which could explain the role of DDX60 as an oncogene in pancreatic cancer. Immune checkpoint is associated with immune escape and immune surveillance. We analyzed the correlation between DDX60 and immune checkpoint gene expression. The relationship between DDX60 and 47 immune checkpoints, we found that DDX60 was positively correlated with 24 immune checkpoints. Of particular interest were significant associations with immune checkpoint surface proteins such as CD274, LGALS9, CD80, and CD44 (P＜0.001) **(Supplement Figure8) (Supplement Table7)**, showing a possible mechanism of how DDX60-rich pancreatic cancer cells exert immune regulation.
